# Socio‐Emotional Development in Young Children With Cerebral Palsy: A Scoping Review

**DOI:** 10.1111/cch.70130

**Published:** 2025-07-02

**Authors:** Julie Enkebølle Hansen, Laura Lærkegård Støve, Mette Skovgaard Væver, Katrine Røhder

**Affiliations:** ^1^ Centre of Excellence in Early Intervention and Family Studies, Department of Psychology University of Copenhagen Copenhagen Denmark

**Keywords:** cerebral palsy, emotional development, methodological issues, social development, young children

## Abstract

**Background:**

Cerebral palsy (CP) is the most common physical disability in childhood, yet research on the socio‐emotional development of young children with or at high risk of CP remains limited. The aim of the study is to describe how socio‐emotional development has been investigated in young children diagnosed with or at high risk of CP and identify knowledge gaps and areas for future research.

**Methods:**

A scoping review based on a systematic search of PsycInfo, PubMed and Web of Science including studies on children aged 0–6:11 years diagnosed with or at high risk of CP, examining socio‐emotional development. No restrictions on publication year or language were applied. Data were extracted following PRISMA guidelines for scoping reviews.

**Results:**

Twenty‐five studies, representing 1914 toddlers and preschool children, were included. No studies exclusively examined infants. Most studies were cross‐sectional (68%) and used parent‐reported data (76%). The most commonly investigated areas of socio‐emotional development were expressing negative emotionality, engagement in peer relationships and exploratory behaviour. Less optimal socio‐emotional development was frequently reported, with prevalence estimates ranging from 14% to 65%. Socio‐emotional difficulties were associated with severity of child motor disability, child delayed language abilities and parenting difficulties.

**Conclusion:**

This review identifies conceptual and methodological limitations in studies of socio‐emotional development in young children with CP. Future research should include infants from a diverse range of geographical and cultural contexts, utilize observational or multi‐informant methods, investigate positive emotionality and parent–child relationships and adopt longitudinal designs to better capture if and how CP may impact socio‐emotional development in early childhood.

## Summary

- Socio‐emotional difficulties among young children with CP are commonly reported, with a wide range of prevalence rates found.
- The majority of existing studies are cross‐sectional, rely on parent‐reported information and are conducted among toddlers and preschool children living in Western high‐income countries.
- Conceptually, studies focus on negative emotionality, difficulties in peer relationships and exploratory behaviour, with fewer studies investigating positive emotionality and parent–child relationships.
- Risk factors for socio‐emotional development include child motor disability, child communication delays and parenting difficulties.
- Future studies should include more geographically and culturally diverse populations, be longitudinal and include observational and multi‐informant methodology (including child‐reported information).

**Abbreviations**
CP: cerebral palsy
QoL: quality of life
PRISMA: Preferred Reporting Items for Systematic Reviews and Meta‐Analyses
GMFCS: Gross Motor Function Classification System
CBCL: Child Behavior Checklist
PEDI: Pediatric Evaluation of Disability Inventory
TNO‐AZL: Preschool Children Quality of Life

## 1 Introduction

Cerebral palsy (CP) is the most common physical disability during childhood, affecting two to three in 1000 children in high‐ and low‐income countries, respectively (Kakooza‐Mwesige et al. 2017; Morgan et al. 2021). CP is defined as a neurodevelopmental disorder primarily affecting movement and posture, limiting motor activity due to nonprogressive disturbances in the developing foetal or infant brain (Rosenbaum et al. 2007). While adverse motor development is the hallmark of CP, more developmental domains are often also affected (Novak et al. 2012). Half (49%) of children with CP experience intellectual disability (Yin Foo et al. 2013) and three out of five (60%) children have language impairments with almost half of these children being nonverbal (Mei et al. 2016; Zhang et al. 2015). Other functions commonly affected are eating and drinking, vision, sleep and musculoskeletal health (Morgan et al. 2021). Less is known about how socio‐emotional development is affected in children with CP. This scoping review aims to map existing knowledge about socio‐emotional development in young children with CP to identify gaps in the research and areas for future research.

A systematic review and meta‐analysis from 2018 shows that approximately one out of three school‐aged children with CP experience mental health difficulties, with both social and emotional domains being affected (Downs et al. 2018). Specifically, peer problems (32%–87%), conduct problems (0%–34%), emotional problems (17%–30%) and hyperactivity (5%–28%) are areas of concern. Compared with prevalence rates of mental health problems among peers from the general population (10%–14%) (Downs et al. 2018), findings suggest that children and adolescents diagnosed with CP have a two to three times increased risk of experiencing mental health problems. A Danish register study confirms and expands these findings by estimating that the prevalence of being diagnosed with a psychiatric diagnosis (e.g., ADHD, autism spectrum disorder or affective disorder) is almost twice as high in 10‐ to 16‐year‐old children with CP compared with children without CP (11.6% vs. 6.2%) (Rackauskaite et al. 2020). Both studies conclude that mental health problems among school‐aged children and adolescents with CP are common and recommend systematically screening for such issues in paediatric follow‐up programmes (Downs et al. 2018). The studies by Downs et al. (2018) and Rackauskaite et al. (2020) focus on symptoms of or diagnosed mental health disorders, which may not encompass the broader domain of child socio‐emotional development. Furthermore, both studies relied on measures developed for typically developing children—for example, the Strength and Difficulties Questionnaire (Goodman 2001) and the Child Behaviour Checklist (Achenbach and Rescorla 2001)—as well as psychiatric diagnoses, which may be a limitation in relation to the assessment of children with neurodivergent development (Parkes et al. 2008). Furthermore, the results reported in the meta‐analysis by Downs et al. (2018) were solely based on parent‐reported information on child socio‐emotional development, which may be a limitation when studying subjective well‐being (Cummins 2002) where a combination of self‐report (when possible), multi‐informants and observational methodology is considered the golden standard (McConaughy and Whitcomb 2021).

These previous methodological limitations have also been addressed in a recent systematic review and meta‐analysis examining the broader concept of ‘quality of life’ (QoL) in children and adolescents (aged 2–17) diagnosed with CP (Makris et al. 2021). This meta‐analysis found varying results of emotional (e.g., positive affect and well‐being) and social (e.g., peer problems and behavioural difficulties) QoL depending on the measure being used. Some studies found that children with CP experienced more mental health problems and felt less accepted by their peers compared with typically developing children, while other studies failed to confirm these findings. Further, children tended to self‐report less socio‐emotional difficulties compared with parent ratings of the same (Makris et al. 2021).

Taken together, these studies show that while mental health problems among school‐aged children with CP are considered common, more knowledge is needed addressing methodological and conceptual differences in assessing socio‐emotional development in relation to CP and how these might affect findings. Particular attention should be given to differences in self‐ versus parent‐reported measures, the validity of measures developed for typically developing children versus children with neurodivergent development and conceptual issues related to the definition of socio‐emotional development (mental health, QoL, social functioning etc.). Finally, as existing reviews have focused mostly on studies including school‐aged children and adolescents, knowledge on socio‐emotional development specific to infants, toddlers and preschool children diagnosed with or at high risk for CP is needed.

Infant and early childhood socio‐emotional development is typically defined as the child's developing capacity ‘to form close and secure adult and peer relationships; experience, regulate, and express emotions in socially and culturally appropriate ways; and explore the environment and learn – all in the context of family, community and culture’ (Yates et al. 2008). From birth and onwards, children's social and emotional development are intertwined domains that lay the foundation for later mental well‐being, social competence and educational achievement. For example, children who can identify, express and regulate their emotions are more capable of building positive and trusting relationships with others and are better equipped to learning, problem solving and coping with challenges (Yates et al. 2008). Studies among both typical and premature children have demonstrated that nonoptimal or atypical socio‐emotional development in infancy or toddlerhood can lead to social or emotional difficulties and mental health problems later in life (Groh et al. 2017; Montagna and Nosarti 2016). There is thus a potential for prevention of later mental health problems by supporting healthy socio‐emotional development, such as forming a secure attachment relationship with parents and acquiring good emotion regulation abilities, during the formative early years. An important first step is mapping existing knowledge on how socio‐emotional development in young children with or at high risk of CP has been investigated to identify knowledge gaps and point to areas for future research.

### 1.1 The Current Study

As existing research has pointed to conceptual and methodological differences in the field (Makris et al. 2021), this review aims to (1) describe how socio‐emotional development has been investigated in relation to CP, (2) identify which factors have been investigated in relation to socio‐emotional development and (3) point to knowledge gaps and areas for future research. A scoping review methodology is considered most suitable for this purpose (Munn et al. 2018; Munn et al. 2022). Because the two prior reviews and meta‐analyses (Downs et al. 2018; Makris et al. 2021) focused on school‐aged children and adolescents, this scoping review is restricted to studies examining infants, toddlers and preschool children diagnosed with (or at high risk of) CP. The following research questions will guide the review:

1. What characterizes existing studies?
2. What characterizes participants in the included studies?
3. What methodologies have been utilized?
4. How has socio‐emotional development been conceptualized in the studies?
5. What findings are reported?

## 2 Method

The review was conducted in accordance with the Joanna Briggs Institute methodology for scoping reviews (Peters et al. 2020; Pollock et al. 2023). The protocol was preregistered in Open Science Framework, February 24, 2022 (https://osf.io/wtec7). The Preferred Reporting Items for Systematic Reviews and Meta‐analyses extension for Scoping Reviews (PRISMA‐ScR) guided the reporting (see Supporting Information S1).

### 2.1 Eligibility Criteria

Inclusion criteria for the studies were based on the PCC framework (participants, concept, and context) (Peters et al. 2020). Participants should be (1) infants or children aged 0–6:11 years, (2) be diagnosed with CP or at ‘high risk of CP’ following clinical guidelines (Novak et al. 2017) and (3) studies should examine typical and/or adverse socio‐emotional development and report on empirical results as either a prevalence of socio‐emotional problems and/or factors impacting on socio‐emotional development. Studies with heterogeneous samples (including children with other disabilities or a broader age range) should present results regarding socio‐emotional development for subsamples of relevance for this review. Children from all contexts (e.g., hospital settings, homes and day care settings) and studies published in any year and language were included.

Exclusion criteria for studies were (1) not original studies (e.g., reviews, meta‐analysis or protocol papers) or (2) not peer‐reviewed (e.g., theses or book chapters).

### 2.2 Search Strategy

An explorative search of PsycInfo and PubMed was undertaken in October 2021 to identify articles on the topic. Titles, abstracts and index terms of relevant papers were used to develop a full search strategy (see Supporting Information S2). The search strategy was adapted for each included database. The final search was conducted in PsycInfo, PubMed and Web of Science in February 2022 and updated in April 2024. Unidentified studies were searched for in trial and open science registers (Open Science Framework and Prospero) and through personal communication to key researchers identified through the systematic search. The reference list of all included studies was screened for additional studies, and a citation search of included studies was performed in Web of Science and Google Scholar.

### 2.3 Study Selection

**FIGURE 1:**
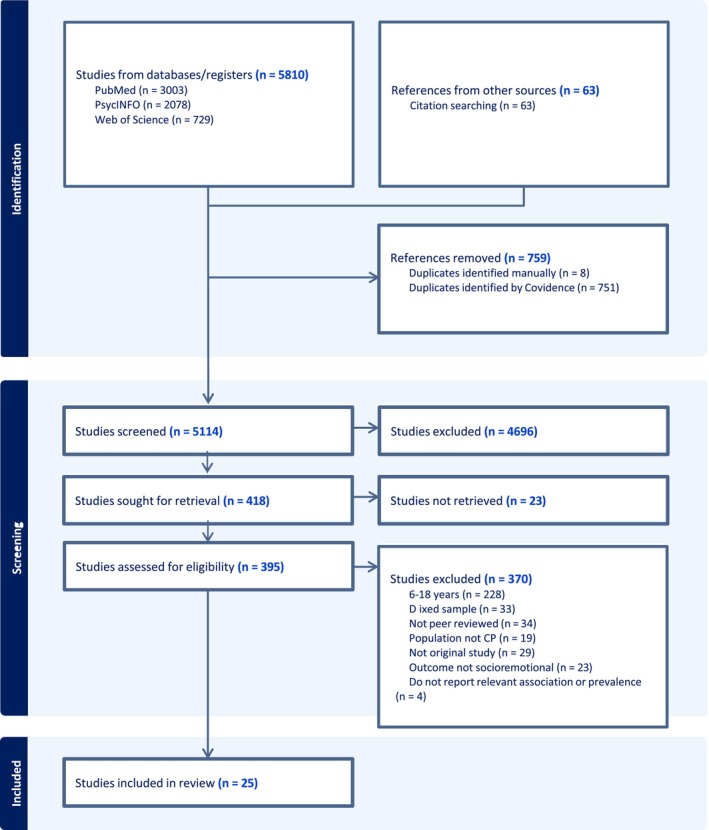
PRISMA (Preferred Reporting Items for Systematic Reviews and Meta‐Analyses) flow diagram (Tricco et al. 2018) of the study selection process.

All identified studies were collected and uploaded into Covidence systematic review software (Veritas Health Innovation, 2023, Melbourne, Australia), and duplicates were removed. Three reviewers (JEH + LLS + KR) independently screened titles and abstracts against the inclusion criteria of the review. Potentially relevant sources were retrieved in full text and assessed in detail independently by two reviewers (JEH + KR). Any disagreements were resolved by consensus. Reasons for exclusion of papers at the full text level were recorded (see Supporting Information S3 for details). The results of the search and the study selection process are presented in Figure 1.

### 2.4 Data Extraction

A data‐extraction form was developed and piloted prior to data collection. Data were extracted from papers included in the scoping review by two independent reviewers (JEH + KR) following guidelines for scoping reviews (Tricco et al. 2018). Any disagreements between reviewers were resolved through consensus.

The data extraction form reports on (1) study characteristics (e.g., authors, year of publication and country), (2) participant characteristics (e.g., sample size, age, gender and severity of motor disability), (3) methodology (e.g., study design, control group, measure name, measure type and version and subscale) and (4) reported findings (e.g., hypotheses presented, prevalence and factors investigated including their statistical significance).

A conceptual analysis of the included studies was conducted, inspired by the procedure described by Chagas et al. (2023). As such, all outcome measures and their operationalizations of socio‐emotional development were listed, as described in the measures section of the papers included. If a paper did not provide a specific description of how socio‐emotional development was operationalized, the cited paper detailing the specific method was identified, and the original description of the measure was recorded. The operationalizations were then classified according to the previously presented definition of socio‐emotional development, encompassing the following elements: (1) Experience, regulate and express *negative* emotions, (2) experience, regulate and express *positive* emotions, (3) forming close relationships with parents (including attachment), (4) forming close relationships with peers and (5) engaging in exploratory behaviour (including play). Measures could address multiple elements of this definition. All coding was performed independently by two authors (KR + JE). In case of disagreement, a third author (MSV) was consulted to resolve conflicts and reach consensus, but this was not necessary.

## 3 Results

The search yielded a total of 5810 papers, and the citation search identified an additional 63 papers. Following the removal of duplicates, 5114 records were screened at the title and abstract level. Of those papers, 395 full‐text papers were assessed for eligibility, and 370 papers were excluded. In total, 25 articles were included in the review (see Tables 1 and 2).

**TABLE 1:** Summary of the included studies describing study, participants, and methodological characteristics.

	Study and participant characteristics		Study methodology		
	Authors, country, publication year	Sample size *n*, Mean age (SD) [age range] Girls *n* (%)	Study design	Name of measure; measure type, version	Subscale used
Toddlers (1–3 years)	Banham (1973) US 1973	*n* = 37 n/a mean age [1.5–5 years] n/a girls	Cross‐sectional	n/a (study specific) observation	Social and emotional adjustment
	Brooks‐Gunn and Lewis (1984) US 1984	*n* = 34 n/a mean age [8–36 months] 15 (44.1) girls	Cross‐sectional	n/a observation	Behaviour
	Burgess et al. (2023) Australia 2022	*n* = 74 2:2 (0:1) ‐ 5:2 (0:2) years [2–5 years] 14 (36) girls	Longitudinal cohort	PEDI, Questionnaire Parent	Social function
	Eisenhower et al. (2005) US 2005	*n* = 10 34.7 (2.4) months [30–40 months] 6 (60) girls	Longitudinal cohort	CBCL, Questionnaire Parent	Internalizing, externalizing and total problem behaviours
	Fuertes and dos Santos (2003) Portugal 2003	*n* = 19 25.3 (4.3) months [18–32 months] 8 (42) girls	Cross‐sectional	SSP, Observation, manual coding	Attachment
	Konst et al. (2014) US 2014	*n* = 89 27.1 (5) months [17–35 months] 36 (40.4) girls	Cross‐sectional	BISCUIT‐Part 1, Interview Parent	Socialization/nonverbal communication
	Lipscombe et al. (2016) Australia 2016	*n* = 71 Time 1: 2 (n/a) years [n/a] 28 (39.4) girls	Prospective cohort	PEDI, Questionnaire Parent	Social function
	Marvin and Pianta (1996) US 1996	*n* = 70 Median: 34 months [1.2–4.5 years] 26 (37.1) girls	Cross‐sectional	SSP, Observation, manual coding	Attachment
	Sharawat and Panda (2022) India 2022	*n* = 103 2.6 (0.9) years [1–4 years] 29 (28.2) girls	Cross‐sectional	ITQOL, Questionnaire Parent	Temperament/moods, general behaviour, getting along
	Sipes et al. (2011) US 2011	*n* = 15 28.3 (5.29) months [20–35 months] 6 (40) girls	Cross‐sectional	BISCUIT‐part 3, Interview Parent	Aggressive/Destructive behaviours, Stereotypic Behaviours, Self‐Injurious Behaviours and total Behaviours.
	Tan et al. (2014) Netherlands 2014	*n* = 97 n/a mean age [1–4 years] 43 (44.0) girls	Longitudinal cohort	TNO‐AZL, Questionnaire Parent	Psychological functioning & social functioning
	Whittingham et al. (2010) Australia 2010	*n* = 122 1.5 (n/a) years [1–3 years] 42 (34.4) girls	Prospective cohort	PEDI, Questionnaire Parent	Social function
	Wu et al. (2021) China 2021	*n* = 300 n/a mean age [1–3 years] 108 (36.0) girls	Cross‐sectional	CITSEA Questionnaire Parent	Externalizing behaviour, Internalizing behaviour, dysregulation, and competence
Early childhood (4–5 years)	Alsem et al. (2013) Netherlands 2013	*n* = 72 n/a mean age [2–6 years] 31 (43.1) girls	Longitudinal cohort	TNO‐AZL, Questionnaire Parent	Positive mood, Behaviour problems, anxiety, social functioning
	Chang et al. (2012) Taiwan 2012	*n* = 47 3.8 (n/a) years [2–6 years] 17 (36.2) girls	Cross‐sectional	PedsQL 4.0, Questionnaire Parent	Psychosocial health
	Chen et al. (2011) Taiwan 2011	*n* = 105 4.8 (1.8) years [3–6 years] 38 (36.2) girls	Cross‐sectional	CCDI, Questionnaire Parent	Personal social
	Chen et al. (2013) Taiwan 2013	*n* = 78 3.7 (1.6) years [1–5 years] 29 (37.2) girls	Prospective cohort	CDIIT, Interview Parent	Social skills
	Gehrmann et al. (2014) Australia 2014	*n* = 151 53.7 (6.3) months [45–65 months] 64 (42.4) girls	Cross‐sectional	PEDI, Questionnaire Parent	Social function
	Horwood et al. (2019) Canada 2019	*n* = 34 n/a mean age [4–5 years] n/a girls	Cross‐sectional	SDQ, Questionnaire Parent	Emotional symptoms, conduct problems, hyperactivity, peer problems, total difficulties
	Keawutan et al. (2018) Australia 2018	*n* = 58 5 (n/a) years [5 years] 19 (32.8) girls	Cross‐sectional	CP QOL‐Child, Questionnaire Parent	Social well‐being and acceptance, emotional well‐being and self‐esteem
	Lai et al. (2017) Taiwan 2017	*n* = 78 37.0 (14.6) months [1–5 years] 29 (37.2) girls	Prospective cohort	TNO‐AZL, Questionnaire Parent	Social functioning & emotional functioning
	Moraleda‐Barreno et al. (2011) Spain 2011	*n* = 50 n/a (n/a) [0–5 years] 25 (50%) girls	Cross‐sectional	BDI, Interview, questionnaire and observation Parent & investigator	Personal‐social
	Romeo et al. (2014) Italy 2014	*n* = 100 3.8 (0.8) years [3–5 years] 48 (48) girls	Cross‐sectional	CBCL, Questionnaire Parent	Internalizing, externalizing and total problem behaviours
	Sigurdardottir et al. (2010) Iceland 2010	*n* = 36 4.9 (0.4) years [4–5 years] 17 (47) girls	Cross‐sectional	CBCL, Questionnaire Parent &Teacher	Internalizing, externalizing and total problem behaviours
	Wanamaker and Glenwick (1998) US 1998	*n* = 64 4.8 (1.2) years [3–6 years] 29 (45.3) girls	Cross‐sectional	ECBI, Questionnaire Parent	Intensity of problem behaviour

Abbreviations: BDI = Battelle Developmental Inventory Screening Test; BISCUIT = Baby and Infant Screen for Children With Autism Traits; CBCL = Child Behavior Checklist; CDIIT = Comprehensive Developmental Inventory for Infants and Toddlers; CCDI = Chinese Child Development Inventory; CITSEA = the standardized Chinese Version of Infant Toddler Social–Emotional Assessment; CP QOL‐Child = cerebral palsy quality of life questionnaire for children; ECBI = the Eyberg Child Behavior Inventory; ITQOL = Infant Toddler Quality of Life; n/a = not available; PEDI = Pediatric Evaluation of Disability; PedsQL = the Pediatric Quality of Life Inventory Version 4.0 for Toddlers (ages 2–4) and young children (ages 5–7); SDQ = Strengths and Difficulties Questionnaire; SSP = Strange Situation Procedure; TNO‐AZL  = Preschool Children Quality of Life.

**TABLE 2:** Summary of the included studies describing findings reported.

	Findings reported		
	Authors, country, publication year	Prevalence	Factors investigated
Toddlers (1–3 years)	Banham (1973) US 1973	n/a	General cognitive impairment (IQ etc.)*
	Brooks‐Gunn and Lewis (1984) US 1984	Significantly lower frequency of infant behaviour among children with CP vs. infants with developmental delay and infants with Downs Syndrome effect, *F*(2,91) = 5.26, *p* < 0.01	n/a
	Burgess et al. (2023) Australia 2022	Mean scaled scores (SD). Baseline (2 years): CP: 54.3 (5.2), norm: 58.2 (4.4). Follow‐up (5 years): CP: 63.2 (7.7), norm: 65.5 (4.5).	Communication*
	Eisenhower et al. (2005) US 2005	Percentage of children in borderline clinical range at age 3 years old: CP = 50%, typically development = 10.3%, undifferentiated developmental delays = 41.5%, Downs syndrome = 8.3%, autism = 46.2%.	Mothers´ level of education
	Fuertes and dos Santos (2003) Portugal 2003	Secure attachment: 9, avoidant: 5, ambivalent: 4	Parenting (Parent caregiver involvement)
	Konst et al. (2014) US 2014	Mean scores (SD). Children with CP 9.28, (11.24) vs. children with CP and ASD 30.11 (11.89) vs. children with Downs Syndrome 5.55 (7.63).	n/a
	Lipscombe et al. (2016) Australia 2016	PEDI social functioning at 60 months: 47.9% > 2 SD below the mean	GMFCS level*, Communication*
	Marvin and Pianta (1996) US 1996	51% insecurely attached (anxious or avoidant)	GMFCS level, General cognitive impairment (IQ etc.), severity of disability, Parent reaction to diagnosis*, Time since diagnosis
	Sharawat and Panda (2022) India 2022	n/a	Type of CP, GMFCS*, Epilepsy/seizures, General cognitive impairment (IQ etc.), Communication*, manual ability, eating and drinking ability, vision, white vs grey matter injury, maldevelopments, protein energy malnourishments, maternal education, paternal education, SES, residence, no. of siblings
	Sipes et al. (2011) US 2011	No significant differences were found between CP, Developmental Delay, Prematurity and Seizures for BISCUIT total scores (*p* = 0.56) or any of the subscales (*p* = 0.40).	Type of CP, sleep*,
	Tan et al. (2014) Netherlands 2014	Psychological function (toddlers) Mean CP vs. reference: 83.5 (9.3) vs. 85.7 (8.1), *p* = 0.028 Social function: 83.5 (21.0) vs. 91.3 (15.4), *p* = 0.000	Type of CP*, GMFCS*, General cognitive impairment (IQ etc.) *
	Whittingham et al. (2010) Australia 2010	Frequency of children (%) > 2 SD and > 1 SD below the mean. 18 months: 27 (44.3%), 17 (27.9%). 30 months: 41 (50.6%), 14 (17.3%).	GMFCS*
	Wu et al. (2021) China 2021	Over cut‐off: Externalizing behaviour (16%), internalizing behaviour (8%), dysregulation domain (10.7%), competence domain (13.3%).	Type of CP*, gestational age*, asphyxia*, abnormal EEG, delivery mode*, birth weight*, parental mental health*, parenting style*, primary caregiver*, attending recreation activities*, siblings, time since final diagnosis*
Early childhood (4–5 years)	Alsem et al. (2013) Netherlands 2013	n/a	Type of CP*, GMFCS* (longitudinal)
	Chang et al. (2012) Taiwan 2012	n/a	Type of CP, General cognitive impairment (IQ etc.), Communication*, Drooling, gross motor function
	Chen et al. (2011) Taiwan 2011	n/a	GMFCS*, temperament patterns*, attention span*
	Chen et al. (2013) Taiwan 2013	n/a	Type of CP, GMFCS*, Selective motor control, spinal alignment, muscle spasticity
	Gehrmann et al. (2014) Australia 2014	Children with CP ≥ 1 SD below norm (%): 104 (68.9). 23% of children with CP scored ≥ 4 SD below norm	GMFCS*, prematurity, SES
	Horwood et al. (2019) Canada 2019	Total difficulties CP vs. norms: Borderline (19.5%) vs. (10%), high (10.6%) vs. (5%), very high (15.0%) vs. (5%)	n/a
	Keawutan et al. (2018) Australia 2018	n/a	GMFCS*, Physical activity
	Lai et al. (2017) Taiwan 2017	n/a	GMFCS*
	Moraleda‐Barreno et al. (2011) Spain 2011	Children with CP experience significantly more personal/social problems than the control group, *p* < 0.001	Type of CP
	Romeo et al. (2014) Italy 2014	Percentage of pathological scores among children with CP: Total = 14%, internalizing = 13%, externalizing = 15%.	Type of CP, sleep*
	Sigurdardottir et al. (2010) Iceland 2010	High CBCL scores (> 84th percentile) CP vs. Comparison. Total problems: 48 vs. 18% High C‐TRF scores (> 84th percentile) CP vs. Comparison. Total problems: 65 vs. 18%	GMFCS*, General cognitive impairment (IQ etc.)*
	Wanamaker and Glenwick (1998) US 1998	n/a	Parental stress, Mental health (depression, anxiety etc.), Parenting* (perception of child, parenting style), social support

*Note:* GMFCS = Gross Motor Function Classification System; n/a = not available.

* Statistically significant risk/protective factor.

### 3.1 Study Characteristics

The 25 included studies were conducted between 1973 and 2022, with the majority conducted after 2010 (*n* = 19; 76%). Most of the included studies were conducted in Western countries, with nine studies from North America (Canada, United States), five from Oceania (Australia) and six from Europe (Iceland, Italy, Netherlands, Portugal, Spain) (*n* = 19; 76%). Six studies were conducted in Asian countries, including Taiwan (*n* = 4), China (*n* = 1) and India (*n* = 1). A list of sources for funding for the included studies can be seen in Supporting Information S4.

### 3.2 Participants Characteristics

The 25 studies included in the review represent data from 1914 children. All children were diagnosed with CP, and none were diagnosed as ‘high risk of CP’. Sample sizes in the individual studies ranged from 10 (Eisenhower et al. 2005) to 300 children (Wu et al. 2021) with a mean sample size of 76.6 children.

**FIGURE 2:**
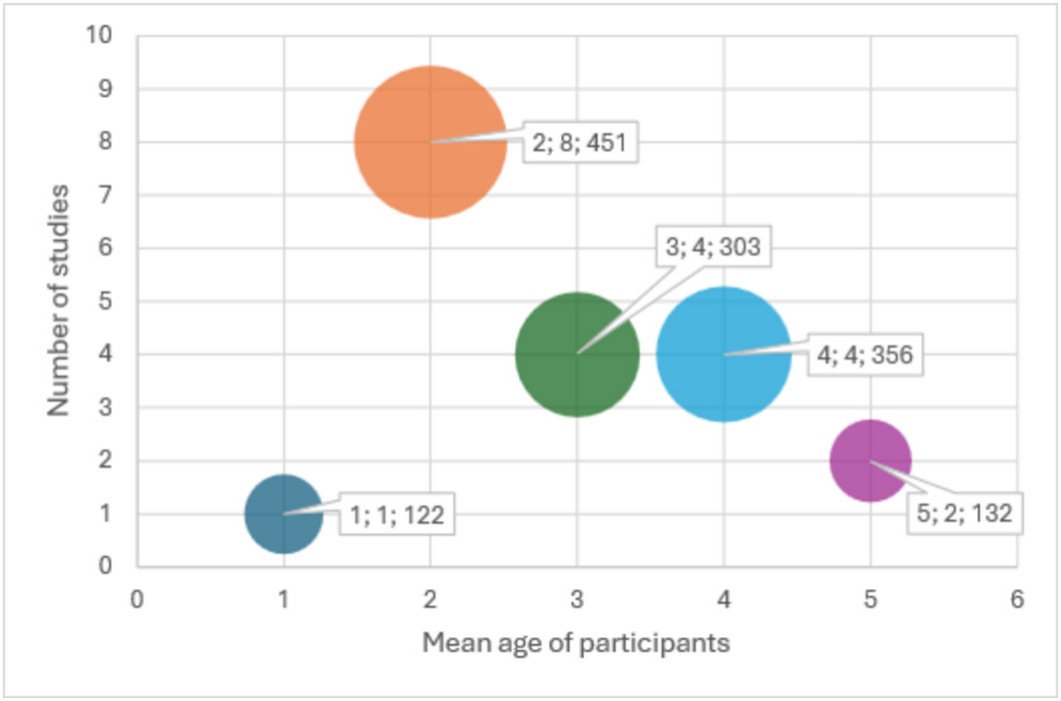
Bubble plot illustrating the distribution of studies and participants by child mean age. *Note:* Bubbles are arranged according to the reported mean age of participants across studies. The *x*‐axis represents the mean age (e.g., 1 = studies with a mean participant age of 1 year); the *y*‐axis shows the number of studies reporting data for each mean age group. The size of each bubble reflects the total number of participants included in studies at that age. Seven studies did not report a mean age. Numbers in the boxes indicate: Mean age; number of studies; total number of participants.

Mean age of the participants in the studies varied between 18 months (Whittingham et al. 2010) to 5 years (Burgess et al. 2023; Keawutan et al. 2018). A bubble plot illustrates the distribution of studies by mean participant age (see Figure 2). Children with a mean age of 2 were the most studied group of children. Only two studies included children younger than 1 year (Brooks‐Gunn and Lewis 1984; Moraleda‐Barreno et al. 2011), and none focused exclusively on infants. The size of each bubble reflects the number of participants at each mean age. Almost all studies that reported gender distribution included fewer females than males (21 of 23 studies) with a range from 28% to 48% females.

The Gross Motor Function Classification System (GMFCS) levels (level of motor difficulties) (Palisano et al. 1997) were reported (for the relevant age groups) in 13 of the 25 studies (52%). Two of the studies grouped children in GMFCS I–II, III–IV and V (Lai et al. 2017; Sigurdardottir et al. 2010). In the remaining 11 studies (representing 995 children), the total distribution of GMFCS levels were as follows: I (*n* = 361, 36.3%), II (*n* = 163, 16.4%), III (*n* = 168, 16.9%), IV (*n* = 153, 15.4%) and V (*n* = 150, 15.1%).

### 3.3 Study Methodology

Most studies were cross‐sectional (*n* = 17; 68%). Other studies were longitudinal and either prospective follow‐up studies (*n* = 4; 16%) (Chen et al. 2013; Lai et al. 2017; Lipscombe et al. 2016; Whittingham et al. 2010) or cohort studies (*n* = 4; 16%) (Alsem et al. 2013; Burgess et al. 2023; Eisenhower et al. 2005; Tan et al. 2014). All longitudinal studies were conducted in 2005 or later.

Five studies included a comparison group of typically developing children (*n* = 5; 20%) (Banham 1973; Chen et al. 2011; Moraleda‐Barreno et al. 2011; Romeo et al. 2014; Sigurdardottir et al. 2010), five studies compared the CP group to reference (norm) data from typically developing children (*n* = 5; 20%) (Burgess et al. 2023; Gehrmann et al. 2014; Horwood et al. 2019; Lipscombe et al. 2016; Tan et al. 2014) and three studies included other clinical groups (e.g., Downs syndrome, autism and undifferentiated developmental delay) as a comparison group (*n* = 3; 12%) (Brooks‐Gunn and Lewis 1984; Konst et al. 2014; Sipes et al. 2011). One study included both typically developing children and several clinical groups (*n* = 1; 4%) (Eisenhower et al. 2005). However, a substantial part of the studies did neither include a comparison group nor a comparison with norms (*n* = 11; 44%).

### 3.4 Measure Characteristics

A variety of measures were used. The Pediatric Evaluation of Disability Inventory (PEDI) (Haley et al. 1992) was used in four studies (16%), the Child Behavior Checklist (CBCL) (Achenbach and Rescorla 2001) in three studies (12%) and the Dutch version of the Preschool Child Quality of Life questionnaire (TNO‐AZL‐PF; Fekkes et al. 2000) also used in three studies. In most of the studies, the main outcomes were assessed via questionnaires (*n* = 17; 68%), with few studies using observations (such as the Strange Situation Procedure (SSP) (Ainsworth et al. 1978) or study‐specific observations) (*n* = 4; 16%) (Banham 1973; Brooks‐Gunn and Lewis 1984; Fuertes and dos Santos 2003; Marvin and Pianta 1996), clinical interviews (*n* = 3; 12%) (Chen et al. 2013; Konst et al. 2014; Sipes et al. 2011) and one study using a mix of clinical interview, questionnaire and observation (*n =* 1; 4%) (Moraleda‐Barreno et al. 2011). The majority of the questionnaires were parent reported (*n* = 16; %), with only one study including both parent and teacher reporting (Sigurdardottir et al. 2010), and another study using a mix of parents and researchers (Moraleda‐Barreno et al. 2011).

### 3.5 Conceptual Characteristics

**FIGURE 3:**
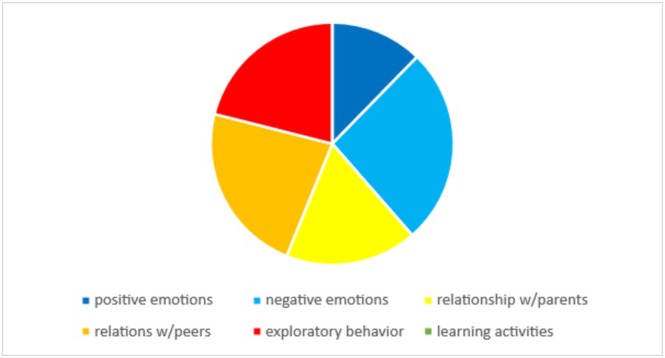
Conceptual analysis of socio‐emotional development utilized in included studies.

Results of the conceptual analysis are presented in Figure 3. All aspects of socio‐emotional development were examined. Experience, regulation and expression of *negative* emotions (*n* = 15; 26%), forming close relationships with peers (*n* = 13; 23%) and exploratory behaviour/play (*n* = 12; 21%) were the most investigated areas of socio‐emotional development compared with forming close relationships with parents (*n* = 10; 18%) and experience, regulation and expression of *positive* emotions (*n* = 7; 12%).

### 3.6 Reported Findings

Only nine studies (36%) made hypotheses regarding their expected findings. A little more than half of the studies (*n* = 14; 56%) reported on the prevalence of socio‐emotional problems based on norms for typically developing children or compared children diagnosed with CP to either typically developing or clinical groups of children. Reported prevalence of total adverse socio‐emotional development varied from 14% (Romeo et al. 2014) to 65% (Sigurdardottir et al. 2010). Different cut‐offs for the same measure were used in different studies. For example, when using the CBCL, two studies used a cut‐off score > 84^th^ percentile for socio‐emotional problems (Eisenhower et al. 2005; Sigurdardottir et al. 2010), while another study reported prevalence with a *t*‐score > 63 (app. 90^th^ percentile) (Romeo et al. 2014). Overall, the studies that compared children with CP to typically developing children (comparison group or norms) reported increased prevalences of socio‐emotional difficulties among children with CP (Burgess et al. 2023; Eisenhower et al. 2005; Gehrmann et al. 2014; Horwood et al. 2019; Lipscombe et al. 2016; Moraleda‐Barreno et al. 2011; Sigurdardottir et al. 2010; Tan et al. 2014; Whittingham et al. 2010). Studies comparing children diagnosed with CP to other clinical groups found that children with CP had more socio‐emotional difficulties compared with children with Down syndrome (Brooks‐Gunn and Lewis 1984; Eisenhower et al. 2005; Konst et al. 2014) but not to children with developmental delay (Eisenhower et al. 2005; Sipes et al. 2011).

**FIGURE 4:**
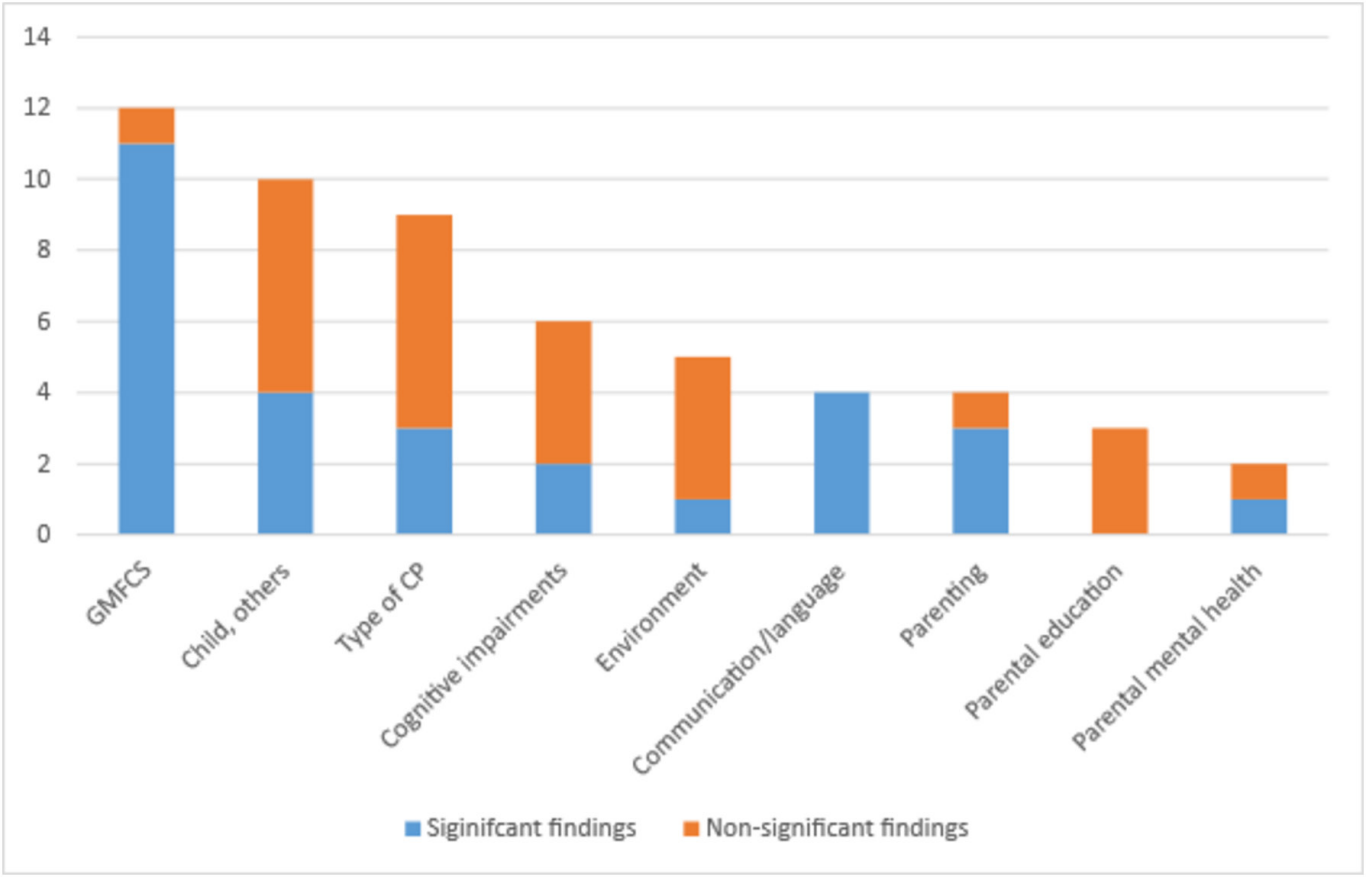
Factors investigated in included studies.

Predictors for socio‐emotional development were classified as either child or family related. Findings are presented in Figure 4. Most of the included studies investigated child‐related factors (*n* = 18; 72%) in relation to CP. The most common child‐related factors investigated were GMFCS level (*n* = 12; 48%), type of CP (*n* = 9; 36%), cognitive impairments (*n* = 6; 24%) and communication/language delay (*n* = 4; 16%). Seven studies (28%) examined family‐related factors in relation to child socio‐emotional development. The most investigated family‐related factor was parenting (caregiver involvement, parent reaction to diagnosis, parenting style, perception of child) (*n* = 4; 16%). Other investigated family‐related factors included parental education level, parental mental health, time since diagnosis, socio‐economic status and number of siblings (all *n* = 2; 8%). Severity of motor disability (GMFCS), delayed communication/language development, and parenting difficulties were found in most studies to be significantly associated with adverse socio‐emotional development in children diagnosed with or at risk for CP.

## 4 Discussion

This scoping review aimed to review the literature on socio‐emotional development in young children diagnosed with or at high risk for CP, focusing on conceptual and methodological issues to point to knowledge gaps and areas for future research.

Many studies investigated and reported adverse or atypical socio‐emotional development among young children with CP compared with typically developing peers. Half of the included studies reported a prevalence of nonoptimal socio‐emotional development ranging from 14% to 65%. Although estimates varied widely, this range is consistent with previous findings among school‐aged children and adolescents with CP, where approximately one‐third experience mental health difficulties (Downs et al. 2018). Several factors may explain the variability in the estimates. First, participant heterogeneity, such as differences in the distribution of GMFCS levels across samples, could influence prevalence rates (Reid et al. 2011). As higher GMFCS levels have been identified as a risk factor in some studies, a greater proportion of children with higher GMFCS levels might lead to higher prevalence rates. Second, small sample sizes (< 40 participants) in some studies may limit generalizability; future meta‐analyses could help address this limitation. Third, differences in instruments and cut‐off scores (e.g., CBCL) further complicate comparisons. Standardizing measures and thresholds would improve the comparability and validity of prevalence estimates. Overall, sample heterogeneity and methodological differences limit direct comparisons and complicate conclusions about the true prevalence of socio‐emotional difficulties among young children with CP. Future research should focus on addressing these inconsistencies to enhance reliability and generalizability.

Almost all the included studies (88%) investigated one or several factors in relation to socio‐emotional development, with three out of four studies investigating child‐related factors. Less studied were family‐related factors (28%). Across studies, severity of motor disability (GMFCS), delayed or atypical language/communicative development and parenting difficulties were reported as significant risk factors for nonoptimal socio‐emotional development. However, as the studies often include the child and family factors without a priori hypotheses regarding their expected impact on child socio‐emotional development, the results should be interpreted as exploratory and only used to generate hypotheses in future studies. We suggest including the three factors (severity of motor disability, language/communication and parenting difficulties) in future studies to examine their relationship and potential independent contribution to young children's socio‐emotional development. An example of this is one of the included studies (Lipscombe et al. 2016) that examined and found that early communicative abilities mediated the association between early motor abilities and later social functioning. As most of the included studies were cross‐sectional, inference about causal relationships is difficult. We suggest future longitudinal study designs to explore developmental trajectories over time.

Conceptually, the included studies focused on the child's abilities to experience, regulate and express negative emotions, as well as its engagement in peer relationships and exploratory behaviour/play. In contrast, positive emotions and parent–child relationships received less attention, highlighting a gap in the literature. Future studies should study these areas of socio‐emotional development.

Methodologically, the majority of the studies used questionnaires to investigate socio‐emotional development. Some of the questionnaires have been developed and validated to be used in a disability population, including those with CP (e.g., PEDI and CP QOL‐Child) (Haley et al. 1991; Waters et al. 2007) and aim to be inclusive of children using nonverbal communication methods (Burgess et al. 2023; Haley et al. 1991). However, other questionnaires (e.g., CBCL and SDQ) were originally developed and validated for use in the general population rather than in children with disabilities (Achenbach and Rescorla 2001; Goodman 1997). Certain items related to behaviour may be interpreted differently in a disability context, potentially affecting the accuracy of assessments (Downs et al. 2018). This issue is particularly relevant in the CP population, where cognitive and communicative impairments are common. When socio‐emotional development is assessed in children with cognitive impairments, overlapping symptoms can make it challenging to distinguish between socio‐emotional difficulties and the effects of cognitive impairments themselves (Einfeld et al. 2011), such as attention difficulties or social withdrawal. Moreover, few tools have been specifically designed for identifying socio‐emotional or mental health difficulties in young children with such impairments (Halvorsen et al. 2023). A recent systematic review recommended using instruments tailored to children and adolescents with intellectual impairments, as these demonstrated superior psychometric properties compared with those designed for the general population. However, among general population tools, the CBCL was identified as a relatively appropriate option for children and adolescents with intellectual impairments (Halvorsen et al. 2023). To ensure accurate assessment in the CP population, future research should prioritize validating socio‐emotional assessment tools tailored to the needs and competencies of these children.

Furthermore, most studies relied on parent‐ or teacher‐reported data through questionnaire studies on children's socio‐emotional functioning. Only four studies used structured observational methods, all conducted over 20 years ago. Adult‐reported assessments are vulnerable to informant bias, as adults may lack full insight into children's internal experiences (Cummins 2002) or interpret behaviours through the lens of their relationship with the child or their own mental health. For example, a recent study in typically developing children found that interparental disagreement about infants' socio‐emotional development was predicted by an interaction between maternal and paternal stress (Egmose et al. 2024). Similarly, among children aged 4–12 years with CP, parental distress mediated the link between child disability and parent‐reported quality of life (Davis et al. 2012). Given that parents of children with CP often report high stress levels (Pousada et al. 2013), this may influence their perceptions of their child's socio‐emotional well‐being.

Observational and multi‐informant methodologies are often considered ‘gold standard’ in assessing young children, as they themselves are typically unable to engage in standard questionnaire studies (McConaughy and Whitcomb 2021), a limitation that is further accentuated among children with communication difficulties. A range of structured and naturalistic observational measures exist, including the Strange Situation Procedure (Ainsworth et al. 1978) and its modified versions—the Cassidy‐Marvin system (children aged 2.5–4.5 years), and the Main‐Cassidy system (children aged 6‐ and‐7‐year‐olds) (Deneault et al. 2023), as well as the Attachment Q‐sort (Waters and Deane 1985), which all assess the parent–child attachment relationship. Emotion regulation tasks, such as ‘do’ and ‘do not’ contexts (Kochanska et al. 2001), and structural observational tools for peer interactions, such as inCLASS (Downer et al. 2010), are also valid methods. Another promising approach could potentially be the ‘puppet interview technique’, which can be administrated to children as young as 4 years old, including nonverbal communicators, as a self‐report measure (Measelle and Ablow 2017). Studies employing puppet interviews have shown that young children are capable of providing valid information about their own socio‐emotional problems and well‐being (Ringoot et al. 2013) and, in a paediatric population, about own illnesses and QoL (Herzog et al. 2024). Future studies should incorporate observational methods, in conjunction with self‐ and multi‐informant assessment when appropriate.

Studies included both toddlers and preschool children with an overall mean age of 27 months. Only two studies included infants under the age of 1; however, the age range of these studies were broad with 0–5 years (Moraleda‐Barreno et al. 2011) and 8–36 months (Brooks‐Gunn and Lewis 1984). No studies focused exclusively on infants. CP has historically been diagnosed in the second year of life (Hoei‐Hansen et al. 2023; Novak et al. 2017), which explains the lack of studies including infants. However, recent clinical guidelines recommend giving an early diagnosis of ‘high risk of CP’ before 6 months of age when CP is suspected but cannot be confirmed, to ensure referral to CP‐specific rehabilitation (Morgan et al. 2021). Of particular interest for this review, these recommendations include supporting attachment formation between parents and their infants, despite limited knowledge about what characterizes parent–infant interactions during the early years or whether attachment formation is affected by the risk of CP. As relationship‐supporting interventions are often overlooked in current CP programmes (Morgan et al. 2021), raising awareness on how socio‐emotional development might be affected in infants with or at ‘high risk of CP’ is crucial and can underscore the critical need for such interventions.

The findings should be interpreted in the light of some geographical and cultural limitations, as the included studies were primarily conducted in Western high‐income countries, such as Australia, the United States, Canada and several European countries. Findings from these contexts may not be generalizable to countries with different cultural, social or economic conditions. The included studies from Asia (Taiwan, India and China, with varying income levels) often lacked a comparison group and did not report prevalence rates, limiting the conclusions that can be drawn about the socio‐emotional development of children with CP across diverse cultural contexts. However, similar to the studies from Western high‐income countries, most of the studies conducted in Asia identified an association between severity of motor disability (GMFCS level) and adverse socio‐emotional development. While most existing studies originate from Western high‐income countries and a few Asian contexts, other parts of the world—such as Africa, Latin America and the Middle East—remain largely unexplored. Expanding research to these regions is essential to ensure culturally and globally applicable knowledge. Moreover, economic development, cultural practices and access to services may differ significantly between countries within regions, further complicating cross‐cultural comparisons.

## 5 Limitations

This scoping review has some limitations. Despite search efforts, there were some studies that we were not able to locate, mainly older studies, due to problems with accessing nondigitized publications. Further, we made some changes from the prepublished protocol. However, it is within the framework of scoping reviews, due to its exploratory nature, to gain insight from the preliminary searches and thereby modify aims or inclusion criteria (Arksey and O'Malley 2005). To ensure transparency, any changes made from the protocol have been recorded and can be seen in the OSF registration.

## 6 Conclusion

In conclusion, existing studies of socio‐emotional development in young children diagnosed with CP are characterized by the use of parent‐reported measures about children's socio‐emotional development, cross‐sectional designs and a conceptual focus on negative emotionality and relationships with peers, including exploratory behaviours. Despite methodological and conceptual heterogeneity, which limits direct comparison of study findings, socio‐emotional difficulties are commonly reported. Further, the severity of motor disability, language/communicative impairments and parenting difficulties are consistently found to be significant risk factors for nonoptimal socio‐emotional development. Taken together, this scoping review confirms that more knowledge is needed to understand the factors that affect socio‐emotional development in young children diagnosed with or at high risk for CP. Future studies should include observational and self‐ or multi‐informant assessment, ideally in longitudinal designs starting in infancy and including geographically and culturally diverse populations.

## Author Contributions

The first author has contributed to research design, analysis and interpretation of data, drafting and revising the paper critically and approval of the submitted and final version. The second author has contributed to analysis of data, revising the paper critically and approval of the submitted and final version. The third author has contributed to research design, revising the paper critically and approval of the submitted and final version. The fourth and last author has contributed to research design, analysis and interpretation of data, drafting and revising the paper critically and approval of the submitted and final version.

## Ethics Statement

The authors have nothing to report.

## Conflicts of Interest

The authors declare no conflicts of interest.

## Supporting information


**Data S1** Preferred Reporting Items for Systematic reviews and Meta‐Analyses extension for Scoping Reviews (PRISMA‐ScR) Checklist.

**Data S2** Full Search String.

**Data S3** Studies excluded at full‐text level with reasons for exclusion.

**Data S4** Sources of funding and conflict of interest of the included studies.

## Data Availability

This study is a scoping review of existing literature. All the data that were used in this review were obtained from publicly available sources, as cited in the manuscript.
