# Supplementary material for: Socio‐Emotional Development in Young Children With Cerebral Palsy: A Scoping Review
Source: Child Care Health Dev. 2025 Jul 2;51(4):e70130. doi: 10.1111/cch.70130 (PMC12223170; doi:10.1111/cch.70130)
Supplement: Supplementary file 2 — Data S2 Full Search String. [file CCH-51-e70130-s001.docx]

Supplementary material 2

*Full Search String*

| Database | Search string |
| --- | --- |
| *PsycInfo | ( Cerebral palsy OR CP ) AND  ( Child* OR infant* OR adolescen* OR toddler* OR preschool OR youth ) AND  ( emotion OR affect OR “emotion* develop*” OR “affect* regulat*” OR “emotion* regulat*” OR “emotion* symptom*” OR “emotion* problem* “OR “emotion* difficult*” OR attachment OR “emotion* competence” OR self-regulation OR “emotion* understand*” OR “emotion* expres*” OR “Social behavio#r*” OR “behavio#r develop*” OR “behavio#r* symptom*” OR “behavio#r* problem*” OR “social problem*” OR “socio-emotion* difficult*” OR “socio-emotion* problem*” OR “peer problem*” OR “peer difficult*” OR “social  interaction*” OR “social interaction* problem*” OR “social interaction* difficult*” OR “social impair*” OR “social outcome*” OR “socializ* skill*” OR socializ* OR “social competence” OR relationship* OR “Psychol* problems*” OR “psychol* difficult*” OR “psychol* symptom*” OR “psychosoc* devel*“ OR Withdrawn OR “agressiv* behavio#r*” OR depress* OR anxi* OR “external* problem*” OR “internal*  problem*” OR shyness OR “social isolation” OR “Mental health” OR “psychiatric disorder*” OR “psych* problem*” OR psychopathol* ) |
| *Psycinfo thesarus | (DE "Cerebral Palsy") AND  (DE "Childhood Development" OR DE "Infant Development" OR DE "Adolescent Development") AND  (DE "Socioemotional Functioning" OR DE "Attachment Behavior" OR DE "Attachment Theory" OR DE "Emotional Regulation" OR DE "Social Behavior" OR DE "Social Skills" OR DE "Psychosocial Development" OR DE "Antisocial Behavior" OR DE "Behavior Problems" OR DE "Externalizing Symptoms" OR DE "Internalizing Symptoms" OR DE "Emotional Adjustment" OR DE "Emotional and Behavioral Disorders" OR DE "Emotional Development" OR DE "Emotional Disturbances" OR DE "Mental Disorders" OR DE "Mental Health" OR DE "Child  Psychopathology" OR DE "Adolescent Psychopathology") |
| *PubMED Text word | ( Cerebral palsy OR CP ) AND  ( Child* OR infant* OR adolescen* OR toddler* OR preschool OR youth ) AND  ( emotion OR affect OR “emotion develop*” OR “emotional develop*” OR “affect regulat*” OR “affectiv regulat*” OR “emotion regulat*” OR “emotion symptom*” OR “emotional symptom*” OR “emotion problem* “OR “emotional problem* “ OR “emotion difficult*” OR “emotional difficult*” OR attachment OR “emotion competence” OR “emotional competence” OR self-regulation OR “emotion |

|  | understand*” OR “emotional understand*” OR “emotion expres*” OR “emotional expres*” OR “Social behavio*” OR “behavior develop*” OR “behavioral develop*” OR “behavioral symptom*” OR “behavior problem*” OR “behaviour develop*” OR “behavioural develop*” OR “behavioural symptom*” OR “behaviour problem*” OR “social problem*” OR “socioemotional difficult*” OR “socioemotional problem*”  OR “peer problem*” OR “peer difficult*” OR “social interaction*” OR “social problem*” OR “social difficult*” OR “social impair*” OR “social outcome*” OR socializ* OR “social competence” OR relationship* OR “Psychological problems*” OR “Psychologic problems*” OR “Psychic problems*” OR “psychological difficult*” OR “psychologic difficult*” OR “psychic difficult*” OR “psychological symptom*” OR “psychologic symptom*” OR “psychic symptom*” OR “psychosocial devel*“ OR Withdrawn OR “aggressive behavio*” OR “aggression behavio*” OR depress* OR anxi* OR “external problem*” OR “externalizing problem*” OR “internal problem*” OR “internalizing problem*” OR “external sympt*” OR “externalizing sympt*” OR “internal sympt*” OR “internalizing sympt*” OR shyness OR “social isolation” “Mental health” OR “psychiatric disorder*” OR psychopathol* ) |
| --- | --- |
| *PubMed Mesh | "Cerebral Palsy"[MeSH Terms] AND  ("Child"[MeSH Terms] OR "Adolescent"[MeSH Terms] OR "child, preschool"[MeSH Terms] OR "Infant"[MeSH Terms] OR "infant, premature"[MeSH Terms] OR "infant, newborn"[MeSH Terms]) AND  ("Psychosocial Functioning"[MeSH Terms] OR "Emotional Regulation"[MeSH Terms] OR "Behavioral Symptoms"[MeSH Terms] OR "Child Behavior"[MeSH Terms] OR "Child Behavior Disorders"[MeSH Terms] OR "Infant Behavior"[MeSH Terms] OR "Adolescent Behavior"[MeSH Terms] OR "Mental Disorders"[MeSH Terms] OR "Mental Health"[MeSH Terms] OR "Psychopathology"[MeSH Terms]) |
| *Web of science  Topic search | ( “Cerebral palsy” OR CP ) AND  ( Child* OR infant* OR adolescen* OR toddler* OR preschool OR youth ) AND  ( emotion OR affect OR “emotion* develop*” OR “affect* regulat*” OR “emotion* regulat*” OR “emotion* symptom*” OR “emotion* problem* “OR “emotion* difficult*” OR attachment OR “emotion* competence” OR self-regulation OR “emotion* understand*” OR “emotion* expres*” OR “Social behavio#r*” OR “behavio#r develop*” OR “behavio#r* symptom*” OR “behavio#r* problem*” OR “social problem*” OR “socio-emotion* difficult*” OR “socio-emotion* problem*” OR “peer problem*” OR “peer difficult*” OR “social  interaction*” OR “social interaction* problem*” OR “social interaction* difficult*” OR “social impair*” OR “social outcome*” OR “socializ* skill*” OR socializ* OR “social competence” OR relationship* OR “Psychol* problems*” OR “psychol* difficult*” OR “psychol* symptom*” OR “psychosoc* devel*“ OR Withdrawn OR “agressiv* behavio#r*” OR depress* OR anxi* OR “external* problem*” OR “internal*  problem*” OR shyness OR “social isolation” OR “Mental health” OR “psychiatric disorder*” OR “psych* problem*” OR psychopathol* ) |
