# Supplementary material for: Socio‐Emotional Development in Young Children With Cerebral Palsy: A Scoping Review
Source: Child Care Health Dev. 2025 Jul 2;51(4):e70130. doi: 10.1111/cch.70130 (PMC12223170; doi:10.1111/cch.70130)
Supplement: Supplementary file 3 — Data S3 Studies excluded at full‐text level with reasons for exclusion. [file CCH-51-e70130-s002.docx]

Supplementary material 3

*Studies excluded at full-text level with reasons for exclusion*

Adams, M. S., Khan, N. Z., Begum, S. A., Wirz, S. L., Hesketh, T., C Pring, T. R. (2012).

Feeding difficulties in children with cerebral palsy: low-cost caregiver training in Dhaka, Bangladesh. *Child Care Health Dev*, *38*(6), 878-888.

<https://doi.org/10.1111/j.1365-2214.2011.01327.x> Exclusion reason: 6-18 years Adamson, L. (2003). Self-Image, Adolescence, and Disability. *American Journal of*

*Occupational Therapy*, *57*(5), 578-581. <https://doi.org/10.5014/ajot.57.5.578>. Exclusion reason: 6-18 years

Adegboye, D., Sterr, A., Lin, J.-P., C Owen, T. J. (2017). Theory of mind, emotional and

social functioning, and motor severity in children and adolescents with dystonic cerebral palsy. *European journal of paediatric neurology*, *21*(3), 549-556. <https://doi.org/10.1016/j.ejpn.2017.01.013>. Exclusion reason: 6-18 years

Aebi, U. (1976). [Early treatment of cerebral movement disorders: findings among 50 school children]. *Helv Paediatr Acta*, *31*(4-5), 319-333. Exclusion reason: 6-18 years

Agarwal, P., C Lim, S. B. (2003). Long-term follow-up and outcome of extremely-low- birth-weight (ELBW) infants. *Ann Acad Med Singap*, *32*(3), 346-353. Exclusion reason: Not original study

Ahn, B., Joung, Y. S., Kwon, J. Y., Lee, D. I., Oh, S., Kim, B. U., Cha, J. Y., Kim, J. H., Lee, J.

Y., Shin, H. Y., C Seo, Y. S. (2021). Effects of equine-assisted activities on

attention and quality of life in children with cerebral palsy in a randomized trial: examining the comorbidity with attention-deficit/hyperactivity disorder. *BMC Pediatr*, *21*(1), 135. <https://doi.org/10.1186/s12887-021-02597-0> Exclusion

reason: 6-18 years

Aidar, F. J., Silva Júnior, W. M. d., Carneiro, A., Gama de Matos, D., Garrido, N. D., Raphael Fabrício de Souza, R. F. d., Aidar, L. Z., C Victor Machado Reis, V. M. (2016). Analise das atividades aquáticas em relação a saúde, aprendizagem e

função social em paralizados cerebrais = Analysis of aquatic activities in relation to health, learning and social function in cerebral paralyzed. *Motricidade*,

*12*(Suppl 2), 11-18.

[https://search.ebscohost.com/login.aspx?direct=trueCdb=psyhCAN=2017-](https://search.ebscohost.com/login.aspx?direct=true&db=psyh&AN=2017-22824-002&site=ehost-livefjaidar%40gmail.com)

[22824-002Csite=ehost-livefjaidar@gmail.com](https://search.ebscohost.com/login.aspx?direct=true&db=psyh&AN=2017-22824-002&site=ehost-livefjaidar%40gmail.com). Exclusion reason: Do not report relevant association or prevalence

Akçay, E., Tanır, Y., Teber, S. T., C Kılıç, B. G. (2021). Motor functions, quality of life and maternal anxiety and depression in children with cerebral palsy of different

intelligence levels. *Turk J Pediatr*, *c3*(5), 846-854.

<https://doi.org/10.24953/turkjped.2021.05.012>. Exclusion reason: 6-18 years Al-Dababneh, K. A., C Al-Zboon, E. K. (2018). Parents’ attitudes towards their children

with cerebral palsy. *Early Child Development and Care*, *188*(6), 731-747. <https://doi.org/10.1080/03004430.2016.1230737>. Exclusion reason: 6-18 years

Alcantara, C. A. R. (2014). *ECOLOGICAL AND A CONTEXTUAL INTERVENTIONS IN BEHAVIORAL REHABILITATION OF A CHILD WITH CEREBRAL PALSY.* Exclusion

reason: Not peer reviewed

Alghamdi, M. S., Chiarello, L. A., Palisano, R. J., C McCoy, S. W. (2017). Understanding participation of children with cerebral palsy in family and recreational activities.

*Research in Developmental Disabilities*, *cS*, 96-104.

<https://doi.org/10.1016/j.ridd.2017.07.006>. Exclusion reason: 6-18 years Allen, M. C., Cristofalo, E., C Kim, C. (2010). PRETERM BIRTH: TRANSITION TO

ADULTHOOD. *Developmental Disabilities Research Reviews*, *1c*(4), 323-335. <https://doi.org/10.1002/ddrr.128>. Exclusion reason: Population not CP

Amini, M., Saneii, S. H., C Pashmdarfard, M. (2018). Factors affecting social

participation of Iranian children with cerebral palsy. *Occupational Therapy In*

*Health Care*, *32*(3), 290-305. <https://doi.org/10.1080/07380577.2018.1497820>. Exclusion reason: 6-18 years

Angeli, J. M., Peck, M. N., C Schwab, S. M. (2019). Self-Perceived Scholastic

Competence, Athletic Competence, and Physical Appearance Are Enhanced in Children and Young Adults with Physical Disabilities Following a Community-

Based Running Program. *Journal of Developmental and Physical Disabilities*,

*31*(5), 707-723. <https://doi.org/10.1007/s10882-019-09690-4>. Exclusion reason:

6-18 years

Aran, A., Shalev, R. S., Biran, G., C Gross-Tsur, V. (2007). Parenting style impacts on

quality of life in children with cerebral palsy. *The Journal of Pediatrics*, *151*(1), 56-

60. <https://doi.org/10.1016/j.jpeds.2007.02.011>. Exclusion reason: 6-18 Arnaud, C., White-Koning, M., Michelsen, S. I., Parkes, J., Parkinson, K., Thyen, U.,

Beckung, E., Dickinson, H. O., Fauconnier, J., Marcelli, M., McManus, V., C

Colver, A. (2008). Parent-reported quality of life of children with cerebral palsy in Europe. *Pediatrics*, *121*(1), 54-64. <https://doi.org/10.1542/peds.2007-0854>.

Exclusion reason: 6-18 years

Asam, U. (1978). [Minimal cerebral dysfunction, minimal cerebral palsy. Concepts, pathology, psychosocial importance (author's transl)]. *Offentl Gesundheitswes*, *40*(10), 673-678. Exclusion reason: Not able to locate study

Asano, D., Takeda, M., Nobusako, S., C Morioka, S. (2020). Self-Rated Depressive Symptoms in Children and Youth with and without Cerebral Palsy: A Pilot Study. *BEHAVIORAL SCIENCES*, *10*(11). <https://doi.org/10.3390/bs10110167>. Exclusion reason: 6-18 years

Badia, M., Longo, E., Orgaz, M. B., C Gómez-Vela, M. (2013). The influence of participation in leisure activities on quality of life in spanish children and

adolescents with cerebral palsy. *Research in Developmental Disabilities*, *34*(9), 2864-2871. <https://doi.org/10.1016/j.ridd.2013.06.017>

Badia, M., Orgaz, M. B., Gómez-Vela, M., Verdugo, M. A., Ullán, A. M., C Longo, E. (2016).

Do environmental barriers affect the parent-reported quality of life of children and adolescents with cerebral palsy? *Research in Developmental Disabilities*,

*4S-50*, 312-321. <https://doi.org/10.1016/j.ridd.2015.12.011>. Exclusion reason: 6-

18 years

Badia, M., Riquelme, I., Orgaz, B., Acevedo, R., Longo, E., C Montoya, P. (2014). Pain,

motor function and health-related quality of life in children with cerebral palsy as reported by their physiotherapists. *BMC pediatrics*, *14*(1), 1-6. Exclusion reason: 6-18 years.

Bahl, A. B., C Freeman, K. A. (2019). Challenging behaviors, sleep and toileting. In (pp.

195-210). Mac Keith Press.

https://search.ebscohost.com/login.aspx?direct=trueCdb=psyhCAN=2018- 31846-012Csite=ehost-live. Exclusion reason: Not peer reviewed

Bakaeva, I. A., Novokhat'ko, E. N., C Shevyreva, E. G. (2019). Defense Activities and Coping Strategies at Children with Cerebral Palsy and their Parents. *SIBIRSKIY PSIKHOLOGICHESKIY ZHURNAL-SIBERIAN JOURNAL OF PSYCHOLOGY*(71),

180-196. <https://doi.org/10.17223/17267080/71/10>. Exclusion reason: 6-18 years Balboni, G., Rebecchini, G., Elisei, S., C Tassé, M. J. (2020). Factors affecting the

relationship between adaptive behavior and challenging behaviors in individuals with intellectual disability and co-occurring disorders. *Research in Developmental Disabilities*, *104*. <https://doi.org/10.1016/j.ridd.2020.103718>.

Exclusion reason: Population not CP

Banjanin, M., Slaviček, I., C Folnegović-Šmalc, V. (2002). Undesirable behavior in children with cerebral palsy in different accommodation and treatment

conditions. *Socijalna Psihijatrija*, *30*(3), 165-170.

[https://search.ebscohost.com/login.aspx?direct=trueCdb=psyhCAN=2002-](https://search.ebscohost.com/login.aspx?direct=true&db=psyh&AN=2002-18509-003&site=ehost-live) [18509-003Csite=ehost-live](https://search.ebscohost.com/login.aspx?direct=true&db=psyh&AN=2002-18509-003&site=ehost-live). Exclusion reason: 6-18 years.

Bantjes, J., Swartz, L., Conchar, L., C Derman, W. (2015). When they call me cripple: A group of South African adolescents with cerebral palsy attending a special needs school talk about being disabled. *Disability & Society*, *30*(2), 241-254.

<https://doi.org/10.1080/09687599.2014.997352>. Exclusion reason: 6-18 years Barfoot, J., Meredith, P., Ziviani, J., C Whittingham, K. (2015). Relationship-focused

parenting intervention to support developmental outcomes for a young child with cerebral palsy: A practice application. *The British Journal of Occupational*

*Therapy*, *78*(10), 640-643. <https://doi.org/10.1177/0308022615583304>. Exclusion

reason: 6-18 years

Barfoot, J., Meredith, P., Ziviani, J., C Whittingham, K. (2017). Parent‐child interactions and children with cerebral palsy: An exploratory study investigating emotional availability, functional ability, and parent distress. *Child: Care, Health and Development*, *43*(6), 812-822. <https://doi.org/10.1111/cch.12493>. Exclusion

reason: 6-18 years

Barnett, D., Hunt, K. H., Butler, C. M., McCaskill, J. W. I. V., Kaplan-Estrin, M., C Pipp- Siegel, S. (1999). Indices of attachment disorganization among toddlers with neurological and non-neurological problems. In (pp. 189-212). The Guilford Press.

[https://search.ebscohost.com/login.aspx?direct=trueCdb=psyhCAN=1999-](https://search.ebscohost.com/login.aspx?direct=true&db=psyh&AN=1999-04187-007&site=ehost-live) [04187-007Csite=ehost-live](https://search.ebscohost.com/login.aspx?direct=true&db=psyh&AN=1999-04187-007&site=ehost-live). Exclusion reason: Mixed sample

Barnett, D., Kaplan-Estrin, M., Braciszewski, J., Hetterscheidt, L., Issner, J., C Butler, C.

M. (2011). Maternal solicitousness and attachment disorganization among toddlers with a congenital anomaly. In (pp. 245-266). The Guilford Press. [https://search.ebscohost.com/login.aspx?direct=trueCdb=psyhCAN=2011-](https://search.ebscohost.com/login.aspx?direct=true&db=psyh&AN=2011-16269-009&site=ehost-live) [16269-009Csite=ehost-live](https://search.ebscohost.com/login.aspx?direct=true&db=psyh&AN=2011-16269-009&site=ehost-live). Exclusion reason: Mixed sample

Barrera, M. E., C Vella, D. M. (1987). Disabled and nondisabled infants' interactions with their mothers. *American Journal of Occupational Therapy*, *41*(3), 168-172. <https://doi.org/10.5014/ajot.41.3.168>. Exclusion reason: Mixed sample

Basil, C. (1992). Social interaction and learned helplessness in severely disabled children. *AAC: Augmentative and Alternative Communication*, *8*(3), 188-199. <https://doi.org/10.1080/07434619212331276183>. Exclusion reason: 6-18 years

Bates, L., Taylor, M., Lin, J. P., Gimeno, H., Kingston, J., C Rudebeck, S. R. (2021). Mental health and behaviour in children with dystonia: Anxiety, challenging behaviour

and the relationship to pain and self-esteem. *Eur J Paediatr Neurol*, *35*, 40-48. <https://doi.org/10.1016/j.ejpn.2021.09.002>. Exclusion reason: 6-18 years

Batshaw, M. L., Pellegrino, L., C Roizen, N. J. (2007). *Children with disabilities, cth ed*.

Paul H. Brookes Publishing Co.

[https://search.ebscohost.com/login.aspx?direct=trueCdb=psyhCAN=2007-](https://search.ebscohost.com/login.aspx?direct=true&db=psyh&AN=2007-03652-000&site=ehost-live) [03652-000Csite=ehost-live](https://search.ebscohost.com/login.aspx?direct=true&db=psyh&AN=2007-03652-000&site=ehost-live). Exclusion reason: Not original study

Battle, C. U. (1974). Disruptions in the socialization of a young, severely handicapped child. *Rehabilitation Literature*, *35*(5), 130-140.

[https://search.ebscohost.com/login.aspx?direct=trueCdb=psyhCAN=1974-](https://search.ebscohost.com/login.aspx?direct=true&db=psyh&AN=1974-27795-001&site=ehost-live) [27795-001Csite=ehost-live](https://search.ebscohost.com/login.aspx?direct=true&db=psyh&AN=1974-27795-001&site=ehost-live). Exclusion reason: Not able to locate study

Bean, J. (1995). Music therapy and the child with cerebral palsy: Directive and non- directive intervention. In (pp. 194-208). Harwood Academic Publishers/Gordon. [https://search.ebscohost.com/login.aspx?direct=trueCdb=psyhCAN=1995-](https://search.ebscohost.com/login.aspx?direct=true&db=psyh&AN=1995-98396-011&site=ehost-live)

[98396-011Csite=ehost-live](https://search.ebscohost.com/login.aspx?direct=true&db=psyh&AN=1995-98396-011&site=ehost-live). Exclusion reason: Not peer reviewed

Beckung, E., C Hagberg, G. (2002). Neuroimpairments, activity limitations, and participation restrictions in children with cerebral palsy. *Developmental Medicine & Child Neurology*, *44*(5), 309-316.

<https://doi.org/10.1017/S0012162201002134>. Exclusion reason: 6-18 years Beckung, E., White-Koning, M., Marcelli, M., McManus, V., Michelsen, S., Parkes, J.,

Parkinson, K., Thyen, U., Arnaud, C., Fauconnier, J., C Colver, A. (2008). Health status of children with cerebral palsy living in Europe: a multi-centre study.

*CHILD CARE HEALTH AND DEVELOPMENT*, *34*(6), 806-814.

<https://doi.org/10.1111/j.1365-2214.2008.00877.x>. Exclusion reason: 6-18 years Behle, A. E., C Pinquart, M. (2015). Perceived Attainment of Developmental Tasks in

Adolescents with and without Physical Disabilities. *Journal of Developmental and Physical Disabilities*, *27*(6), 773-787. [https://doi.org/10.1007/s10882-015-](https://doi.org/10.1007/s10882-015-9454-0)

[9454-0](https://doi.org/10.1007/s10882-015-9454-0). Exclusion reason: 6-18 years

Belmonte-Darraz, S., Montoro, C. I., Andrade, N. C., Montoya, P., C Riquelme, I. (2021). Alteration of emotion knowledge and its relationship with emotion regulation and psychopathological behavior in children with cerebral palsy. *Journal of Autism and Developmental Disorders*, *51*(4), 1238-1248.

<https://doi.org/10.1007/s10803-020-04605-1>. Exclusion reason: 6-18 years Belmonte, S., Montoya, P., González-Roldán, A. M., C Riquelme, I. (2019). Reduced brain

processing of affective pictures in children with cerebral palsy. *Research in Developmental Disabilities*, *S4*. <https://doi.org/10.1016/j.ridd.2019.103457>. Exclusion reason: 6-18 years

Benassi, B. J., C Benassi, V. A. (1973). Behavioral strategies for a deaf and cerebral palsied child. *Journal of Communication Disorders*, *c*(3), 165-174.

<https://doi.org/10.1016/0021-9924(73)90003-8>. Exclusion reason: Outcome not socioemotional

Berney, T. (2009). Management of emotional and behavioural problems. In (pp. 58-66).

Mac Keith Press.

[https://search.ebscohost.com/login.aspx?direct=trueCdb=psyhCAN=2009-](https://search.ebscohost.com/login.aspx?direct=true&db=psyh&AN=2009-01229-006&site=ehost-live) [01229-006Csite=ehost-live](https://search.ebscohost.com/login.aspx?direct=true&db=psyh&AN=2009-01229-006&site=ehost-live). Exclusion reason: Not original study

Bice, H. V. (1954). Some factors that contribute to the concept of self in the child with cerebral palsy. *Ment Hyg*, *38*(1), 120-131. Exclusion reason: Not able to locate study

Birnbaum, R., Lach, L. M., Saposnek, D. T., C MacCulloch, R. (2012). Co-parenting children with neurodevelopmental disorders. In (pp. 270-329). Oxford University Press. https://doi.org/10.1093/med:psych/9780199754021.003.0010. Exclusion reason: Not peer reviewed

Bjorgaas, H. M., Elgen, I., Boe, T., C Hysing, M. (2013). Mental health in children with cerebral palsy: does screening capture the complexity? *ScientificWorldJournal*, *2013*, 468402. <https://doi.org/10.1155/2013/468402>. Exclusion reason: 6-18 years

Bjorgaas, H. M., Elgen, I. B., C Hysing, M. (2021). Trajectories of psychiatric disorders in a cohort of children with cerebral palsy across four years. *Disabil Health J*, *14*(1), 100992. <https://doi.org/10.1016/j.dhjo.2020.100992>. Exclusion reason: 6-18 years

Bjorgaas, H. M., Hysing, M., C Elgen, I. (2012). Psychiatric disorders among children with cerebral palsy at school starting age. *Research in Developmental Disabilities*,

*33*(4), 1287-1293. <https://doi.org/10.1016/j.ridd.2012.02.024>. Exclusion reason:

6-18 years

Bjornson, K. F., Belza, B., Kartin, D., Logsdon, R. G., C McLaughlin, J. (2008). Self- reported health status and quality of life in youth with cerebral palsy and typically developing youth. *Archives of physical medicine and rehabilitation*, *8S*(1), 121-

127. Exclusion reason: 6-18 years

Blacher, J. (1984). Attachment and severely handicapped children: Implications for

intervention. *Journal of Developmental and Behavioral Pediatrics*, *5*(4), 178-183. <https://doi.org/10.1097/00004703-198408000-00004>. Exclusion reason: 6-18 years

Blacher, J., C McIntyre, L. L. (2006). Syndrome specificity and behavioural disorders in young adults with intellectual disability: cultural differences in family impact. *Journal of Intellectual Disability Research*, *50*, 184-198.

<https://doi.org/10.1111/j.1365-2788.2005.00768.x>. Exclusion reason: 6-18 years Blitz, J. M. (1992). *Parental correlates of social competence in preschool children with*

*physical handicaps* ProQuest Information C Learning].

[https://search.ebscohost.com/login.aspx?direct=trueCdb=psyhCAN=1993-](https://search.ebscohost.com/login.aspx?direct=true&db=psyh&AN=1993-74503-001&site=ehost-live) [74503-001Csite=ehost-live](https://search.ebscohost.com/login.aspx?direct=true&db=psyh&AN=1993-74503-001&site=ehost-live). Exclusion reason: Not peer reviewed

Blumberg, M. L. (1979). Character disorders in traumatized and handicapped children.

*Am J Psychother*, *33*(2), 201-203.

https://doi.org/10.1176/appi.psychotherapy.1979.33.2.201. Exclusion reason: Not original study

Boldyreva, U., Streiner, D. L., Rosenbaum, P. L., C Ronen, G. M. (2020). Quality of life in

adolescents with epilepsy, cerebral palsy, and population norms. *Developmental Medicine & Child Neurology*, *c2*(5), 609-614.

<https://doi.org/10.1111/dmcn.14450>. Exclusion reason: 6-18 years

Borkowska, A. R. (2015). Anxiety level and self-esteem in youth with cerebral palsy.

*Current Issues in Personality Psychology*, *3*(3), 159-165.

<https://doi.org/10.5114/cipp.2015.53641>. Exclusion reason: 6-18 years

Boström, P., Broberg, M., C Hwang, C. P. (2010). Different, difficult or distinct? Mothers' and fathers' perceptions of temperament in children with and without

intellectual disabilities. *Journal of Intellectual Disability Research*, *54*(9), 806-

819. <https://doi.org/10.1111/j.1365-2788.2010.01309.x>. Exclusion reason: Mixed sample

Bottcher, L. (2010). Children with spastic cerebral palsy, their cognitive functioning, and social participation: A review. *Child Neuropsychology*, *1c*(3), 209-228.

<https://doi.org/10.1080/09297040903559630>. Exclusion reason: Outcome not socioemotional

Bottcher, L., C Dammeyer, J. (2016). *Development and Learning of Young Children with Disabilities: A Vygotskian Perspective* (Vol. 13). [https://doi.org/10.1007/978-3-](https://doi.org/10.1007/978-3-319-39114-4) [319-39114-4](https://doi.org/10.1007/978-3-319-39114-4). Exclusion reason: Not original study

Boulet, S. L., Boyle, C. A., C Schieve, L. A. (2009). Health care use and health and

functional impact of developmental disabilities among US children, 1997-2005.

*Arch Pediatr Adolesc Med*, *1c3*(1), 19-26.

<https://doi.org/10.1001/archpediatrics.2008.506>. Exclusion reason: Population not CP

Bourke-Taylor, H., Pallant, J. F., Law, M., C Howie, L. (2012). Predicting mental health among mothers of school-aged children with developmental disabilities: The relative contribution of child, maternal and environmental factors. *Research in Developmental Disabilities*, *33*(6), 1732-1740.

<https://doi.org/10.1016/j.ridd.2012.04.011>. Exclusion reason: Outcome not socioemotional

Bouvattier, J. P. (2013). Abord des troubles psychiques dans les paralysies cérébrales de l'enfant = An approach to mental disorders in children with cerebral palsy.

*A.N.A.E. Approche Neuropsychologique des Apprentissages chez l'Enfant*, *25*(5[126-127]), 538-543.

[https://search.ebscohost.com/login.aspx?direct=trueCdb=psyhCAN=2016-](https://search.ebscohost.com/login.aspx?direct=true&db=psyh&AN=2016-15219-009&site=ehost-live) [15219-009Csite=ehost-live](https://search.ebscohost.com/login.aspx?direct=true&db=psyh&AN=2016-15219-009&site=ehost-live). Exclusion reason: Not able to locate study

Breslau, N. (1983). The psychological study of chronically ill and disabled children: Are healthy siblings appropriate controls? *Journal of Abnormal Child Psychology*,

*11*(3), 379-391. <https://doi.org/10.1007/BF00914246>. Exclusion reason: 6-18 years

Breslau, N. (1985). Psychiatric disorder in children with physical disabilities. *Journal of the American Academy of Child Psychiatry*, *24*(1), 87-94.

<https://doi.org/10.1016/S0002-7138(09)60415-5>. Exclusion reason: 6-18 years Breslau, N. (1990). Does brain dysfunction increased children's vulnerability to

environmental stress? *Arch Gen Psychiatry*, *47*(1), 15-20.

<https://doi.org/10.1001/archpsyc.1990.01810130017003>. Exclusion reason: 6-18 years

Breslau, N., C Marshall, I. A. (1985). Psychological disturbance in children with physical disabilities: Continuity and change in a 5-year follow-up. *Journal of Abnormal*

*Child Psychology*, *13*(2), 199-215. <https://doi.org/10.1007/BF00910642>. Exclusion reason: 6-18 years

Britner, P. A., Marvin, R. S., C Pianta, R. C. (2005). Development and preliminary validation of the caregiving behavior system: Association with child attachment classification in the preschool Strange Situation. *Attachment & Human Development*, *7*(1), 83-102. <https://doi.org/10.1080/14616730500039861>.

Exclusion reason: Mixed sample

Britner, P. A. I. V. (1997). *Maternal caregiving behavior and child attachment classifications in the preschool Strange Situation* ProQuest Information C Learning].

[https://search.ebscohost.com/login.aspx?direct=trueCdb=psyhCAN=1997-](https://search.ebscohost.com/login.aspx?direct=true&db=psyh&AN=1997-95004-180&site=ehost-live) [95004-180Csite=ehost-live](https://search.ebscohost.com/login.aspx?direct=true&db=psyh&AN=1997-95004-180&site=ehost-live). Exclusion reason: Not peer reviewed

Brooks-Gunn, J., C Lewis, M. (1982). Temperament and affective interaction in handicapped infants. *Journal of the Division for Early Childhood*, *5*(1), 31-41. Exclusion reason: Mixed sample

Brossard-Racine, M., Hall, N., Majnemer, A., Shevell, M. I., Law, M., Poulin, C., C Rosenbaum, P. (2012). Behavioural problems in school age children with cerebral palsy. *Eur J Paediatr Neurol*, *1c*(1), 35-41.

<https://doi.org/10.1016/j.ejpn.2011.10.001>. Exclusion reason: 6-18 years Brossard-Racine, M., Waknin, J., Shikako-Thomas, K., Shevell, M., Poulin, C., Lach, L.,

Law, M., Schmitz, N., C Majnemer, A. (2013). Behavioral difficulties in

adolescents with cerebral palsy. *Journal of Child Neurology*, *28*(1), 27-33. <https://doi.org/10.1177/0883073812461942>. Exclusion reason: 6-18 years

Brown, F. L., C Whittingham, K. (2015). A structured behavioural family intervention with parents of children with brain injury. In (pp. 60-81). Palgrave Macmillan. [https://search.ebscohost.com/login.aspx?direct=trueCdb=psyhCAN=2015-](https://search.ebscohost.com/login.aspx?direct=true&db=psyh&AN=2015-26756-004&site=ehost-live)

[26756-004Csite=ehost-live](https://search.ebscohost.com/login.aspx?direct=true&db=psyh&AN=2015-26756-004&site=ehost-live). Exclusion reason: Not peer reviewed

Brown, F. L., Whittingham, K., Boyd, R. N., McKinlay, L., C Sofronoff, K. (2014). Improving child and parenting outcomes following paediatric acquired brain injury: A

randomised controlled trial of Stepping Stones Triple P plus acceptance and

commitment therapy. *Journal of Child Psychology and Psychiatry*, *55*(10), 1172- 1183. <https://doi.org/10.1111/jcpp.12227>. Exclusion reason: 6-18 yearspopulation not CP

Brown, F. L., Whittingham, K., Boyd, R. N., McKinlay, L., C Sofronoff, K. (2015). Does Stepping Stones Triple P plus Acceptance and Commitment Therapy improve parent, couple, and family adjustment following paediatric acquired brain injury? A randomised controlled trial. *Behaviour Research and Therapy*, *73*, 58-66. <https://doi.org/10.1016/j.brat.2015.07.001>. Exclusion reason: Outcome not

socioemotional

Brown, F. L., Whittingham, K., McKinlay, L., Boyd, R., C Sofronoff, K. (2013). Efficacy of stepping stones Triple P plus a stress management adjunct for parents of children with an acquired brain injury: The protocol of a randomised controlled trial. *Brain Impairment*, *14*(2), 253-269. <https://doi.org/10.1017/BrImp.2013.18>. Exclusion reason: Not original study

Brunton, L. K., McPhee, P. G., C Gorter, J. W. (2021). Self-reported factors contributing to fatigue and its management in adolescents and adults with cerebral palsy.

*Disability and Rehabilitation: An International, Multidisciplinary Journal*, *43*(7),

929-935. <https://doi.org/10.1080/09638288.2019.1647294>. Exclusion reason: 6-

18 years

Bryan, D. P., C Herjanic, B. (1980). Depression and suicide among adolescents and young adults with selective handicapping conditions. *Exceptional Education Ǫuarterly*, *1*(2), 57-65.

[https://search.ebscohost.com/login.aspx?direct=trueCdb=psyhCAN=1982-](https://search.ebscohost.com/login.aspx?direct=true&db=psyh&AN=1982-07805-001&site=ehost-live) [07805-001Csite=ehost-live](https://search.ebscohost.com/login.aspx?direct=true&db=psyh&AN=1982-07805-001&site=ehost-live). Exclusion reason: Not original study

Burak, M., C Kavlak, E. (2019). Investigation of the relationship between quality of life, activity participation and environmental factors in adolescents with cerebral palsy. *NeuroRehabilitation*, *45*(4), 555-565.

[https://search.ebscohost.com/login.aspx?direct=trueCdb=psyhCAN=2020-](https://search.ebscohost.com/login.aspx?direct=true&db=psyh&AN=2020-15077-008&site=ehost-livekavlake%40hotmail.com)

[15077-008Csite=ehost-livekavlake@hotmail.com](https://search.ebscohost.com/login.aspx?direct=true&db=psyh&AN=2020-15077-008&site=ehost-livekavlake%40hotmail.com). Exclusion reason: 6-18 years Byrt, R. (1969). Monoplegic cerebral palsy with behaviour disorder. *Nurs Times*, *c5*(15),

455-458. Exclusion reason: 6-18 years

Bøttcher, L. (2010). An eye for possibilities in the development of children with cerebral palsy: Neurobiology and neuropsychology in a cultural-historical dynamic understanding. *Outlines: Critical Practice Studies*, *1*, 3-23.

[https://search.ebscohost.com/login.aspx?direct=trueCdb=psyhCAN=2010-](https://search.ebscohost.com/login.aspx?direct=true&db=psyh&AN=2010-23843-002&site=ehost-liveboettcher%40dpu.dk)

[23843-002Csite=ehost-liveboettcher@dpu.dk](https://search.ebscohost.com/login.aspx?direct=true&db=psyh&AN=2010-23843-002&site=ehost-liveboettcher%40dpu.dk). Exclusion reason: Outcome not socioemotional

Bøttcher, L., C Dammeyer, J. (2013). Disability as a risk factor? Development of psychopathology in children with disabilities. *Research in Developmental Disabilities*, *34*(10), 3607-3617. <https://doi.org/10.1016/j.ridd.2013.07.022>. Exclusion reason: Not original study

Caillies, S., Hody, A., C Calmus, A. (2012). Theory of mind and irony comprehension in children with cerebral palsy. *Res Dev Disabil*, *33*(5), 1380-1388.

<https://doi.org/10.1016/j.ridd.2012.03.012>. Exclusion reason: 6-18 years

Can, G., Bilgin, L., Tatli, B., Saydam, R., Coban, A., C Ince, Z. (2012). Morbidity in early adulthood among low-risk very low birth weight children in Turkey: a preliminary study. *Turk J Pediatr*, *54*(5), 458-464. Exclusion reason: Population not CP

Capjon, H., C Bjørk, I. T. (2010). Rehabilitation after multilevel surgery in ambulant spastic children with cerebral palsy: children and parent experiences. *Dev Neurorehabil*, *13*(3), 182-191. <https://doi.org/10.3109/17518421003606151>. Exclusion reason: 6-18 years

Carlsso, M., Olsson, I., Hagberg, G., C Beckung, E. (2008). Behaviour in children with cerebral palsy with and without epilepsy. *Developmental Medicine & Child Neurology*, *50*(10), 784-789. <https://doi.org/10.1111/j.1469-8749.2008.03090.x>. Exclusion reason: 6-18 years

Carona, C., Crespo, C., C Canavarro, M. C. (2013). Similarities amid the difference: Caregiving burden and adaptation outcomes in dyads of parents and their children with and without cerebral palsy. *Research in Developmental*

*Disabilities*, *34*(3), 882-893. <https://doi.org/10.1016/j.ridd.2012.12.004>. Exclusion reason: 6-18 years

Carona, C., Moreira, H., Silva, N., Crespo, C., C Canavarro, M. C. (2014). Social support and adaptation outcomes in children and adolescents with cerebral palsy.

*Disability and Rehabilitation: An International, Multidisciplinary Journal*, *3c*(7), 584-592. <https://doi.org/10.3109/09638288.2013.804596>. Exclusion reason: 6-

18 years

Carona, C., Silva, N., Crespo, C., C Canavarro, M. C. (2014). Caregiving burden and parent–child quality of life outcomes in neurodevelopmental conditions: The mediating role of behavioral disengagement. *Journal of Clinical Psychology in Medical Settings*, *21*(4), 320-328. <https://doi.org/10.1007/s10880-014-9412-5>. Exclusion reason: Mixed sample

Casseus, M., C Cheng, J. (2021). Children with Cerebral Palsy and Unmet Need for Care Coordination. *J Dev Behav Pediatr*, *42*(8), 605-612.

<https://doi.org/10.1097/DBP.0000000000000950>. Exclusion reason: 6-18 years Chang, H.-J., Chiarello, L. A., Palisano, R. J., Orlin, M. N., Bundy, A., C Gracely, E. J.

(2014). The determinants of self-determined behaviors of young children with cerebral palsy. *Research in Developmental Disabilities*, *35*(1), 99-109. <https://doi.org/10.1016/j.ridd.2013.10.004>. Exclusion reason: Outcome not socioemotional

Chen, C.-M., Chen, C.-Y., Wu, K. P., Chen, C.-L., Hsu, H.-C., C Lo, S.-K. (2011). Motor

factors associated with health-related quality-of-life in ambulatory children with cerebral palsy. *American Journal of Physical Medicine & Rehabilitation*, *S0*(11), 940-947. Exclusion reason: 6-18 years

Chen, K.-L., Tseng, M.-H., Shieh, J.-Y., Lu, L., C Huang, C.-Y. (2014). Determinants of

quality of life in children with cerebral palsy: A comprehensive biopsychosocial approach. *Research in Developmental Disabilities*, *35*(2), 520-528.

<https://doi.org/10.1016/j.ridd.2013.12.002>. Exclusion reason: 6-18 years Chen, Y. L., Chen, H. L., Shieh, J. Y., C Wang, T. N. (2019). Preliminary Efficacy of a

Friendly Constraint-Induced Therapy (Friendly-CIT) Program on Motor and Psychosocial Outcomes in Children with Cerebral Palsy. *Phys Occup Ther Pediatr*, *3S*(2), 139-150. <https://doi.org/10.1080/01942638.2018.1484407> Cheong, S. K., Lang, C. P., C Johnston, L. M. (2018). Self-concept of children with

cerebral palsy measured using the population-specific myTREEHOUSE Self- Concept Assessment. *Research in Developmental Disabilities*, *73*, 96-105. <https://doi.org/10.1016/j.ridd.2017.12.001>. Exclusion reason: 6-18 years

Cherry, D. B. (1991). *Relationship between self-esteem and social support in physically disabled and able-bodied adolescents* ProQuest Information C Learning]. [https://search.ebscohost.com/login.aspx?direct=trueCdb=psyhCAN=1992-](https://search.ebscohost.com/login.aspx?direct=true&db=psyh&AN=1992-73638-001&site=ehost-live)

[73638-001Csite=ehost-live](https://search.ebscohost.com/login.aspx?direct=true&db=psyh&AN=1992-73638-001&site=ehost-live). Exclusion reason: Not peer reviewed

Chiarello, L. A., Almasri, N., C Palisano, R. J. (2009). Factors related to adaptive behavior in children with cerebral palsy. *Journal of Developmental and Behavioral*

*Pediatrics*, *30*(5), 426-434. <https://doi.org/10.1097/DBP.0b013e3181b4ec54>. Exclusion reason: 6-18 years

Choi, J. Y., Rha, D.-w., C Park, E. S. (2016). The effects of the severity of periventricular

leukomalacia on the neuropsychological outcomes of preterm children. *Journal of Child Neurology*, *31*(5), 603-612. <https://doi.org/10.1177/0883073815604229>. Exclusion reason: Population not CP

Chong, J., Mackey, A. H., Stott, N. S., C Broadbent, E. (2013). Walking drawings and walking ability in children with cerebral palsy. *Health Psychol*, *32*(6), 710-713. <https://doi.org/10.1037/a0027353>. Exclusion reason: 6-18 years

Clark, C., Sliker, L., Sandstrum, J., Burne, B., Haggett, V., C Bodine, C. (2019).

Development and preliminary investigation of a semiautonomous Socially

Assistive Robot (SAR) designed to elicit communication, motor skills, emotion, and visual regard (engagement) from young children with complex cerebral

palsy: A pilot comparative trial. *Advances in Human-Computer Interaction*, *201S*. <https://doi.org/10.1155/2019/2614060>. Exclusion reason: Outcome not

socioemotional

Clements, M., C Barnett, D. (2002). Parenting and attachment among toddlers with congenital anomalies: Examining the strange situation and attachment Q-sort. *Infant mental health journal*, *23*(6), 625-642. <https://doi.org/10.1002/imhj.10040>. Exclusion reason: Mixed sample

Cohen, E., Biran, G., Aran, A., C Gross-Tsur, V. (2008). Locus of control, perceived parenting style, and anxiety in children with cerebral palsy. *Journal of Developmental and Physical Disabilities*, *20*(5), 415-423.

<https://doi.org/10.1007/s10882-008-9106-8>. Exclusion reason: 6-18 years Cole, P. G. (1976). Parents' and teachers' estimates of the social competence of

handicapped and normal children. *Australian Journal of Mental Retardation*, *4*(4), 1-8. <https://doi.org/10.3109/13668257609004304>. Exclusion reason: 6-18 years

Colver, A. (2006). Study protocol: SPARCLE--a multi-centre European study of the relationship of environment to participation and quality of life in children with

cerebral palsy. *BMC Public Health*, *c*, 105. [https://doi.org/10.1186/1471-2458-6-](https://doi.org/10.1186/1471-2458-6-105)

[105](https://doi.org/10.1186/1471-2458-6-105). Exclusion reason: Not original study

Colver, A. (2010). Why are children with cerebral palsy more likely to have emotional and behavioural difficulties? *Dev Med Child Neurol*, *52*(11), 986.

<https://doi.org/10.1111/j.1469-8749.2010.03721.x>. Exclusion reason: 6-18 years Colver, A., Rapp, M., Eisemann, N., Ehlinger, V., Thyen, U., Dickinson, H. O., Parkes, J.,

Parkinson, K., Nystrand, M., Fauconnier, J., Marcelli, M., Michelsen, S. I., C

Arnaud, C. (2015). Self-reported quality of life of adolescents with cerebral palsy: A cross-sectional and longitudinal analysis. *The Lancet*, *385*(9969), 705-716. <https://doi.org/10.1016/S0140-6736(14)61229-0>. Exclusion reason: 6-18 years

Colver, A. F., C Dickinson, H. O. (2010). Study protocol: determinants of participation and quality of life of adolescents with cerebral palsy: a longitudinal study

(SPARCLE2). *BMC Public Health*, *10*, 280. [https://doi.org/10.1186/1471-2458-10-](https://doi.org/10.1186/1471-2458-10-280)

[280](https://doi.org/10.1186/1471-2458-10-280). Exclusion reason: 6-18 years

Craven, C., James, A., C Murphy, M. (2002). Cerebral palsy and juvenile-onset bipolar disorder: A preliminary report. *European Child & Adolescent Psychiatry*, *11*(3), 134-137. <https://doi.org/10.1007/s00787-002-0235-9>. Exclusion reason:

Outcome not socioemotional

Craven, C., C Murphy, M. (2000). Carbamazepine treatment of bipolar disorder in an

adolescent with cerebral palsy. *J Am Acad Child Adolesc Psychiatry*, *3S*(6), 680- 681. <https://doi.org/10.1097/00004583-200006000-00005>. Exclusion reason: Outcome not socioemotional

Cunningham, S. D., Thomas, P. D., C Warschausky, S. (2007). Gender differences in peer relations of children with neurodevelopmental conditions. *REHABILITATION*

*PSYCHOLOGY*, *52*(3), 331-337. <https://doi.org/10.1037/0090-5550.52.3.331>.

Exclusion reason: 6-18 years

Cunningham, S. D., Warschausky, S., C Thomas, P. D. (2009). Parenting and Social Functioning of Children With and Without Cerebral Palsy. *REHABILITATION PSYCHOLOGY*, *54*(1), 109-115. <https://doi.org/10.1037/a0014748>. Exclusion reason: Mixed sample

Dababneh, K. A. H. (2013). The socio-emotional behavioural problems of children with cerebral palsy according to their parents' perspectives. *International Journal of Adolescence and Youth*, *18*(2), 85-104.

<https://doi.org/10.1080/02673843.2012.655443>. Exclusion reason: 6-18 years

Dahan-Oliel, N., Shikako-Thomas, K., C Majnemer, A. (2012). Quality of life and leisure participation in children with neurodevelopmental disabilities: A thematic analysis of the literature. *Ǫuality of Life Research: An International Journal of Ǫuality of Life Aspects of Treatment, Care & Rehabilitation*, *21*(3), 427-439. <https://doi.org/10.1007/s11136-011-0063-9>. Exclusion reason: Not original study

Dahl, L. B., Kaaresen, P. I., Tunby, J., Handegård, B. H., Kvernmo, S., C Rønning, J. A. (2006). Emotional, behavioral, social, and academic outcomes in adolescents born with very low birth weight. *Pediatrics*, *118*(2), e449-459.

<https://doi.org/10.1542/peds.2005-3024>. Exclusion reason: Population not CP Dahlgren, S., Sandberg, A. D., C Larsson, M. (2010). Theory of mind in children with

severe speech and physical impairments. *Research in Developmental Disabilities*, *31*(2), 617-624. <https://doi.org/10.1016/j.ridd.2009.12.010>. Exclusion reason: 6-18 years

Dallas, E., Stevenson, J., C McGurk, H. (1993). Cerebral-palsied children's interactions with siblings: II Interactional structure. *Child Psychology & Psychiatry & Allied Disciplines*, *34*(5), 649-671. <https://doi.org/10.1111/j.1469-7610.1993.tb01063.x>. Exclusion reason: 6-18 years

Dallas, E., Stevenson, J., C McGurk, H. (1993). CEREBRAL-PALSIED CHILDRENS

INTERACTIONS WITH SIBLINGS .1. INFLUENCE OF SEVERITY OF DISABILITY, AGE AND BIRTH-ORDER. *JOURNAL OF CHILD PSYCHOLOGY AND PSYCHIATRY AND ALLIED DISCIPLINES*, *34*(5), 621-647. [https://doi.org/10.1111/j.1469-](https://doi.org/10.1111/j.1469-7610.1993.tb01062.x)

[7610.1993.tb01062.x](https://doi.org/10.1111/j.1469-7610.1993.tb01062.x). Exclusion reason: Not able to locate study Dammeyer, J., C Bottcher, L. (2010). Handicap som risikofaktor? Et

udviklingspsykopatologisk perspektiv på born med handicap = Disability as a risk factor? A developmental psychopathology perspective on children with

disabilities. *Psyke & Logos*, *31*(2), 668-687.

https://search.ebscohost.com/login.aspx?direct=trueCdb=psyhCAN=2011- 01623-015Csite=ehost-live. Exclusion reason: Not original study

Dang, V. M., Colver, A., Dickinson, H. O., Marcelli, M., Michelsen, S. I., Parkes, J., Parkinson, K., Rapp, M., Arnaud, C., Nystrand, M., C Fauconnier, J. (2015).

Predictors of participation of adolescents with cerebral palsy: A European multi- centre longitudinal study. *Research in Developmental Disabilities*, *3c*, 551-564. <https://doi.org/10.1016/j.ridd.2014.10.043>. Exclusion reason: 6-18 years

Danilchenko, D. A., C Kazmin, A. M. (2015). Functionality, temperament and

development of personality in preschool children: a pilot study. *CLINICAL*

*PSYCHOLOGY AND SPECIAL EDUCATION*, *4*(1), 64-74. Exclusion reason: Mixed

sample

Das, S., Aggarwal, A., Roy, S., C Kumar, P. (2017). Quality of life in Indian children with cerebral palsy using cerebral palsy-quality of life questionnaire. *Journal of pediatric neurosciences*, *12*(3), 251. Exclusion reason: 6-18 years

Davis, E., Davies, B., Wolfe, R., Raadsveld, R., Heine, B., Thomason, P., Dobson, F., C Graham, H. K. (2009). A randomized controlled trial of the impact of therapeutic horse riding on the quality of life, health, and function of children with cerebral palsy. *Developmental Medicine & Child Neurology*, *51*(2), 111-119.

<https://doi.org/10.1111/j.1469-8749.2008.03245.x>. Exclusion reason: 6-18 years Davis, E., Mackinnon, A., C Waters, E. (2012). Parent proxy-reported quality of life for

children with cerebral palsy: is it related to parental psychosocial distress?

*CHILD CARE HEALTH AND DEVELOPMENT*, *38*(4), 553-560.

<https://doi.org/10.1111/j.1365-2214.2011.01267.x>. Exclusion reason: 6-18 years Davis, E., Reddihough, D., Murphy, N., Epstein, A., Reid, S. M., Whitehouse, A., Williams,

K., Leonard, H., C Downs, J. (2017). Exploring quality of life of children with cerebral palsy and intellectual disability: What are the important domains of life? *Child: Care, Health and Development*, *43*(6), 854-860.

<https://doi.org/10.1111/cch.12501>. Exclusion reason: 6-18 years

Davis, E., Shelly, A., Waters, E., C Davern, M. (2010). Measuring the quality of life of children with cerebral palsy: Comparing the conceptual differences and

psychometric properties of three instruments. *Developmental Medicine & Child Neurology*, *52*(2), 174-180. <https://doi.org/10.1111/j.1469-8749.2009.03382.x>. Exclusion reason: 6-18 years

Davis, E., Shelly, A., Waters, E., MacKinnon, A., Reddihough, D., Boyd, R., C Graham, H.

K. (2009). Quality of life of adolescents with cerebral palsy: Perspectives of

adolescents and parents. *Developmental Medicine & Child Neurology*, *51*(3), 193-199. <https://doi.org/10.1111/j.1469-8749.2008.03194.x>. Exclusion reason: 6-18 years

De Clercq, L., Van der Kaap-Deeder, J., Dieleman, L. M., Soenens, B., Prinzie, P., C De

Pauw, S. S. W. (2019). Parenting and psychosocial development in youth with and without autism spectrum disorder, cerebral palsy, and Down syndrome: A cross- disability comparison. *Advances in Neurodevelopmental Disorders*, *3*(2), 220-

234. <https://doi.org/10.1007/s41252-019-00112-2>. Exclusion reason: 6-18 years De Clercq, L. E., Soenens, B., Dieleman, L. M., Prinzie, P., Van der Kaap-deeder, J.,

Beyers, W., C De Pauw, S. S. W. (2022). Parenting and Child Personality as

Modifiers of the Psychosocial Development of Youth with Cerebral Palsy. *CHILD PSYCHIATRY & HUMAN DEVELOPMENT*, *53*(1), 137-155.

<https://doi.org/10.1007/s10578-020-01106-1>. Exclusion reason: 6-18 years Dean, K. R. (2000). *Reciprocal imitation and social responsiveness in children with*

*physical, health, or developmental impairment* ProQuest Information C Learning].

[https://search.ebscohost.com/login.aspx?direct=trueCdb=psyhCAN=2000-](https://search.ebscohost.com/login.aspx?direct=true&db=psyh&AN=2000-95020-263&site=ehost-live) [95020-263Csite=ehost-live](https://search.ebscohost.com/login.aspx?direct=true&db=psyh&AN=2000-95020-263&site=ehost-live). Exclusion reason: Not peer reviewed

Della Ratta, F. (2018). Lo sviluppo emozionale e il disturbo percettivo nelle paralisi cerebrali infantili Spunti di riflessione in un contesto riabilitativo = Perceptual disorders and emotional development in children with cerebral palsy Starting

points for reflection in a reha. *Richard e Piggle: Studi Psicoanalitici del Bambino e dell’Adolexcente*, *2c*(2), 113-122.

https://search.ebscohost.com/login.aspx?direct=trueCdb=psyhCAN=2019-

[01187-001Csite=ehost-livefeliciana.dellaratta@yahoo.it.](mailto:01187-001Csite%3Dehost-livefeliciana.dellaratta@yahoo.it) Exclusion reason: Do not report relevant association or prevalence

Demuth, S. K., Knutson, L. M., C Fowler, E. G. (2012). The PEDALS stationary cycling

intervention and health‐related quality of life in children with cerebral palsy: A randomized controlled trial. *Developmental Medicine & Child Neurology*, *54*(7), 654-661. <https://doi.org/10.1111/j.1469-8749.2012.04321.x>. Exclusion reason: 6-18 years

Dickinson, H. O., Parkinson, K. N., Ravens-Sieberer, U., Schirripa, G., Thyen, U., Arnaud, C., Beckung, E., Fauconnier, J., McManus, V., Michelsen, S. I., Parkes, J., C

Colver, A. F. (2007). Self-reported quality of life of 8-12-year-old children with cerebral palsy: A cross-sectional European study. *The Lancet*, *3cS*(9580), 2171- 2178. <https://doi.org/10.1016/S0140-6736(07)61013-7>. Exclusion reason: 6-18 years

Didden, R., Korzilius, H., van Aperlo, B., van Overloop, C., C de Vries, M. (2002). Sleep problems and daytime problem behaviours in children with intellectual disability. *Journal of Intellectual Disability Research*, *4c*(7), 537-547.

<https://doi.org/10.1046/j.1365-2788.2002.00404.x>. Exclusion reason: Population not CP

Dieleman, L. M., Soenens, B., Prinzie, P., De Clercq, L., Ortibus, E., C De Pauw, S. S. W. (2021). Daily parenting of children with cerebral palsy: The role of daily child

behavior, parents' daily psychological needs, and mindful parenting.

*Development and psychopathology*, *33*(1), 184-200.

<https://doi.org/10.1017/S0954579419001688>. Exclusion reason: Outcome not socioemotional

DiFazio, R. L., Vessey, J. A., Miller, P. E., Snyder, B. D., C Shore, B. J. (2022). Health‐ related quality of life and caregiver burden after hip reconstruction and spinal fusion in children with spastic cerebral palsy. *Developmental Medicine & Child*

*Neurology*, *c4*(1), 80-87. <https://doi.org/10.1111/dmcn.14994>. Exclusion reason: 6-18 years

Difazio, R. L., Vessey, J. A., Zurakowski, D., C Snyder, B. D. (2016). Differences in health‐ related quality of life and caregiver burden after hip and spine surgery in non‐ ambulatory children with severe cerebral palsy. *Developmental Medicine & Child Neurology*, *58*(3), 298-305. <https://doi.org/10.1111/dmcn.12872>. Exclusion

reason: 6-18 years

Dmitruk, E., Mirska, A., Kułak, W., Kalinowska, A. K., Okulczyk, K., C Wojtkowski, J. (2014). Psychometric properties and validation of the Polish CP QOL-Child questionnaire: A pilot study. *Scandinavian Journal of Caring Sciences*, *28*(4), 878-884. <https://doi.org/10.1111/scs.12095>. Exclusion reason: 6-18 years

Dobhal, M., Juneja, M., Jain, R., Sairam, S., C Thiagarajan, D. (2014). Health-related

quality of life in children with cerebral palsy and their families. *Indian pediatrics*, *51*, 385-387. Exclusion reason: 6-18 years

Dorval, G., Tetreault, S., C Caron, C. (1996). Impact of aquatic programmes on

adolescents with cerebral palsy. *Occupational Therapy International*, *3*(4), 241-

261. <https://doi.org/10.1002/oti.39>. Exclusion reason: 6-18 years

Downs, J., Blackmore, A. M., Epstein, A., Skoss, R., Langdon, K., Jacoby, P., Whitehouse,

A. J. O., Leonard, H., Rowe, P. W., C Glasson, E. J. (2018). The prevalence of mental health disorders and symptoms in children and adolescents with cerebral palsy: A systematic review and meta‐analysis. *Developmental Medicine & Child Neurology*, *c0*(1), 30-38. <https://doi.org/10.1111/dmcn.13555>. Exclusion reason: 6-18 years

Downs, J., Jacoby, P., Leonard, H., Epstein, A., Murphy, N., Davis, E., Reddihough, D., Whitehouse, A., C Williams, K. (2019). Psychometric properties of the Quality of Life Inventory-Disability (QI-Disability) measure. *Ǫuality of Life Research: An International Journal of Ǫuality of Life Aspects of Treatment, Care &*

*Rehabilitation*, *28*(3), 783-794. <https://doi.org/10.1007/s11136-018-2057-3>. Exclusion reason: 6-18 years

Dunst, C. J., Raab, M., Trivette, C. M., Parkey, C., Gatens, M., Wilson, L. L., French, J., C Hamby, D. W. (2007). Child and adult social-emotional benefits of response- contingent child learning opportunities. *Journal of Early and Intensive Behavior Intervention*, *4*(2), 379-391. <https://doi.org/10.1037/h0100380>. Exclusion reason: Outcome not socioemotional

Durán-Carabali, L. E., Henao-Pacheco, M. L., González-Clavijo, A. M., C Dueñas, Z. (2021). Salivary alpha amylase and cortisol levels as stress biomarkers in children with cerebral palsy and their association with a physical therapy program. *Res Dev Disabil*, *108*, 103807.

<https://doi.org/10.1016/j.ridd.2020.103807>. Exclusion reason: Outcome not socioemotional

Durukan, İ., Ceylan, M. F., Kara, K., Erdem, M., Akça, Ö. F., C Türkay, T. (2011). Mental retardasyonu olan çocuklarda yaşam kalitesi = Quality of life in children with mental retardation. *Yeni Symposium: psikiyatri, nöroloji ve davraniş bilimleri dergisi*, *4S*(1), 43-50.

[https://search.ebscohost.com/login.aspx?direct=trueCdb=psyhCAN=2011-](https://search.ebscohost.com/login.aspx?direct=true&db=psyh&AN=2011-07756-004&site=ehost-liveidurukan2003%40yahoo.com)

[07756-004Csite=ehost-liveidurukan2003@yahoo.com](https://search.ebscohost.com/login.aspx?direct=true&db=psyh&AN=2011-07756-004&site=ehost-liveidurukan2003%40yahoo.com). Exclusion reason: 6-18 years

Elgen, S. K., Leversen, K. T., Grundt, J. H., Hurum, J., Sundby, A. B., Elgen, I. B., C

Markestad, T. (2012). Mental health at 5 years among children born extremely preterm: A national population-based study. *European Child & Adolescent Psychiatry*, *21*(10), 583-589. <https://doi.org/10.1007/s00787-012-0298-1>.

Exclusion reason: Population not CP

Ellison, P. H. (1984). Neurologic development of the high-risk infant. *Clin Perinatol*, *11*(1), 41-58. Exclusion reason: Outcome not socioemotional

Engel, J. M., Wilson, S., Tran, S. T., Jensen, M. P., C Ciol, M. A. (2013). Pain

Catastrophizing in Youths With Physical Disabilities and Chronic Pain. *Journal of Pediatric Psychology*, *38*(2), 192-201. <https://doi.org/10.1093/jpepsy/jss103>.

Exclusion reason: Mixed sample

Festante, F., Antonelli, C., Chorna, O., Corsi, G., C Guzzetta, A. (2019). Parent-infant

interaction during the first year of life in infants at high risk for cerebral palsy: A systematic review of the literature. *Neural Plasticity*, *201S*.

<https://doi.org/10.1155/2019/5759694>. Exclusion reason: Not original study Fevang, S. K. E., Hysing, M., Markestad, T., C Sommerfelt, K. (2016). Mental health in

children born extremely preterm without severe neurodevelopmental disabilities. *Pediatrics*, *137*(4), 1-11.

[https://search.ebscohost.com/login.aspx?direct=trueCdb=psyhCAN=2016-](https://search.ebscohost.com/login.aspx?direct=true&db=psyh&AN=2016-23930-004&site=ehost-livesilje.elgen%40uib.no)

[23930-004Csite=ehost-livesilje.elgen@uib.no](https://search.ebscohost.com/login.aspx?direct=true&db=psyh&AN=2016-23930-004&site=ehost-livesilje.elgen%40uib.no). Exclusion reason: Population not CP

Fevang, S. K. E., Hysing, M., Sommerfelt, K., C Elgen, I. (2017). Mental health assessed by the Strengths and Difficulties Questionnaire for children born extremely preterm without severe disabilities at 11 years of age: A Norwegian, national population‐based study. *European Child & Adolescent Psychiatry*, *2c*(12), 1523- 1531. <https://doi.org/10.1007/s00787-017-1007-x>. Exclusion reason: Population not CP

Fisher, M. (2001). Andre's story: Frames of friendship. In (pp. 91-111). Paul H Brookes Publishing.

[https://search.ebscohost.com/login.aspx?direct=trueCdb=psyhCAN=2001-](https://search.ebscohost.com/login.aspx?direct=true&db=psyh&AN=2001-18916-006&site=ehost-live) [18916-006Csite=ehost-live](https://search.ebscohost.com/login.aspx?direct=true&db=psyh&AN=2001-18916-006&site=ehost-live). Exclusion reason: Not peer reviewed

Florou, A., Widdershoven, M.-A., Giannakopoulos, G., C Christogiorgos, S. (2016). Working through physical disability in psychoanalytic psychotherapy with an adolescent boy. *Psychoanalytical Social Work*, *23*(2), 119-129.

<https://doi.org/10.1080/15228878.2016.1160834>. Exclusion reason: 6-18 years Foster, T., Rai, A. I. K., Weller, R. A., Dixon, T. A., C Weller, E. B. (2010). Psychiatric

Complications in Cerebral Palsy. *CURRENT PSYCHIATRY REPORTS*, *12*(2), 116-

121. <https://doi.org/10.1007/s11920-010-0096-8>. Exclusion reason: 6-18 years Freeman, R. D. (1970). Psychiatric problems in adolescents with cerebral palsy. *Dev*

*Med Child Neurol*, *12*(1), 64-70. [https://doi.org/10.1111/j.1469-](https://doi.org/10.1111/j.1469-8749.1970.tb01861.x) [8749.1970.tb01861.x](https://doi.org/10.1111/j.1469-8749.1970.tb01861.x). Exclusion reason: 6-18 years

French, N. P., Hagan, R., Evans, S. F., Mullan, A., C Newnham, J. P. (2004). Repeated antenatal corticosteroids: effects on cerebral palsy and childhood behavior. *Am J Obstet Gynecol*, *1S0*(3), 588-595. <https://doi.org/10.1016/j.ajog.2003.12.016>.

Exclusion reason: Population not CP

Frey, C., C Schneider, R. (1987). [Long-term development of children with cerebral palsy. Follow-up at age 15]. *Z Kinder Jugendpsychiatr*, *15*(2), 134-145. Exclusion reason: 6-18 years

Frontini, R., Crespo, C., Carona, C., C Canavarro, M. C. (2012). Health-related quality of life and its correlates in children with cerebral palsy: An exploratory study.

*Journal of Developmental and Physical Disabilities*, *24*(2), 181-196. <https://doi.org/10.1007/s10882-011-9265-x>. Exclusion reason: 6-18 years

Galambos, N. L., Magill-Evans, J., C Darrah, J. (2008). Psychosocial Maturity in the Transition to Adulthood for People With and Without Motor Disabilities.

*REHABILITATION PSYCHOLOGY*, *53*(4), 498-504.

<https://doi.org/10.1037/a0013131>. Exclusion reason: 6-18 years

Gallart Capdevila, J. M. (1973). Psychological aspects of children suffering from cerebral palsy: Spastic and/or nonspastic. *Anuario de Psicología*, *8*, 53-71. [https://search.ebscohost.com/login.aspx?direct=trueCdb=psyhCAN=1975-](https://search.ebscohost.com/login.aspx?direct=true&db=psyh&AN=1975-11990-001&site=ehost-live) [11990-001Csite=ehost-live](https://search.ebscohost.com/login.aspx?direct=true&db=psyh&AN=1975-11990-001&site=ehost-live) Exclusion reason: Not able to locate study

García-Galant, M., Blasco, M., Reid, L., Pannek, K., Leiva, D., Laporta-Hoyos, O., Ballester-Plané, J., Miralbell, J., Caldú, X., Alonso, X., Toro-Tamargo, E.,

Meléndez-Plumed, M., Gimeno, F., Coronas, M., Soro-Camats, E., Boyd, R., C Pueyo, R. (2020). Study protocol of a randomized controlled trial of home-based computerized executive function training for children with cerebral palsy. *BMC Pediatr*, *20*(1), 9. <https://doi.org/10.1186/s12887-019-1904-x>. Exclusion reason: Not original study

Gardiner, E., Miller, A. R., C Lach, L. M. (2018). Family impact of childhood

neurodevelopmental disability: considering adaptive and maladaptive behaviour. *Journal of Intellectual Disability Research*, *c2*(10), 888-899. <https://doi.org/10.1111/jir.12547>. Exclusion reason: Mixed sample

Gardiner, E., Miller, A. R., C Lach, L. M. (2020). Topography of behavior problems among children with neurodevelopmental conditions: Profile differences and overlaps. *Child: Care, Health and Development*, *4c*(1), 149-153.

<https://doi.org/10.1111/cch.12720>. Exclusion reason: 6-18 years

Garg, P., Haynes, N., De Lima, J., C Collins, J. J. (2017). Profile of children with

developmental disabilities attending a complex pain clinic of a children's

hospital in Australia. *Journal of Paediatrics and Child Health*, *53*(12), 1186-1191. <https://doi.org/10.1111/jpc.13633>. Exclusion reason: Mixed sample

Gatta, M., Sisti, M., Brunello, G., Sale, E., Simonelli, A., C Battistella, P. A. (2014). Valutazione delle relazioni familiari nell’intervento psicomotorio con pre-

adolescenti affetti da paralisi cerebrale = Evaluation of family relationships in psychomotor intervention with pre-teens suffering from cerebral palsy. *Giornale di Neuropsichiatria dell'Età Evolutiva*, *34*(1), 73-81.

[https://search.ebscohost.com/login.aspx?direct=trueCdb=psyhCAN=2014-](https://search.ebscohost.com/login.aspx?direct=true&db=psyh&AN=2014-23310-009&site=ehost-livesisti.marta%40gmail.com)

[23310-009Csite=ehost-livesisti.marta@gmail.com](https://search.ebscohost.com/login.aspx?direct=true&db=psyh&AN=2014-23310-009&site=ehost-livesisti.marta%40gmail.com). Exclusion reason: 6-18 years Gillberg, C. (2020). Mental health problems in cerebral palsy: comprehensive

management for children and their families. *Dev Med Child Neurol*, *c2*(2), 154. <https://doi.org/10.1111/dmcn.14359>. Exclusion reason: Not original study

Giusti, L., Provenzi, L., C Montirosso, R. (2018). The Face-to-Face Still-Face (FFSF) Paradigm in Clinical Settings: Socio-Emotional Regulation Assessment and Parental Support With Infants With Neurodevelopmental Disabilities. *Frontiers in psychology*, *S*. <https://doi.org/10.3389/fpsyg.2018.00789>. Exclusion reason: Mixed sample

Golden, G. S. (1979). The effect of developmental disabilities on mental health. *J Sch Health*, *4S*(5), 260-262. <https://doi.org/10.1111/j.1746-1561.1979.tb03847.x>. Exclusion reason: Not original study

Gover, S. M. (1979). Nursing care study: a personality disorder in an adolescent with cerebral palsy. *Nurs Times*, *75*(49), 2111-2114. Exclusion reason: 6-18 years

Govindshenoy, M., C Spencer, N. (2007). Abuse of the disabled child: A systematic

review of population-based studies. *Child: Care, Health and Development*, *33*(5), 552-558. <https://doi.org/10.1111/j.1365-2214.2006.00693.x>. Exclusion reason: Not original study

Graham, P., C Rutter, M. (1968). Organic brain dysfunction and child psychiatric

disorder. *Br Med J*, *3*(5620), 695-700. <https://doi.org/10.1136/bmj.3.5620.695>. Exclusion reason: Mixed sample

Grody, M. B., C Coffey, B. J. (2012). Presentation and treatment of acute psychosis in an adolescent girl with cerebral palsy. *Journal of Child and Adolescent*

*Psychopharmacology*, *22*(2), 175-178. <https://doi.org/10.1089/cap.2012.2223>. Exclusion reason: 6-18 years

Guyard, A., Michelsen, S. I., Arnaud, C., C Fauconnier, J. (2017). Family adaptation to cerebral palsy in adolescents: A european multicenter study. *Research in Developmental Disabilities*, *c1*, 138-150.

<https://doi.org/10.1016/j.ridd.2016.11.010>. Exclusion reason: Outcome not socioemotional

Hall, L. J., C McGregor, J. A. (2000). A follow-up study of the peer relationships of children with disabilities in an inclusive school. *The Journal of Special Education*, *34*(3), 114-126. <https://doi.org/10.1177/002246690003400301>. Exclusion

reason: 6-18 years

Hamer, E. G., Bos, A. F., C Hadders-Algra, M. (2016). Specific characteristics of abnormal general movements are associated with functional outcome at school age. *Early Human Development*, *S5*, 9-13.

<https://doi.org/10.1016/j.earlhumdev.2016.01.019>. Exclusion reason: Mixed sa mple

Hanes, J. E., Hlyva, O., Rosenbaum, P., Freeman, M., Nguyen, T., Palisano, R. J., C

Gorter, J. W. (2019). Beyond stereotypes of cerebral palsy: Exploring the lived experiences of young Canadians. *Child: Care, Health and Development*, *45*(5), 613-622. <https://doi.org/10.1111/cch.12705>. Exclusion reason: 6-18 years

Hansen, J. M. (1995). *Social self-concept in children with physical disabilities: Exploring the role of friendship* ProQuest Information C Learning].

[https://search.ebscohost.com/login.aspx?direct=trueCdb=psyhCAN=1995-](https://search.ebscohost.com/login.aspx?direct=true&db=psyh&AN=1995-95005-019&site=ehost-live) [95005-019Csite=ehost-live](https://search.ebscohost.com/login.aspx?direct=true&db=psyh&AN=1995-95005-019&site=ehost-live). Exclusion reason: Not peer reviewed

Hanzlik, J. R., C Stevenson, M. B. (1986). Interaction of mothers with their infants who are mentally retarded, retarded with cerebral palsy, or nonretarded. *Am J Ment Defic*, *S0*(5), 513-520. Exclusion reason: Mixed sample

Harris, J. (2008). Brain disorders and their effect on psychopathology. In (pp. 459-473).

Wiley Blackwell. <https://doi.org/10.1002/9781444300895.ch30>. Exclusion reason: Not peer reviewed

Harvey, D., C Greenway, P. (1982). Congruence between mother and handicapped child's view of the child's sense of adjustment. *Exceptional Child*, *2S*(2), 111-116. <https://doi.org/10.1080/0156655820290206>. Exclusion reason: 6-18 years

Hauer, J. (2019). Pain and irritability. In (pp. 227-238). Mac Keith Press. [https://search.ebscohost.com/login.aspx?direct=trueCdb=psyhCAN=2018-](https://search.ebscohost.com/login.aspx?direct=true&db=psyh&AN=2018-31846-014&site=ehost-live) [31846-014Csite=ehost-live](https://search.ebscohost.com/login.aspx?direct=true&db=psyh&AN=2018-31846-014&site=ehost-live). Exclusion reason: Not peer reviewed

Hauser-Cram, P., Warfield, M. E., Shonkoff, J. P., Krauss, M. W., Sayer, A., Upshur, C. C., C Hodapp, R. M. (2001). Children with disabilities: A longitudinal study of child development and parent well-being. *Monographs of the Society for Research in Child Development*, i-126. Exclusion reason: Mixed sample

Heflich-Piatkowska, H. (1968). [Multifactor analysis of psychologic conditions in children with infantile cerebral palsies during rehabilitation]. *Psychiatr Neurol Med Psychol Beih*, *8-S*, 160-162. Exclusion reason: Not able to locate study

Heller, K. W., Alberto, P. A., C Meagher, T. M. (1996). The impact of physical impairments on academic performance. *Journal of Developmental and Physical Disabilities*, *8*(3), 233-245. <https://doi.org/10.1007/BF02578392>. Exclusion reason: Mixed

sample

Hendriks, A. H. C., De Moor, J. M. H., Oud, J. H. L., Franken, W. M., C Savelberg, M. M. H.

W. (2001). Behaviour problems of young motor disabled children at home and in the therapeutic toddler class. *European Journal of Special Needs Education*,

*1c*(1), 15-28. <https://doi.org/10.1080/08856250150501770>. Exclusion reason: Mixed sample

Hersov, L. (1963). EMOTIONAL FACTORS IN CEREBRAL PALSY. *Dev Med Child Neurol*,

*25*, 504-511. <https://doi.org/10.1111/j.1469-8749.1963.tb10705.x>. Exclusion reason: Not able to locate study

Herzberg, B., C Herzberg, L. (1977). Brain damage and abnormal behaviour in children.

*Med J Aust*, *1*(23), 853-855. [https://doi.org/10.5694/j.1326-](https://doi.org/10.5694/j.1326-5377.1977.tb131173.x) [5377.1977.tb131173.x](https://doi.org/10.5694/j.1326-5377.1977.tb131173.x). Exclusion reason: Population not CP

Hicks, M., C Davitt, K. (2018). Chronic illness and rehabilitation. In (pp. 446-491).

Charles C Thomas Publisher, Ltd.

[https://search.ebscohost.com/login.aspx?direct=trueCdb=psyhCAN=2018-](https://search.ebscohost.com/login.aspx?direct=true&db=psyh&AN=2018-18845-014&site=ehost-live) [18845-014Csite=ehost-live](https://search.ebscohost.com/login.aspx?direct=true&db=psyh&AN=2018-18845-014&site=ehost-live). Exclusion reason: Not peer reviewed

Hinton, V. J., Nereo, N. E., Fee, R. J., C Cyrulnik, S. E. (2006). Social Behavior Problems in Boys with Duchenne Muscular Dystrophy. *Journal of Developmental and*

*Behavioral Pediatrics*, *27*(6), 470-476. [https://doi.org/10.1097/00004703-](https://doi.org/10.1097/00004703-200612000-00003) [200612000-00003](https://doi.org/10.1097/00004703-200612000-00003). Exclusion reason: Population not CP

Hirst, M. (1989). Patterns of impairment and disability related to social handicap in young people with cerebral palsy and spina bifida. *Journal of Biosocial Science*, *21*(1), 1-12. <https://doi.org/10.1017/S0021932000017685>. Exclusion reason: Mixed sample

Ho, S. M., Fung, B. K., Fung, A. S., Chow, S. P., Ip, W. Y., Lee, S. F., Leung, E. Y., C Ha, K.

W. (2008). Overprotection and the psychological states of cerebral palsy patients and their caretakers in Hong Kong: a preliminary report. *Hong Kong Med J*, *14*(4), 286-291. Exclusion reason: 6-18 years

Hollung, S. J., Bakken, I. J., Vik, T., Lydersen, S., Wiik, R., Aaberg, K. M., C Andersen, G. L. (2020). Comorbidities in cerebral palsy: A patient registry study. *Developmental Medicine & Child Neurology*, *c2*(1), 97-103. <https://doi.org/10.1111/dmcn.14307>. Exclusion reason: 6-18 years

Horwood, L. (2021). *Sleep, behaviour and health-related quality of life in preschool-and school-aged children with cerebral palsy* ProQuest Information C Learning]. [https://search.ebscohost.com/login.aspx?direct=trueCdb=psyhCAN=2020-](https://search.ebscohost.com/login.aspx?direct=true&db=psyh&AN=2020-97495-223&site=ehost-live)

[97495-223Csite=ehost-live](https://search.ebscohost.com/login.aspx?direct=true&db=psyh&AN=2020-97495-223&site=ehost-live). Exclusion reason: Not peer-reviewed

Hosokawa, T., Kitahara, T., C Nakamura, R. (1985). Social skills of children with cerebral palsy. *Journal of Human Ergology*, *14*(2), 79-88.

[https://search.ebscohost.com/login.aspx?direct=trueCdb=psyhCAN=1987-](https://search.ebscohost.com/login.aspx?direct=true&db=psyh&AN=1987-01618-001&site=ehost-live) [01618-001Csite=ehost-live](https://search.ebscohost.com/login.aspx?direct=true&db=psyh&AN=1987-01618-001&site=ehost-live). Exclusion reason: 6-18 years

Houlihan, C. M., O'Donnell, M., Conaway, M., C Stevenson, R. D. (2004). Bodily pain and health-related quality of life in children with cerebral palsy. *Developmental Medicine & Child Neurology*, *4c*(5), 305-310.

<https://doi.org/10.1017/S0012162204000507>. Exclusion reason: 6-18 years Howe, G. W., Feinstein, C., Reiss, D., Molock, S., C Berger, K. (1993). Adolescent

adjustment to chronic physical disorders: I Comparing neurological and non- neurological conditions. *Child Psychology & Psychiatry & Allied Disciplines*, *34*(7), 1153-1171. <https://doi.org/10.1111/j.1469-7610.1993.tb01780.x>.

Exclusion reason: 6-18 years

Hsieh, H.-C. (2012). Effectiveness of adaptive pretend play on affective expression and imagination of children with cerebral palsy. *Research in Developmental*

*Disabilities*, *33*(6), 1975-1983. <https://doi.org/10.1016/j.ridd.2012.05.013>. Exclusion reason: 6-18 years

Indredavik, M. S., Vik, T., Heyerdahl, S., Romundstad, P., C Brubakk, A. M. (2005). Low- birthweight adolescents: quality of life and parent-child relations. *Acta Paediatr*, *S4*(9), 1295-1302. <https://doi.org/10.1111/j.1651-2227.2005.tb02091.x>.

Exclusion reason: 6-18 years

Jackson, K. E., Krishnaswami, S., C McPheeters, M. (2011). Unmet health care needs in children with cerebral palsy: A cross-sectional study. *Research in Developmental Disabilities*, *32*(6), 2714-2723.

<https://doi.org/10.1016/j.ridd.2011.05.040>. Exclusion reason: 6-18 years

James, S. D., C Egel, A. L. (1986). A direct prompting strategy for increasing reciprocal interactions between handicapped and nonhandicapped siblings. *Journal of*

*Applied Behavior Analysis*, *1S*(2), 173-186. [https://doi.org/10.1901/jaba.1986.19-](https://doi.org/10.1901/jaba.1986.19-173)

[173](https://doi.org/10.1901/jaba.1986.19-173). Exclusion reason: Mixed sample

Janssen, C. G. C., Voorman, J. M., Becher, J. G., Dallmeijer, A. J., C Schuengel, C. (2010).

Course of health-related quality of life in 9–16-year-old children with cerebral palsy: Associations with gross motor abilities and mental health. *Disability and Rehabilitation: An International, Multidisciplinary Journal*, *32*(4), 344-351. <https://doi.org/10.3109/09638280903166345>. Exclusion reason: 6-18 years

Jensen, M. P., Engel, J. M., C Schwartz, L. (2006). Coping with cerebral palsy pain: a preliminary longitudinal study. *Pain Med*, *7*(1), 30-37.

<https://doi.org/10.1111/j.1526-4637.2006.00086.x>. Exclusion reason: 6-18 years Kalizhniuk, E. S. (1972). [Delay in psychic development in children with cerebral palsy].

*Zh Nevropatol Psikhiatr Im S S Korsakova*, *72*(9), 1408-1412. Exclusion reason: Not able to locate study

Kalizhniuk, E. S. (1978). [Personality formation in younger school age children afflicted with cerebral paralysis]. *Zh Nevropatol Psikhiatr Im S S Korsakova*, *78*(10), 1554- 1558. Exclusion reason: 6-18 years

Kang, L. J., Palisano, R. J., King, G. A., Chiarello, L. A., Orlin, M. N., C Polansky, M. (2012).

Social participation of youths with cerebral palsy differed based on their self‐

perceived competence as a friend. *Child: Care, Health and Development*, *38*(1), 117-127. <https://doi.org/10.1111/j.1365-2214.2011.01222.x>. Exclusion reason: 6-18 years

King, G., King, S., Rosenbaum, P., C Goffin, R. (1999). Family-centered caregiving and well-being of parents of children with disabilities: Linking process with outcome. *Journal of Pediatric Psychology*, *24*(1), 41-53.

<https://doi.org/10.1093/jpepsy/24.1.41>. Exclusion reason: Mixed sample Kirichenko, E. I., C Trifonov, O. A. (1969). [Pathologic personality development in

children and adolescents with cerebral palsy]. *Zh Nevropatol Psikhiatr Im S S Korsakova*, *cS*(10), 1553-1556. Exclusion reason: Not able to locate study

Kirshner, S., Weiss, P. L., C Tirosh, E. (2016). Differences in autonomic functions as related to induced stress between children with and without cerebral palsy while performing a virtual meal-making task. *Research in Developmental Disabilities*, *4S-50*, 247-257. <https://doi.org/10.1016/j.ridd.2015.11.025>. Exclusion reason: 6-

18 years

Kohleis, K., Storck, M., Geissler-Preuss, S., Hirsch, A., Kuhn, F. D., Ortfeld, S., Rapp, M., C Bode, H. (2019). [Risk Factors for Mental Health Problems in Children with

Cerebral Palsy and Spina Bifida]. *Klin Padiatr*, *231*(1), 28-34. <https://doi.org/10.1055/a-0664-0832>. Exclusion reason: 6-18 years

Kohler, C., C Lachanat, J. (1972). The 'corporal schema' of children with cerebromotor disability. *Annales Médico-Psychologiques*, *2*(2), 177-187.

[https://search.ebscohost.com/login.aspx?direct=trueCdb=psyhCAN=1973-](https://search.ebscohost.com/login.aspx?direct=true&db=psyh&AN=1973-25098-001&site=ehost-live) [25098-001Csite=ehost-live](https://search.ebscohost.com/login.aspx?direct=true&db=psyh&AN=1973-25098-001&site=ehost-live). Exclusion reason: Outcome not socioemotional

Kok, S. E., van der Burg, J. J., van Hulst, K., Erasmus, C. E., C van den Hoogen, F. J. (2016). The impact of submandibular duct relocation on drooling and the well-being of children with neurodevelopmental disabilities. *Int J Pediatr Otorhinolaryngol*, *88*, 173-178.

<https://doi.org/10.1016/j.ijporl.2016.06.043>. Exclusion reason: Mixed sample

Kolman, S. E., Glanzman, A. M., Prosser, L., Spiegel, D. A., C Baldwin, K. D. (2018). Factors that Predict Overall Health and Quality of Life in Non-Ambulatory Individuals with Cerebral Palsy. *The Iowa orthopaedic journal*, *38*, 147-152. Exclusion reason: Outcome not socioemotional

Krahn, G., C Havercamp, S. (2019). Shining the Light on Mental Health in a Population at Risk: Cerebral Palsy and Other Developmental Disabilities. *Ann Intern Med*,

*171*(5), 370-371. <https://doi.org/10.7326/M19-1951>. Exclusion reason: Not original study

Kramer, L. A. (1999). *Effects of social competence factors on self-esteem and behavior in adolescents with cerebral palsy* ProQuest Information C Learning]. [https://search.ebscohost.com/login.aspx?direct=trueCdb=psyhCAN=1999-](https://search.ebscohost.com/login.aspx?direct=true&db=psyh&AN=1999-95003-094&site=ehost-live)

[95003-094Csite=ehost-live](https://search.ebscohost.com/login.aspx?direct=true&db=psyh&AN=1999-95003-094&site=ehost-live). Exclusion reason: Not peer reviewed

Lalkhen, Y. (2002). *A phenomenological understanding of self-esteem in physically disabled adolescents in a non-disabled environment* ProQuest Information C Learning].

[https://search.ebscohost.com/login.aspx?direct=trueCdb=psyhCAN=2002-](https://search.ebscohost.com/login.aspx?direct=true&db=psyh&AN=2002-95008-331&site=ehost-live) [95008-331Csite=ehost-live](https://search.ebscohost.com/login.aspx?direct=true&db=psyh&AN=2002-95008-331&site=ehost-live). Exclusion reason: Not peer reviewed

Langher, V., Kourkoutas, E., Scurci, G., C Tolve, G. (2010). *Perception of the security of attachment in neurologically ill children.* Exclusion reason: 6-18 years

Langher, V., Scurci, G., Tolve, G., C Caputo, A. (2013). Perception of attachment security in families with children affected by neurological illness. *PSIHOLOGIJA*, *4c*(2),

99-110. <https://doi.org/10.2298/PSI1302099L>. Exclusion reason: Mixed sample Laporta-Hoyos, O., Ballester-Plané, J., Póo, P., Macaya, A., Meléndez-Plumed, M.,

Vázquez, E., Delgado, I., Zubiaurre-Elorza, L., Botellero, V. L., Narberhaus, A., Toro-Tamargo, E., Segarra, D., C Pueyo, R. (2017). Proxy-reported quality of life in adolescents and adults with dyskinetic cerebral palsy is associated with

executive functions and cortical thickness. *Ǫuality of Life Research: An International Journal of Ǫuality of Life Aspects of Treatment, Care &*

*Rehabilitation*, *2c*(5), 1209-1222. <https://doi.org/10.1007/s11136-016-1433-0>.

Exclusion reason: 6-18 years

Law, M., Hanna, S., Anaby, D., Kertoy, M., King, G., C Xu, L. (2014). Health-related quality of life of children with physical disabilities: a longitudinal study. *BMC pediatrics*, *14*(1), 1-10. Exclusion reason: 6-18 years

Leader, G., Molina Bonilla, P., Naughton, K., Maher, L., Casburn, M., Arndt, S., C Mannion, A. (2021). Complex comorbid presentations are associated with

harmful behavior problems among children and adolescents with cerebral palsy.

*Developmental Neurorehabilitation*, *24*(1), 25-34.

<https://doi.org/10.1080/17518423.2020.1770353>. Exclusion reason: 6-18 years Leader, G., Mooney, A., Chen, J. L., Whelan, S., Naughton, K., Maher, L., C Mannion, A. (2022). The co-occurrence of autism spectrum disorder and cerebral palsy and

associated comorbid conditions in children and adolescents. *Developmental Neurorehabilitation*, *25*(5), 289-297. Exclusion reason: 6-18 years

<https://doi.org/10.1080/17518423.2021.2011456>

Lee, S., C Kahn, J. V. (2000). A survival analysis of parent-child interaction in early intervention. *Infant-Toddler Intervention*, *10*(3), 137-156.

[https://search.ebscohost.com/login.aspx?direct=trueCdb=psyhCAN=2000-](https://search.ebscohost.com/login.aspx?direct=true&db=psyh&AN=2000-02556-002&site=ehost-live) [02556-002Csite=ehost-live](https://search.ebscohost.com/login.aspx?direct=true&db=psyh&AN=2000-02556-002&site=ehost-live). Exclusion reason: Not able to locate study

Leland, H., C Smith, D. (1962). Unstructured material in play therapy for emotionally

disturbed, brain damaged mentally retarded children. *Am J Ment Defic*, *cc*, 621- 628. Exclusion reason: Outcome not socioemotional

Lempp, R. (1969). Cerebral lesion of early childhood and later neurotic development.

*Praxis der Psychotherapie*, *14*(6), 274-281.

[https://search.ebscohost.com/login.aspx?direct=trueCdb=psyhCAN=1971-](https://search.ebscohost.com/login.aspx?direct=true&db=psyh&AN=1971-25332-001&site=ehost-live) [25332-001Csite=ehost-live](https://search.ebscohost.com/login.aspx?direct=true&db=psyh&AN=1971-25332-001&site=ehost-live). Exclusion reason: Not original study

Levy-Zaks, A., Pollak, Y., C Ben-Pazi, H. (2014). Cerebral palsy risk factors and their impact on psychopathology. *Neurol Res*, *3c*(1), 92-94.

<https://doi.org/10.1179/1743132813Y.0000000290>. Exclusion reason: 6-18 years Lewis, V. (1987). *Development and handicap*. Basil Blackwell.

[https://search.ebscohost.com/login.aspx?direct=trueCdb=psyhCAN=1987-](https://search.ebscohost.com/login.aspx?direct=true&db=psyh&AN=1987-98592-000&site=ehost-live) [98592-000Csite=ehost-live](https://search.ebscohost.com/login.aspx?direct=true&db=psyh&AN=1987-98592-000&site=ehost-live). Exclusion reason: Not peer reviewed

Lewis, V. (2003). *Development and disability, 2nd ed*. Blackwell Publishing. [https://search.ebscohost.com/login.aspx?direct=trueCdb=psyhCAN=2002-](https://search.ebscohost.com/login.aspx?direct=true&db=psyh&AN=2002-18571-000&site=ehost-live) [18571-000Csite=ehost-live](https://search.ebscohost.com/login.aspx?direct=true&db=psyh&AN=2002-18571-000&site=ehost-live). Exclusion reason: Not peer reviewed

Li, X., Wang, K., Wu, J., Hong, Y., Zhao, J., Feng, X., Xu, M., Wang, M., Ndasauka, Y., C Zhang, X. (2014). The link between impaired theory of mind and executive

function in children with cerebral palsy. *Research in Developmental Disabilities*, *35*(7), 1686-1693. <https://doi.org/10.1016/j.ridd.2014.03.017>. Exclusion reason:

6-18 years

Lin, S.-L. (2000). Coping and adaptation in families of children with cerebral palsy.

*Exceptional Children*, *cc*(2), 201-218.

[https://search.ebscohost.com/login.aspx?direct=trueCdb=psyhCAN=2000-](https://search.ebscohost.com/login.aspx?direct=true&db=psyh&AN=2000-15829-005&site=ehost-live) [15829-005Csite=ehost-live](https://search.ebscohost.com/login.aspx?direct=true&db=psyh&AN=2000-15829-005&site=ehost-live). Exclusion reason: 6-18 years

Livingston, M. H., C Rosenbaum, P. L. (2008). Adolescents with cerebral palsy: Stability in measurement of quality of life and health-related quality of life over 1 year.

*Developmental Medicine & Child Neurology*, *50*(9), 696-701.

<https://doi.org/10.1111/j.1469-8749.2008.03053.x>. Exclusion reason: 6-18 years Longo, E., Badia, M., Begoña Orgaz, M., C Gómez-Vela, M. (2017). Comparing parent

and child reports of health-related quality of life and their relationship with

leisure participation in children and adolescents with cerebral palsy. *Research in Developmental Disabilities*, *71*, 214-222.

<https://doi.org/10.1016/j.ridd.2017.09.020>. Exclusion reason: 6-18 years Longo, E., Badia, M., C Orgaz, B. M. (2013). Patterns and predictors of participation in

leisure activities outside of school in children and adolescents with Cerebral Palsy. *Research in Developmental Disabilities*, *34*(1), 266-275.

<https://doi.org/10.1016/j.ridd.2012.08.017>. Exclusion reason: 6-18 years

Love, N. W., Jr. (1970). The relative occurrence of secondary disabilities in children with cerebral palsy and other primary physical handicaps. *Except Child*, *37*(4), 301-

302. Exclusion reason: 6-18 years

Lust, M. (1964). [THE PSYCHIC DISORDERS AND RETARDATION IN CHILDREN WITH

PSYCHO-CINETIC DISORDERS RESULTING FROM A CHRONIC, NON EVOLUTIVE ENCEPHALOPATHY (CEREBRAL PALSY)]. *Clin Pediatr (Bologna)*, *4c*, 577-582.

Exclusion reason: Not able to locate study

Maher, C. A., Toohey, M., C Ferguson, M. (2016). Physical activity predicts quality of life and happiness in children and adolescents with cerebral palsy. *Disability and*

*Rehabilitation: An International, Multidisciplinary Journal*, *38*(9), 865-869. <https://doi.org/10.3109/09638288.2015.1066450>. Exclusion reason: 6-18 years

Majnemer, A., Shevell, M., Hall, N., Poulin, C., C Law, M. (2010). Developmental and

functional abilities in children with cerebral palsy as related to pattern and level of motor function. *Journal of Child Neurology*, *25*(10), 1236-1241.

<https://doi.org/10.1177/0883073810363175>. Exclusion reason: 6-18 years

Majnemer, A., Shevell, M., Law, M., Poulin, C., C Rosenbaum, P. (2008). Reliability in the ratings of quality of life between parents and their children of school age with cerebral palsy. *Ǫuality of Life Research: An International Journal of Ǫuality of Life Aspects of Treatment, Care & Rehabilitation*, *17*(9), 1163-1171.

<https://doi.org/10.1007/s11136-008-9394-6>. Exclusion reason: 6-18 years

Majnemer, A., Shevell, M., Law, M., Poulin, C., C Rosenbaum, P. (2012). Indicators of

distress in families of children with cerebral palsy. *Disabil Rehabil*, *34*(14), 1202- 1207. <https://doi.org/10.3109/09638288.2011.638035>. Exclusion reason: 6-18 years

Majnemer, A., Shevell, M., Rosenbaum, P., Law, M., C Poulin, C. (2007). Determinants of life quality in school-age children with cerebral palsy. *The Journal of Pediatrics*, *151*(5), 470-475. <https://doi.org/10.1016/j.jpeds.2007.04.014>. Exclusion reason:

6-18 years

Majnemer, A., Shikako-Thomas, K., Lach, L., Shevell, M., Law, M., C Schmitz, N. (2013).

Mastery motivation in adolescents with cerebral palsy. *Research in Developmental Disabilities*, *34*(10), 3384-3392.

<https://doi.org/10.1016/j.ridd.2013.07.002>. Exclusion reason: 6-18 years Mamaychyuk, I. I., C Pyatakova, G. V. (1990). Investigation of personality peculiarities in

cerebral palsied children. *Defektologiya*, *3*, 23-28.

[https://search.ebscohost.com/login.aspx?direct=trueCdb=psyhCAN=1991-](https://search.ebscohost.com/login.aspx?direct=true&db=psyh&AN=1991-73772-001&site=ehost-live) [73772-001Csite=ehost-live](https://search.ebscohost.com/login.aspx?direct=true&db=psyh&AN=1991-73772-001&site=ehost-live). Exclusion reason: 6-18 years

Marret, S., Marchand, L., Kaminski, M., Larroque, B., Arnaud, C., Truffert, P., Thirez, G.,

Fresson, J., Rozé, J.-C., C Ancel, P.-Y. (2010). Prenatal low-dose aspirin and

neurobehavioral outcomes of children born very preterm. *Pediatrics*, *125*(1), e29- e34. <https://doi.org/10.1542/peds.2009-0994>. Exclusion reason: Population not CP

McCormick, M. C., McCarton, C., Tonascia, J., C Brooks-Gunn, J. (1993). Early

educational intervention for very low birth weight infants: results from the Infant Health and Development Program. *J Pediatr*, *123*(4), 527-533.

<https://doi.org/10.1016/s0022-3476(05)80945-x>. Exclusion reason: Population not CP

McDermott, S., Coker, A. L., Mani, S., Krishnaswami, S., Nagle, R. J., Barnett-Queen, L. L., C Wuori, D. F. (1996). A population-based analysis of behavior problems in children with cerebral palsy. *Journal of Pediatric Psychology*, *21*(3), 447-463. <https://doi.org/10.1093/jpepsy/21.3.447>. Exclusion reason: 6-18 years

McDermott, S., Nagle, R., Wright, H. H., Swann, S., Leonhardt, T., C Wuori, D. (2002).

Consultation in paediatric rehabilitation for behaviour problems in young children with cerebral palsy and/or developmental delay. *Pediatr Rehabil*, *5*(2),

99-106. <https://doi.org/10.1080/1363849021000013531>. Exclusion reason: Mixed sample

McFadd, E. (2018). *Mother-child interaction in children with cerebral palsy* ProQuest Information C Learning].

[https://search.ebscohost.com/login.aspx?direct=trueCdb=psyhCAN=2018-](https://search.ebscohost.com/login.aspx?direct=true&db=psyh&AN=2018-00726-167&site=ehost-live) [00726-167Csite=ehost-live](https://search.ebscohost.com/login.aspx?direct=true&db=psyh&AN=2018-00726-167&site=ehost-live). Exclusion reason: Not peer reviewed

McIntyre, S. (2020). Think beyond movement C posture; mental disorders in cerebral palsy. *Eur J Paediatr Neurol*, *27*, 8. <https://doi.org/10.1016/j.ejpn.2020.06.012>. Exclusion reason: Not original study

McMahon, J., Harvey, A., Reid, S. M., May, T., C Antolovich, G. (2020). Anxiety in children and adolescents with cerebral palsy. *J Paediatr Child Health*, *5c*(8), 1194-1200. <https://doi.org/10.1111/jpc.14879>. Exclusion reason: 6-18 years

Merrick, H., McConachie, H., Le Couteur, A., Mann, K., Parr, J. R., Pearce, M. S., C Colver, A. (2015). Characteristics of young people with long term conditions close to transfer to adult health services. *BMC Health Serv Res*, *15*, 435. <https://doi.org/10.1186/s12913-015-1095-6>. Exclusion reason: 6-18 years

Mezgebe, M., Akhtar-Danesh, G.-G., Streiner, D. L., Fayed, N., Rosenbaum, P. L., C Ronen, G. M. (2015). Quality of life in children with epilepsy: How does it compare with the quality of life in typical children and children with cerebral palsy? *Epilepsy & Behavior*, *52*(Part A), 239-243.

<https://doi.org/10.1016/j.yebeh.2015.09.009>. Exclusion reason: 6-18 years

Michalska, A., Markowska, M., Śliwiński, Z., C Pogorzelska, J. A. (2018). Quality of life in children and young people with tetraplegic cerebral palsy. *Studia medyczne*,

*34*(2), 112-119. <https://doi.org/10.5114/ms.2018.76871>. Exclusion reason: 6-18 years

Miller, A. C., Johann-Murphy, M., C Cate, I. M. P.-t. (1997). Pain, anxiety, and

cooperativeness in children with cerebral palsy after rhizotomy: Changes throughout rehabilitation. *Journal of Pediatric Psychology*, *22*(5), 689-705. <https://doi.org/10.1093/jpepsy/22.5.689>. Exclusion reason: 6-18 years

Miller, C. J. (1976). Children with feeding problems. *Child: Care, Health and Development*, *2*(2), 73-76. <https://doi.org/10.1111/j.1365-2214.1976.tb00860.x>. Exclusion reason: Outcome not socioemotional

Miller, E., C Rosenfeld, G. B. (1952). Psychological evaluation of children with cerebral palsy and its implications in treatment. *AMA Am J Dis Child*, *84*(4), 504-505.

Exclusion reason: Outcome not socioemotional

Molnar, G. E. (1989). The influence of psychosocial factors on personality development and emotional health in children with cerebral palsy and spina bifida. In (pp. 87- 107). Rutgers University Press.

[https://search.ebscohost.com/login.aspx?direct=trueCdb=psyhCAN=1989-](https://search.ebscohost.com/login.aspx?direct=true&db=psyh&AN=1989-98670-005&site=ehost-live) [98670-005Csite=ehost-live](https://search.ebscohost.com/login.aspx?direct=true&db=psyh&AN=1989-98670-005&site=ehost-live). Exclusion reason: Not peer reviewed

Moreira, H., Carona, C., Silva, N., Frontini, R., Bullinger, M., C Canavarro, M. C. (2013). Psychological and quality of life outcomes in pediatric populations: A parent- child perspective. *The Journal of Pediatrics*, *1c3*(5), 1471-1478.

<https://doi.org/10.1016/j.jpeds.2013.06.028>. Exclusion reason: 6-18 years Morog, M. C. (1997). *Trauma and its relation to working models of relationships:*

*Attachment and loss in mothers of children with disabilities* ProQuest Information C

Learning].[https://search.ebscohost.com/login.aspx?direct=trueCdb=psyhCAN=](https://search.ebscohost.com/login.aspx?direct=true&db=psyh&AN=1997-95004-172&site=ehost-live) [1997-95004-172Csite=ehost-live](https://search.ebscohost.com/login.aspx?direct=true&db=psyh&AN=1997-95004-172&site=ehost-live). Exclusion reason: Not peer reviewed

Mullen, S. W. (1998). *The impact of child disability on marriage, parenting, and*

*attachment: Relationships in families with a child with cerebral palsy* ProQuest Information C Learning].

https://search.ebscohost.com/login.aspx?direct=trueCdb=psyhCAN=1998- 95002-049Csite=ehost-liv.e. Exclusion reason: Not peer reviewed

Munir, K. M., C Rustamov, I. (2019). Mental health in children and adolescents with cerebral palsy. In (pp. 211-225). Mac Keith Press.

[https://search.ebscohost.com/login.aspx?direct=trueCdb=psyhCAN=2018-](https://search.ebscohost.com/login.aspx?direct=true&db=psyh&AN=2018-31846-013&site=ehost-live) [31846-013Csite=ehost-live](https://search.ebscohost.com/login.aspx?direct=true&db=psyh&AN=2018-31846-013&site=ehost-live). Exclusion reason: Not peer reviewed

Murphy, N., Caplin, D. A., Christian, B. J., Luther, B. L., Holobkov, R., C Young, P. C.

(2011). The function of parents and their children with cerebral palsy. *PM R*, *3*(2), 98-104. <https://doi.org/10.1016/j.pmrj.2010.11.006>. Exclusion reason: 6-18 years

Nadeau, L., C Tessier, R. (2006). Social adjustment of children with cerebral palsy in mainstream classes: Peer perception. *Developmental Medicine & Child Neurology*, *48*(5), 331-336. <https://doi.org/10.1017/S0012162206000739>.

Exclusion reason: 6-18 years

Nagle, R. J., C Campbell, L. H. (1998). Cerebral palsy. In (pp. 145-153). American Psychological Association. <https://doi.org/10.1037/10300-020>. Exclusion reason: Not peer reviewed.

Nassau, J. H., C Drotar, D. (1997). Social competence among children with central

nervous system-related chronic health conditions: A review. *Journal of Pediatric Psychology*, *22*(6), 771-793. <https://doi.org/10.1093/jpepsy/22.6.771>. Exclusion reason: Mixed sample

Nielsen, H. H. (1964). Social vulnerability C adjustment of cerebral palsied children.

*Scandinavian Journal of Psychology*, *5*(1), 26-32. [https://doi.org/10.1111/j.1467-](https://doi.org/10.1111/j.1467-9450.1964.tb01405.x) [9450.1964.tb01405.x](https://doi.org/10.1111/j.1467-9450.1964.tb01405.x). Exclusion reason: 6-18 years

Nussbaum, J. (1966). Self-concept of adolescents with cerebral palsy. *Cerebral Palsy Journal*, *27*(4), 5-7.

[https://search.ebscohost.com/login.aspx?direct=trueCdb=psyhCAN=1967-](https://search.ebscohost.com/login.aspx?direct=true&db=psyh&AN=1967-01819-001&site=ehost-live) [01819-001Csite=ehost-live](https://search.ebscohost.com/login.aspx?direct=true&db=psyh&AN=1967-01819-001&site=ehost-live). Exclusion reason: Not able to locate study

Okurowska-Zawada, B., Kułak, W., Otapowicz, D., Sienkiewicz, D., Paszko-Patej, G., C Wojtkowski, J. (2011). Quality of life in children and adolescents with cerebral palsy and myelomeningocele. *Pediatr Neurol*, *45*(3), 163-168.

<https://doi.org/10.1016/j.pediatrneurol.2011.04.006>. Exclusion reason: 6-18 years

Ong, L. C., Boo, N. Y., C Chandran, V. (2001). Predictors of neurodevelopmental

outcome of Malaysian very low birthweight children at 4 years of age. *Journal of Paediatrics and Child Health*, *37*(4), 363-368. [https://doi.org/10.1046/j.1440-](https://doi.org/10.1046/j.1440-1754.2001.00694.x) [1754.2001.00694.x](https://doi.org/10.1046/j.1440-1754.2001.00694.x). Exclusion reason: Population not CP

Padilla, A. (1960). [Treatment of behavior disorders and epilepsy in infantile cerebral paralysis]. *Rev Chil Pediatr*, *31*, 467-471. Exclusion reason: Not able to locate study

Padilla, A. (1961). [Treatment of behavior disorders and epilepsy in cerebral paralysis].

*Neurocirugia*, *1S*, 77-80. Exclusion reason: Not able to locate study

Parette, H. P., C Hourcade, J. J. (1984). The student with cerebral palsy and the public schools: Implications for the counselor. *Elementary School Guidance &*

*Counseling*, *1S*(2), 141-146.

[https://search.ebscohost.com/login.aspx?direct=trueCdb=psyhCAN=1985-](https://search.ebscohost.com/login.aspx?direct=true&db=psyh&AN=1985-15957-001&site=ehost-live) [15957-001Csite=ehost-live](https://search.ebscohost.com/login.aspx?direct=true&db=psyh&AN=1985-15957-001&site=ehost-live). Exclusion reason: Not original study

Park, E.-Y. (2017). Relationship between activity limitation and health-related quality of life in school-aged children with cerebral palsy: A cross-sectional study. *Health and Ǫuality of Life Outcomes*, *15*.

[https://search.ebscohost.com/login.aspx?direct=trueCdb=psyhCAN=2017-](https://search.ebscohost.com/login.aspx?direct=true&db=psyh&AN=2017-19661-001&site=ehost-liveeungyoung%40jj.ac.kr)

[19661-001Csite=ehost-liveeungyoung@jj.ac.kr](https://search.ebscohost.com/login.aspx?direct=true&db=psyh&AN=2017-19661-001&site=ehost-liveeungyoung%40jj.ac.kr). Exclusion reason: Outcome not socioemotional

Park, E.-Y. (2018). Path analysis of strength, spasticity, gross motor function, and health- related quality of life in children with spastic cerebral palsy. *Health and Ǫuality of Life Outcomes*, *1c*.

[https://search.ebscohost.com/login.aspx?direct=trueCdb=psyhCAN=2018-](https://search.ebscohost.com/login.aspx?direct=true&db=psyh&AN=2018-18167-001&site=ehost-liveeungyoung%40jj.ac.kr)

[18167-001Csite=ehost-liveeungyoung@jj.ac.kr](https://search.ebscohost.com/login.aspx?direct=true&db=psyh&AN=2018-18167-001&site=ehost-liveeungyoung%40jj.ac.kr). Exclusion reason: Outcome not socioemotional

Parkes, J., McCullough, N., Madden, A., C McCahey, E. (2009). The health of children with cerebral palsy and stress in their parents. *Journal of Advanced Nursing*,

*c5*(11), 2311-2323. <https://doi.org/10.1111/j.1365-2648.2009.05089.x>. Exclusion reason: 6-18 years

Parkes, J., White-Koning, M., Dickinson, H. O., Thyen, U., Arnaud, C., Beckung, E., Fauconnier, J., Marcelli, M., McManus, V., Michelsen, S. I., Parkinson, K., C Colver, A. (2008). Psychological problems in children with cerebral palsy: A cross-sectional European study. *Journal of Child Psychology and Psychiatry*,

*4S*(4), 405-413. <https://doi.org/10.1111/j.1469-7610.2007.01845.x>. Exclusion reason: 6-18 years

Parkinson, K. N., Rice, H., C Young, B. (2011). Incorporating children's and their parents' perspectives into condition-specific quality-of-life instruments for children with cerebral palsy: a qualitative study. *Value Health*, *14*(5), 705-711.

<https://doi.org/10.1016/j.jval.2010.12.003>. Exclusion reason: 6-18 years

Paulus, F. W. (2019). A bio-psycho-social view of cerebral palsy: friendships reduce mental health disorders. *Dev Med Child Neurol*, *c1*(8), 862.

<https://doi.org/10.1111/dmcn.14200>. Exclusion reason: Not original study Pennington, L., C McConachie, H. (1999). Mother-child interaction revisited:

communication with non-speaking physically disabled children. *Int J Lang*

*Commun Disord*, *34*(4), 391-416. <https://doi.org/10.1080/136828299247351>. Exclusion reason: Outcome not socioemotional

Peterman, J. S., Hoff, A. L., Gosch, E., C Kendall, P. C. (2015). Cognitive-behavioral therapy for anxious youth with a physical disability: A case study. *Clinical Case Studies*, *14*(3), 210-226. <https://doi.org/10.1177/1534650114552556>. Exclusion

reason: 6-18 years

Peters, P. A., Huisman, J., C van Emmerik-Levelt, H. M. (1999). Gedragsproblemen en emotionele problemen bij kinderen in het Mytyl- en Tyltylonderwijs = Behavioral and emotional problems in children at schools for physically handicapped children and for multiply handicapped children. *Kind en Adolescent*, *20*(1), 44-

51. <https://doi.org/10.1007/BF03060723>. Exclusion reason: 6-18 years

Petersen, C., Schmidt, S., Power, M., C Bullinger, M. (2005). Development and pilot-

testing of a health-related quality of life chronic generic module for children and adolescents with chronic health conditions: A European perspective. *Ǫuality of Life Research: An International Journal of Ǫuality of Life Aspects of Treatment, Care & Rehabilitation*, *14*(4), 1065-1077. [https://doi.org/10.1007/s11136-004-](https://doi.org/10.1007/s11136-004-2575-z)

[2575-z](https://doi.org/10.1007/s11136-004-2575-z). Exclusion reason: 6-18 years

Petersen, M. C., Kube, D. A., C Palmer, F. B. (1998). Classification of developmental delays. *Semin Pediatr Neurol*, *5*(1), 2-14. [https://doi.org/10.1016/s1071-](https://doi.org/10.1016/s1071-9091(98)80012-0) [9091(98)80012-0](https://doi.org/10.1016/s1071-9091(98)80012-0). Exclusion reason: Outcome not socioemotional

Phelps, W. M. (1948). Characteristic psychological variations in cerebral palsy. *Nerv Child*, *7*(1), 10-13. Exclusion reason: Not original study

Pianta, R. C., Marvin, R. S., C Morog, M. C. (1999). Resolving the past and present: Relations with attachment organization. In (pp. 379-398). The Guilford Press. [https://search.ebscohost.com/login.aspx?direct=trueCdb=psyhCAN=1999-](https://search.ebscohost.com/login.aspx?direct=true&db=psyh&AN=1999-04187-014&site=ehost-live) [04187-014Csite=ehost-live](https://search.ebscohost.com/login.aspx?direct=true&db=psyh&AN=1999-04187-014&site=ehost-live). Exclusion reason: Not peer reviewed

Pierrat, V., Marchand-Martin, L., Arnaud, C., Kaminski, M., Resche-Rigon, M., Lebeaux, C., Bodeau-Livinec, F., Morgan, A. S., Goffinet, F., Marret, S., C Ancel, P. Y. (2017). Neurodevelopmental outcome at 2 years for preterm children born at 22 to 34 weeks' gestation in France in 2011: EPIPAGE-2 cohort study. *BMJ*, *358*, j3448. <https://doi.org/10.1136/bmj.j3448>. Exclusion reason: Population not CP

Pinquart, M. (2017). [Psychological Health of Children with Chronic Physical Illness and their Parents - Results from Meta-Analyses]. *Prax Kinderpsychol Kinderpsychiatr*, *cc*(9), 656-671. <https://doi.org/10.13109/prkk.2017.66.9.656>. Exclusion reason: Not original study

Pirpiris, M., Gates, P. E., McCarthy, J. J., D'Astous, J., Tylkowksi, C., Sanders, J. O., Dorey,

F. J., Ostendorff, S., Robles, G., C Caron, C. (2006). Function and well-being in ambulatory children with cerebral palsy. *Journal of Pediatric Orthopaedics*, *2c*(1), 119-124. Exclusion reason: 6-18 years

Pivry, S., Bréchon, G., C Scelles, R. (2020). Processus dépressif chez l’adolescent déficient moteur avec troubles cognitifs associés, accueilli en Institut

d’Éducation Motrice: Étude qualitative et prospective = Depressive process in young people with physical disability and cognitive disorders, in.

*Neuropsychiatrie de l'Enfance et de l'Adolescence*, *c8*(2), 83-92.

<https://doi.org/10.1016/j.neurenf.2019.11.005>. Exclusion reason: 6-18 years Polacco, M. (1997). Hypothesis about the thought in children with cerebral palsy.

*Giornale di Neuropsichiatria dell'Età Evolutiva*, *17*(2), 87-96.

[https://search.ebscohost.com/login.aspx?direct=trueCdb=psyhCAN=1997-](https://search.ebscohost.com/login.aspx?direct=true&db=psyh&AN=1997-06982-001&site=ehost-live) [06982-001Csite=ehost-live](https://search.ebscohost.com/login.aspx?direct=true&db=psyh&AN=1997-06982-001&site=ehost-live). Exclusion reason: Not original study

Pond, D. A. (1961). Psychiatric aspects of epileptic and brain-damaged children. *Br Med J*, *2*(5265), 1454-1459. <https://doi.org/10.1136/bmj.2.5265.1454>. Exclusion reason: Mixed sample

Power, R., Akhter, R., Muhit, M., Wadud, S., Heanoy, E., Karim, T., Badawi, N., C

Khandaker, G. (2019). A quality of life questionnaire for adolescents with cerebral palsy: Psychometric properties of the Bengali CPQoL-teens. *Health and Ǫuality of Life Outcomes*, *17*.

https://search.ebscohost.com/login.aspx?direct=trueCdb=psyhCAN=2019- 46037-001Csite=ehost-liveORCID: 0000-0002-0661-4113ORCID: 0000-0003-

1480-8409ORCID: 0000-0002-6313-4343ORCID: 0000-0001-9723-7343ORCID:

[0000-0001-9434-3941gulam.khandaker@health.nsw.gov.a.](mailto:0000-0001-9434-3941gulam.khandaker@health.nsw.gov.a) Exclusion reason: 6- 18 years

Power, R., Galea, C., Muhit, M., Heanoy, E., Karim, T., Badawi, N., C Khandaker, G. (2020). What predicts the proxy-reported health-related quality of life of

adolescents with cerebral palsy in Bangladesh? *BMC Public Health*, *20*(1), 18. <https://doi.org/10.1186/s12889-019-8130-1>. Exclusion reason: 6-18 years

Power, R., Muhit, M., Heanoy, E., Karim, T., Badawi, N., Akhter, R., C Khandaker, G. (2019). Health-related quality of life and mental health of adolescents with cerebral palsy in rural Bangladesh. *PLoS ONE*, *14*(6).

<https://doi.org/10.1371/journal.pone.0217675>. Exclusion reason: 6-18 years Rackauskaite, G., Bilenberg, N., Bech, B. H., Uldall, P., C Østergaard, J. R. (2016).

Screening for psychopathology in a national cohort of 8- to 15-year-old children with cerebral palsy. *Research in Developmental Disabilities*, *4S-50*, 171-180. <https://doi.org/10.1016/j.ridd.2015.11.019>. Exclusion reason: 6-18 years

Rackauskaite, G., Bilenberg, N., Uldall, P., Bech, B. H., C Østergaard, J. (2020).

Prevalence of mental disorders in children and adolescents with cerebral palsy: Danish nationwide follow-up study. *Eur J Paediatr Neurol*, *27*, 98-103.

<https://doi.org/10.1016/j.ejpn.2020.03.004>. Exclusion reason: 6-18 years

Ramstad, K., Jahnsen, R., Skjeldal, O. H., C Diseth, T. H. (2012a). Mental health, health related quality of life and recurrent musculoskeletal pain in children with cerebral palsy 8–18 years old. *Disability and Rehabilitation: An International, Multidisciplinary Journal*, *34*(19), 1589-1595.

<https://doi.org/10.3109/09638288.2012.656794>. Exclusion reason: 6-18 years Ramstad, K., Jahnsen, R., Skjeldal, O. H., C Diseth, T. H. (2012b). Parent-reported

participation in children with cerebral palsy: The contribution of recurrent musculoskeletal pain and child mental health problems. *Developmental Medicine & Child Neurology*, *54*(9), 829-835. [https://doi.org/10.1111/j.1469-](https://doi.org/10.1111/j.1469-8749.2012.04341.x) [8749.2012.04341.x](https://doi.org/10.1111/j.1469-8749.2012.04341.x). Exclusion reason: 6-18 years

Ramstad, K., Loge, J. H., Jahnsen, R., C Diseth, T. H. (2015). Self-reported mental health in youth with cerebral palsy and associations to recurrent musculoskeletal pain. *Disability and Rehabilitation: An International, Multidisciplinary Journal*, *37*(2),

144-150. <https://doi.org/10.3109/09638288.2014.913703>. Exclusion reason: 6-18 years

Rapp, M., Eisemann, N., Arnaud, C., Ehlinger, V., Fauconnier, J., Marcelli, M., Michelsen,

S. I., Nystrand, M., Colver, A., C Thyen, U. (2017). Predictors of parent-reported quality of life of adolescents with cerebral palsy: A longitudinal study. *Research in Developmental Disabilities*, *c2*, 259-270.

<https://doi.org/10.1016/j.ridd.2016.12.005>. Exclusion reason: 6-18 years

Resnick, M. D., C Hutton, L. (1987). Resiliency among physically disabled adolescents.

*Psychiatric Annals*, *17*(12), 796-800. [https://doi.org/10.3928/0048-5713-](https://doi.org/10.3928/0048-5713-19871201-09)

[19871201-09](https://doi.org/10.3928/0048-5713-19871201-09). Exclusion reason: 6-18 years

Romeo, D. M., Cioni, M., Distefano, A., Battaglia, L. R., Costanzo, L., Ricci, D., De Sanctis, R., Romeo, M. G., Mazzone, D., C Mercuri, E. (2010). Quality of life in

parents of children with cerebral palsy: is it influenced by the child's behaviour?

*Neuropediatrics*, *41*(3), 121-126. <https://doi.org/10.1055/s-0030-1262841>. Exclusion reason: 6-18 years

Roux, G., Sofronoff, K., C Sanders, M. (2013). A randomized controlled trial of group Stepping Stones Triple P: a mixed-disability trial. *Fam Process*, *52*(3), 411-424. <https://doi.org/10.1111/famp.12016>. Exclusion reason: Mixed sample

Ruisel, I., C Arochová, O. (1979). [Psychological aspects of resistance to stress in children with infantile cerebral palsies]. *Cesk Neurol Neurochir*, *42*(1), 18-23. Exclusion reason: Not able to locate study

Russo, R. N., Goodwin, E. J., Miller, M. D., Haan, E. A., Connell, T. M., C Crotty, M. (2008). Self-esteem, self-concept, and quality of life in children with hemiplegic cerebral palsy. *The Journal of Pediatrics*, *153*(4), 473-477.

<https://doi.org/10.1016/j.jpeds.2008.05.040>. Exclusion reason: 6-18 years Russo, R. N., Miller, M. D., Haan, E., Cameron, I. D., C Crotty, M. (2008). Pain

characteristics and their association with quality of life and self-concept in children with hemiplegic cerebral palsy identified from a population register. *The Clinical journal of pain*, *24*(4), 335-342. Exclusion reason: 6-18 years

Rutter, M. (1977). Brain damage syndromes in childhood: Concepts and findings. *Child Psychology & Psychiatry & Allied Disciplines*, *18*(1), 1-21.

<https://doi.org/10.1111/j.1469-7610.1977.tb00413.x>. Exclusion reason: Not original study

Sakzewski, L., Carlon, S., Shields, N., Ziviani, J., Ware, R. S., C Boyd, R. N. (2012). Impact of intensive upper limb rehabilitation on quality of life: A randomized trial in children with unilateral cerebral palsy. *Developmental Medicine & Child Neurology*, *54*(5), 415-423. <https://doi.org/10.1111/j.1469-8749.2012.04272.x>.

Exclusion reason: 6-18 years

Salie, R., Eken, M. M., Donald, K. A., Fieggen, A. G., C Langerak, N. G. (2022). Pain,

health-related quality of life, and mental health of adolescents and adults with cerebral palsy in urban South Africa. *Disability and rehabilitation*, *44*(17), 4672- 4680. <https://doi.org/10.1080/09638288.2021.1916101>. Exclusion reason: 6-18 years

Sandberg, A. D., C Dahlgren, S. (2012). Theory of mind in children with cerebral palsy: The impact of limited expressive linguistic abilities. In (pp. 62-79). Oxford University Press.

[https://search.ebscohost.com/login.aspx?direct=trueCdb=psyhCAN=2012-](https://search.ebscohost.com/login.aspx?direct=true&db=psyh&AN=2012-06298-004&site=ehost-live) [06298-004Csite=ehost-live](https://search.ebscohost.com/login.aspx?direct=true&db=psyh&AN=2012-06298-004&site=ehost-live). Exclusion reason: Not peer reviewed

Schmidt, S., Markwart, H., Rapp, M., Guyard, A., Arnaud, C., Fauconnier, J., Thyen, U., Hahm, S., Bagazgoitia, N. V. D. E., C Muehlan, H. (2022). Quality of life and mental health in emerging adults with cerebral palsy compared to the general population. *Health and Ǫuality of Life Outcomes*, *20*(1), 61-61.

<https://doi.org/10.1186/s12955-022-01961-7>. Exclusion reason: 6-18 years Schneider, J. A. (2011). *Psychosocial functioning of children with hemiplegic cerebral*

*palsy* ProQuest Information C Learning].

[https://search.ebscohost.com/login.aspx?direct=trueCdb=psyhCAN=2011-](https://search.ebscohost.com/login.aspx?direct=true&db=psyh&AN=2011-99060-179&site=ehost-live) [99060-179Csite=ehost-live](https://search.ebscohost.com/login.aspx?direct=true&db=psyh&AN=2011-99060-179&site=ehost-live). Exclusion reason: Not peer reviewed

Scholtes, V., Vermeer, A., C Meek, G. (2002). Measuring perceived competence and social acceptance in children with cerebral palsy. *European Journal of Special Needs Education*, *17*(1), 77-87. Exclusion reason: 6-18 years

Schuengel, C., Voorman, J., Stolk, J., Dallmeijer, A., Vermeer, A., C Becher, J. (2006). Self-worth, perceived competence, and behaviour problems in children with

cerebral palsy. *Disability and Rehabilitation: An International, Multidisciplinary Journal*, *28*(20), 1251-1258. <https://doi.org/10.1080/09638280600554652>.

Exclusion reason: 6-18 years

Seidel, C., C Tscherner, U. (1982). [Evaluation of the health status of handicapped

persons]. *Z Gesamte Hyg*, *28*(9), 614-616. Exclusion reason: Not able to locate study

Sentenac, M., Rapp, M., Ehlinger, V., Colver, A., Thyen, U., C Arnaud, C. (2021). Disparity of child/parent‐reported quality of life in cerebral palsy persists into

adolescence. *Developmental Medicine & Child Neurology*, *c3*(1), 68-74. <https://doi.org/10.1111/dmcn.14638>. Exclusion reason: 6-18 years

Shahriari, Y., Ghasemzadeh, S., C Vakili, S. (2019). The Effectiveness of Child-Centred Play Therapy on Internalization and Extrapolation Behavioral Problems in Children With Cerebral Palsy. *IRANIAN JOURNAL OF PSYCHIATRY AND CLINICAL PSYCHOLOGY*, *25*(3), 236-248. <https://doi.org/10.32598/ijpcp.25.3.236>.

Exclusion reason: 6-18 years

Sharawat, I. K., Ramachandran, A., Panda, P. K., Kumar, V., C Bhat, N. K. (2023).

Prevalence, severity, and predictors of malnutrition in Indian children with cerebral palsy and their impact on health-related quality of life. *European Journal of Pediatrics*, *182*(5), 2433-2441. Exclusion reason: 6-18 years

Shearer, H. M., Côté, P., Hogg‐Johnson, S., McKeever, P., C Fehlings, D. L. (2022). Pain trajectories and well‐being in children and young people with cerebral palsy: A cohort study. *Developmental medicine and child neurology*, *c4*(11), 1416-1424. <https://doi.org/10.1111/dmcn.15252>. Exclusion reason: 6-18 years

Shelly, A., Davis, E., Waters, E., Mackinnon, A., Reddihough, D., Boyd, R., Reid, S., C Graham, H. K. (2008). The relationship between quality of life and functioning for children with cerebral palsy. *Developmental Medicine & Child Neurology*, *50*(3), 199-203. <https://doi.org/10.1111/j.1469-8749.2008.02031.x>. Exclusion reason: 6-18 years

Shere, E., C Kastenbaum, R. (1966). Mother-child interactions in cerebral palsy: Environmental and psychosocial obstacles to cognitive development. *Genetic Psychology Monographs*. Exclusion reason: Not peer reviewed

Shields, N., Murdoch, A., Loy, Y., Dodd, K. J., C Taylor, N. F. (2006). A systematic review of the self-concept of children with cerebral palsy compared with children without disability. *Dev Med Child Neurol*, *48*(2), 151-157.

<https://doi.org/10.1017/S0012162206000326>. Exclusion reason: Not original study

Shikako-Thomas, K., Dahan-Oliel, N., Shevell, M., Law, M., Birnbaum, R., Rosenbaum, P., Poulin, C., C Majnemer, A. (2012). Play and be happy? Leisure participation and quality of life in school-aged children with cerebral palsy. *International*

*journal of pediatrics*, *2012*. Exclusion reason: 6-18 years

Shikako-Thomas, K., Lach, L., Majnemer, A., Nimigon, J., Cameron, K., C Shevell, M. (2009). Quality of life from the perspective of adolescents with cerebral palsy: 'I just think I’m a normal kid, I just happen to have a disability'. *Ǫuality of Life*

*Research: An International Journal of Ǫuality of Life Aspects of Treatment, Care & Rehabilitation*, *18*(7), 825-832. <https://doi.org/10.1007/s11136-009-9501-3>.

Exclusion reason: 6-18 years

Shrestha, N., Paudel, S., C Thapa, R. (2018). Children with Cerebral Palsy and their Quality Of Life in Nepal. *Journal of Nepal Paediatric Society*, *37*(2), 122-128. <https://doi.org/10.3126/jnps.v37i2.17124>. Exclusion reason: 6-18 years

Siegel, J. H. (2004). *A study of frustration tolerance in cerebral palsied and non- handicapped children* ProQuest Information C Learning].

[https://search.ebscohost.com/login.aspx?direct=trueCdb=psyhCAN=2004-](https://search.ebscohost.com/login.aspx?direct=true&db=psyh&AN=2004-99010-205&site=ehost-live) [99010-205Csite=ehost-live](https://search.ebscohost.com/login.aspx?direct=true&db=psyh&AN=2004-99010-205&site=ehost-live). Exclusion reason: Not peer reviewed

Sienko, S. E. (2018). An exploratory study investigating the multidimensional factors impacting the health and well-being of young adults with cerebral palsy. *Disabil Rehabil*, *40*(6), 660-666. <https://doi.org/10.1080/09638288.2016.1274340>.

Exclusion reason: 6-18 years

Sierra, A. M. (1990). *Ǫuality of attachment and its relationship to competence in infants with cerebral palsy* ProQuest Information C Learning].

[https://search.ebscohost.com/login.aspx?direct=trueCdb=psyhCAN=1990-](https://search.ebscohost.com/login.aspx?direct=true&db=psyh&AN=1990-59497-001&site=ehost-live) [59497-001Csite=ehost-live](https://search.ebscohost.com/login.aspx?direct=true&db=psyh&AN=1990-59497-001&site=ehost-live). Exclusion reason: Not peer reviewed

Silberg, T., Brezner, A., Gal, G., Ahonniska-Assa, J., C Levav, M. (2016). The Role of Maternal Distress in the Report of Behavioral and Emotional Problems among Children with Chronic Disabilities. *ISRAEL JOURNAL OF PSYCHIATRY AND*

*RELATED SCIENCES*, *53*(2), 17-24. Exclusion reason: Mixed sample

Simeoni, M.-C., Schmidt, S., Muehlan, H., Debensason, D., C Bullinger, M. (2007). Field testing of a European quality of life instrument for children and adolescents with chronic conditions: The 37-item DISABKIDS Chronic Generic Module. *Ǫuality of Life Research: An International Journal of Ǫuality of Life Aspects of Treatment,*

*Care & Rehabilitation*, *1c*(5), 881-893. [https://doi.org/10.1007/s11136-007-9188-](https://doi.org/10.1007/s11136-007-9188-2)

[2](https://doi.org/10.1007/s11136-007-9188-2). Exclusion reason: 6-18 years

Sipal, R. F., Schuengel, C., Voorman, J. M., Van Eck, M., C Becher, J. G. (2010). Course of behaviour problems of children with cerebral palsy: The role of parental stress

and support. *Child: Care, Health and Development*, *3c*(1), 74-84.

<https://doi.org/10.1111/j.1365-2214.2009.01004.x>. Exclusion reason: 6-18 years Skjeldal, O. H., Capjon, H., Dahl, A., C Diseth, T. H. (2009). Therapy in a subtropical

climate for children with cerebral palsy Evidence of physical and psychosocial effects? *Acta Paediatrica*, *S8*(4), 670-674. [https://doi.org/10.1111/j.1651-](https://doi.org/10.1111/j.1651-2227.2008.01114.x) [2227.2008.01114.x](https://doi.org/10.1111/j.1651-2227.2008.01114.x). Exclusion reason: 6-18 years

Skoricová, M. (1988). [Psychopathology and clinical aspects of mental disorders in organic brain damage in children]. *Cesk Psychiatr*, *84*(2), 90-99. Exclusion reason: Not able to locate study

Slaman, J., van den Berg-Emons, H. J. G., van Meeteren, J., Twisk, J., van Markus, F.,

Stam, H. J., van der Slot, W. M., C Roebroeck, M. E. (2015). A lifestyle intervention improves fatigue, mental health and social support among adolescents and young adults with cerebral palsy: Focus on mediating effects. *Clinical*

*Rehabilitation*, *2S*(7), 717-727. <https://doi.org/10.1177/0269215514555136>. Exclusion reason: 6-18 years

Sokołowska, E. (1996). Psychologiczne konsekwencje doswiadczeń terapeutycznych matek i ich dzieci / = Psychological consequences of therapeutic experience in mothers and their children. *Psychologia Wychowawcza*, *3S*(3), 241-248. [https://search.ebscohost.com/login.aspx?direct=trueCdb=psyhCAN=1997-](https://search.ebscohost.com/login.aspx?direct=true&db=psyh&AN=1997-04272-004&site=ehost-live)

[04272-004Csite=ehost-live](https://search.ebscohost.com/login.aspx?direct=true&db=psyh&AN=1997-04272-004&site=ehost-live). Exclusion reason: Not able to locate study

Soyupek, F., Aktepe, E., Savas, S., C Askin, A. (2010). Do the self-concept and quality of life decrease in CP patients? Focussing on the predictors of self-concept and

quality of life. *Disability and Rehabilitation: An International, Multidisciplinary Journal*, *32*(13), 1109-1115. <https://doi.org/10.3109/09638280903391120>.

Exclusion reason: 6-18 years

Stahlecker, J. E., C Cohen, M. C. (1985). Application of the strange situation attachment paradigm to a neurologically impaired population. *Child Development*, *5c*(2),

502-507. <https://doi.org/10.2307/1129737>. Exclusion reason: Mixed sample Stokes, T., Mowery, D., Dean, K. R., C Hoffman, S. J. (1997). Nurturance traps of

aggression, depression, and regression affecting childhood illness. In (pp. 147- 154). Westview Press.

[https://search.ebscohost.com/login.aspx?direct=trueCdb=psyhCAN=1997-](https://search.ebscohost.com/login.aspx?direct=true&db=psyh&AN=1997-08237-011&site=ehost-live) [08237-011Csite=ehost-live](https://search.ebscohost.com/login.aspx?direct=true&db=psyh&AN=1997-08237-011&site=ehost-live). Exclusion reason: Not peer reviewed

Sutter, E. N., Francis, L. S., Francis, S. M., Lench, D. H., Nemanich, S. T., Krach, L. E., Sukal-Moulton, T., C Gillick, B. T. (2021). Disrupted Access to Therapies and Impact on Well-Being During the COVID-19 Pandemic for Children With Motor Impairment and Their Caregivers. *Am J Phys Med Rehabil*, *100*(9), 821-830. <https://doi.org/10.1097/PHM.0000000000001818>. Exclusion reason: 6-18 years

Şükran Üneri, Ö., C İkbal Karadavut, K. (2010). Ebeveyn değeriendirmesine dayalı, serebral palsili çocuklarda yaşam kalitesi: Bir ön çalışma = Parent-reported

quality of life of children with cerebral palsy: A Preliminary study. *Nöropsikiyatri Arşivi*, *47*(2), 127-132.

[https://search.ebscohost.com/login.aspx?direct=trueCdb=psyhCAN=2010-](https://search.ebscohost.com/login.aspx?direct=true&db=psyh&AN=2010-15812-008&site=ehost-liveozdenuneri%40yahoo.com) [15812-008Csite=ehost-liveozdenuneri@yahoo.com](https://search.ebscohost.com/login.aspx?direct=true&db=psyh&AN=2010-15812-008&site=ehost-liveozdenuneri%40yahoo.com). Exclusion reason: 6-18 years

Tajik-Parvinchi, D., Davis, A., Roth, S., Rosenbaum, P., Hopmans, S. N., Dudin, A., Hall, G., C Gorter, J. W. (2020). Functional connectivity and quality of life in young adults with cerebral palsy: a feasibility study. *BMC Neurol*, *20*(1), 388.

<https://doi.org/10.1186/s12883-020-01950-7>. Exclusion reason: 6-18 years Tajik‐Parvinchi, D., Farmus, L., Tablon Modica, P., Cribbie, R. A., C Weiss, J. A. (2021).

The role of cognitive control and emotion regulation in predicting mental health problems in children with neurodevelopmental disorders. *Child: Care, Health and Development*, *47*(5), 608-617. <https://doi.org/10.1111/cch.12868>. Exclusion

reason: 6-18 years

Tan, S. S., van Gorp, M., Voorman, J. M., Geytenbeek, J. J. M., Reinders-Messelink, H. A., Ketelaar, M., Dallmeijer, A. J., C Roebroeck, M. E. (2020). Development curves of communication and social interaction in individuals with cerebral palsy.

*Developmental Medicine & Child Neurology*, *c2*(1), 132-139. <https://doi.org/10.1111/dmcn.14351>. Exclusion reason: 6-18 years

Tarsuslu, T., C Livanelioglu, A. (2010). Relationship between quality of life and functional status of young adults and adults with cerebral palsy. *Disability and*

*Rehabilitation: An International, Multidisciplinary Journal*, *32*(20), 1658-1665. <https://doi.org/10.3109/09638281003649904>. Exclusion reason: 6-18 years

Tessier, D. W., Hefner, J. L., C Newmeyer, A. (2014). Factors Related to Psychosocial

Quality of Life for Children with Cerebral Palsy. *International journal of pediatrics*, *2014*(2014), 204386-204386. <https://doi.org/10.1155/2014/204386>. Exclusion

reason: 6-18 years

Tezcan, S., C Simsek, T. T. (2013). Comparison of health-related quality of life between children with cerebral palsy and spina bifida. *Research in Developmental*

*Disabilities*, *34*(9), 2725-2733. <https://doi.org/10.1016/j.ridd.2013.05.017>. Exclusion reason: 6-18 years

Thomas, P. D., Warschausky, S., Golin, R., C Meiners, K. (2008). Direct parenting methods to facilitate the social functioning of children with cerebral palsy. *Journal of Developmental and Physical Disabilities*, *20*(2), 167-174. <https://doi.org/10.1007/s10882-007-9087-z>. Exclusion reason: 6-18 years

Tsoi, W. S. E., Zhang, L. A., Wang, W. Y., Tsang, K. L., C Lo, S. K. (2012). Improving quality of life of children with cerebral palsy: A systematic review of clinical trials. *Child: Care, Health and Development*, *38*(1), 21-31. [https://doi.org/10.1111/j.1365-](https://doi.org/10.1111/j.1365-2214.2011.01255.x) [2214.2011.01255.x](https://doi.org/10.1111/j.1365-2214.2011.01255.x). Exclusion reason: 6-18 years

Turkcan, A. N. (2017). *Effectiveness of dance movement therapy on the quality of gait and socialization of children with cerebral palsy* ProQuest Information C Learning].

[https://search.ebscohost.com/login.aspx?direct=trueCdb=psyhCAN=2017-](https://search.ebscohost.com/login.aspx?direct=true&db=psyh&AN=2017-05719-133&site=ehost-live) [05719-133Csite=ehost-live](https://search.ebscohost.com/login.aspx?direct=true&db=psyh&AN=2017-05719-133&site=ehost-live). Exclusion reason: Not peer reviewed

Tyler, N. B., Kogan, K. L., C Turner, P. (1974). Interpersonal components of therapy with young cerebral palsied. *American Journal of Occupational Therapy*, *28*(7), 395-

400. [https://search.ebscohost.com/login.aspx?direct=trueCdb=psyhCAN=1975-](https://search.ebscohost.com/login.aspx?direct=true&db=psyh&AN=1975-03879-001&site=ehost-live) [03879-001Csite=ehost-live](https://search.ebscohost.com/login.aspx?direct=true&db=psyh&AN=1975-03879-001&site=ehost-live). Exclusion reason: 6-18 years

Türkoğlu, S., Bı̇lgı̇ç, A., Türkoğlu, G., C Yilmaz, S. (2016). Impact of symptoms of

maternal anxiety and depression on quality of life of children with cerebral palsy.

*Nöropsikiyatri Arşivi*, *53*(1), 49-54. <https://doi.org/10.5152/npa.2015.10132>. Exclusion reason: 6-18 years

Tüzün, E. H., Guven, D. K., C Eker, L. (2010). Pain prevalence and its impact on the

quality of life in a sample of Turkish children with cerebral palsy. *Disability and Rehabilitation: An International, Multidisciplinary Journal*, *32*(9), 723-728. <https://doi.org/10.3109/09638280903295433>. Exclusion reason: 6-18 years

Uneri, O. S., C Karadavut, K. I. (2010). Parent-reported quality of life of children with cerebral palsy: a preliminary study/Ebeveyn degerlendirmesine dayali, serebral palsili cocuklarda yasam kalitesi: bir on calisma. *Archives of Neuropsychiatry*, *47*(2), 127-133. Exclusion reason: 6-18 years

Vajda, P., C Čačková, M. (1981). Psychologické problémy detí postihnutých mozgovou obrnou = Psychological problems of children suffering from cerebral palsy.

*Psychológia a Patopsychológia Dieťaťa*, *1c*(5), 446-448.

[https://search.ebscohost.com/login.aspx?direct=trueCdb=psyhCAN=1982-](https://search.ebscohost.com/login.aspx?direct=true&db=psyh&AN=1982-23951-001&site=ehost-live) [23951-001Csite=ehost-live](https://search.ebscohost.com/login.aspx?direct=true&db=psyh&AN=1982-23951-001&site=ehost-live). Exclusion reason: Not able to locate study

van Gilst, C., Prinzie, P., C Vanpeteghem, A. (2016). Externaliserende en

internaliserende gedragsproblemen en kwaliteit van leven bij jongeren met niet- aangeboren hersenletsel = Externalizing and internalizing behaviour problems and quality of life in adolescents with acquired brain injury. *Kind en Adolescent*, *37*(4), 230-245.

[https://search.ebscohost.com/login.aspx?direct=trueCdb=psyhCAN=2016-](https://search.ebscohost.com/login.aspx?direct=true&db=psyh&AN=2016-59093-003&site=ehost-liveprinzie%40fsw.eur.nl) [59093-003Csite=ehost-liveprinzie@fsw.eur.nl](https://search.ebscohost.com/login.aspx?direct=true&db=psyh&AN=2016-59093-003&site=ehost-liveprinzie%40fsw.eur.nl). Exclusion reason: 6-18 years

Vargus-Adams, J. (2005). Health-related quality of life in childhood cerebral palsy. *Archives of physical medicine and rehabilitation*, *8c*(5), 940-945. Exclusion reason: 6-18 years

Vargus‐Adams, J. (2006). Longitudinal use of the Child Health Questionnaire in

childhood cerebral palsy. *Developmental Medicine & Child Neurology*, *48*(5), 343-347. Exclusion reason: 6-18 years

Varni, J. W., Burwinkle, T. M., Sherman, S. A., Hanna, K., Berrin, S. J., Malcarne, V. L., C Chambers, H. G. (2005). Health-related quality of life of children and

adolescents with cerebral palsy: hearing the voices of the children. *Developmental medicine and child neurology*, *47*(9), 592-597. Exclusion reason: 6-18 years

Varni, J. W., Limbers, C. A., C Burwinkle, T. M. (2007). Impaired health-related quality of life in children and adolescents with chronic conditions: a comparative analysis of 10 disease clusters and 33 disease categories/severities utilizing the PedsQL™

4.0 Generic Core Scales. *Health and Ǫuality of Life Outcomes*, *5*, 1-15. Exclusion reason: 6-18 years

Vlčková, I. (2010). Psychologické aspekty a kvalita života dětí a dospívajících s DMO—

Přehledová studie = Psychological aspects and quality of life in children and

adolescents with cerebral palsy—A survey study. *Psychológia a Patopsychológia Dieťaťa*, *45*(4), 328-355.

[https://search.ebscohost.com/login.aspx?direct=trueCdb=psyhCAN=2011-](https://search.ebscohost.com/login.aspx?direct=true&db=psyh&AN=2011-21840-003&site=ehost-liveirena_vlckova%40hotmail.com)

[21840-003Csite=ehost-liveirena_vlckova@hotmail.com](https://search.ebscohost.com/login.aspx?direct=true&db=psyh&AN=2011-21840-003&site=ehost-liveirena_vlckova%40hotmail.com). Exclusion reason: Not able to locate study

Vles, G. F., Hendriksen, R. G., Hendriksen, J. G., van Raak, E. P., Soudant, D., Vles, J. S., C Gavilanes, A. W. (2015). Quality of Life of Children with Cerebral Palsy: A Cross- Sectional KIDSCREEN study in the Southern part of the Netherlands. *CNS Neurol Disord Drug Targets*, *14*(1), 102-109.

<https://doi.org/10.2174/1871527314666150116123045>. Exclusion reason: Not able to locate study

Vles, G. F., Hendriksen, R. G., Vles, J. S., Kessels, A. G., C Hendriksen, J. G. (2012).

Psychosocial adjustment in a Dutch sample of children with cerebral palsy. *Eur J Paediatr Neurol*, *1c*(4), 365-372. <https://doi.org/10.1016/j.ejpn.2011.12.002>.

Exclusion reason: 6-18 years

Volterra, V., C Gamberini, A. (1963). [PSYCHOLOGICAL PREMISES TO THE TREATMENT OF INFANTILE SPASTIC CEREBRAL DISEASES]. *Lattante*, *34*, 441-446. Exclusion

reason: Not able to locate study

Voorman, J. M., Dallmeijer, A. J., Van Eck, M., Schuengel, C., C Becher, J. G. (2010).

Social functioning and communication in children with cerebral palsy: Association with disease characteristics and personal and environmental factors. *Developmental Medicine & Child Neurology*, *52*(5), 441-447.

<https://doi.org/10.1111/j.1469-8749.2009.03399.x>. Exclusion reason: 6-18 years Voyer, A.-P., Nadeau, L., C Tessier, R. (2018). Social dominance in children with cerebral palsy during a problem-solving task with peers. *Disability and Rehabilitation: An*

*International, Multidisciplinary Journal*, *40*(19), 2288-2292.

<https://doi.org/10.1080/09638288.2017.1334237>. Exclusion reason: 6-18 years Vuillerot, C., Hodgkinson, I., Bissery, A., Schott-Pethelaz, A. M., Iwaz, J., Ecochard, R., D'Anjou, M. C., Commare, M. C., C Berard, C. (2010). Self-Perception of Quality

of Life by Adolescents with Neuromuscular Diseases. *Journal of Adolescent Health*, *4c*(1), 70-76. <https://doi.org/10.1016/j.jadohealth.2009.05.005>.

Exclusion reason: 6-18 years

Wadsworth, J. S., C Harper, D. S. (1993). The social needs of adolescents with cerebral palsy. *Developmental Medicine & Child Neurology*, *35*(11), 1019-1022. <https://doi.org/10.1111/j.1469-8749.1993.tb11586.x>. Exclusion reason: 6-18 years

Wake, M., Salmon, L., C Reddihough, D. (2003). Health status of Australian children with mild to severe cerebral palsy: Cross-sectional survey using the Child Health

Questionnaire. *Developmental Medicine & Child Neurology*, *45*(3), 194-199. <https://doi.org/10.1017/S0012162203000379>. Exclusion reason: 6-18 years

Wallander, J. L., C Becker, L. M. (1998). Cognitive status as a risk factor for

maladjustment in children with a physical disability. *Análise Psicológica*, *1c*(1), 77-89.

[https://search.ebscohost.com/login.aspx?direct=trueCdb=psyhCAN=1998-](https://search.ebscohost.com/login.aspx?direct=true&db=psyh&AN=1998-02858-005&site=ehost-live) [02858-005Csite=ehost-live](https://search.ebscohost.com/login.aspx?direct=true&db=psyh&AN=1998-02858-005&site=ehost-live)

Wallander, J. L., Hubert, N. C., C Varni, J. W. (1988). Child and maternal temperament characteristics, goodness of fit, and adjustment in physically handicapped children. *Journal of Clinical Child Psychology*, *17*(4), 336-344.

<https://doi.org/10.1207/s15374424jccp1704_7>

Wallander, J. L., C Varni, J. W. (1989). Social support and adjustment in chronically ill and handicapped children. *American Journal of Community Psychology*, *17*(2), 185-201. <https://doi.org/10.1007/BF00931007>. Exclusion reason: 6-18 years

Wallander, J. L., Varni, J. W., Babani, L., Banis, H. T., C Wilcox, K. T. (1988). Children with chronic physical disorders: Maternal reports of their psychological adjustment. *Journal of Pediatric Psychology*, *13*(2), 197-212.

<https://doi.org/10.1093/jpepsy/13.2.197>. Exclusion reason: 6-18 years Webb, K., Morgan, J., C Lacey, J. H. (2009). Cerebral Palsy and Anorexia Nervosa.

*INTERNATIONAL JOURNAL OF EATING DISORDERS*, *42*(1), 87-89.

<https://doi.org/10.1002/eat.20559>. Exclusion reason: 6-18 years Weber, P., Bolli, P., Heimgartner, N., Merlo, P., Zehnder, T., C Kätterer, C. (2016).

Behavioral and emotional problems in children and adults with cerebral palsy.

*Eur J Paediatr Neurol*, *20*(2), 270-274. <https://doi.org/10.1016/j.ejpn.2015.12.003> Westaway, J. L. (1992). The assessment and management of emotional disorder in

cerebral palsied children. *Southern African Journal of Child & Adolescent Psychiatry*, *4*(2), 53-59. <https://doi.org/10.1080/16826108.1992.9631488>. Exclusion reason: Not original study

White-Koning, M., Arnaud, C., Dickinson, H. O., Thyen, U., Beckung, E., Fauconnier, J., McManus, V., Michelsen, S. I., Parkes, J., Parkinson, K., Schirripa, G., C Colver, A. (2007). Determinants of child-parent agreement in quality-of-life reports: A European study of children with cerebral palsy. *Pediatrics*, *120*(4), e804-e814. <https://doi.org/10.1542/peds.2006-3272>. Exclusion reason: 6-18 years

White-Koning, M., Grandjean, H., Colver, A., C Arnaud, C. (2008). Parent and professional reports of the quality of life of children with cerebral palsy and associated intellectual impairment. *Dev Med Child Neurol*, *50*(8), 618-624.

<https://doi.org/10.1111/j.1469-8749.2008.03026.x>. Exclusion reason: 6-18 years

Whitney, D. G., Peterson, M. D., C Warschausky, S. A. (2019). Mental health disorders, participation, and bullying in children with cerebral palsy. *Developmental Medicine & Child Neurology*, *c1*(8), 937-942.

<https://doi.org/10.1111/dmcn.14175>. Exclusion reason: 6-18 years

Whitney, D. G., Shapiro, D. N., Peterson, M. D., C Warschausky, S. A. (2019). Factors

associated with depression and anxiety in children with intellectual disabilities.

*Journal of Intellectual Disability Research*, *c3*(5), 408-417.

<https://doi.org/10.1111/jir.12583>. Exclusion reason: Population not CP Whitney, D. G., Warschausky, S. A., C Peterson, M. D. (2019). Mental health disorders

and physical risk factors in children with cerebral palsy: A cross‐sectional study. *Developmental Medicine & Child Neurology*, *c1*(5), 579-585. Exclusion reason: 6- 18 years <https://doi.org/10.1111/dmcn.14083>. Exclusion reason: Population not CP

Whittingham, K., Bodimeade, H. L., Lloyd, O., C Boyd, R. N. (2014). Everyday psychological functioning in children with unilateral cerebral palsy: Does

executive functioning play a role? *Developmental Medicine & Child Neurology*, *5c*(6), 572-579. <https://doi.org/10.1111/dmcn.12374>

Whittingham, K., Sanders, M., McKinlay, L., C Boyd, R. N. (2014). Interventions to reduce behavioral problems in children with cerebral palsy: An RCT. *Pediatrics*, *133*(5), e1249-e1257. <https://doi.org/10.1542/peds.2013-3620>. Exclusion reason: 6-18 years

Whittingham, K., Wee, D., C Boyd, R. (2011). Systematic review of the efficacy of parenting interventions for children with cerebral palsy. *Child: Care, Health and Development*, *37*(4), 475-483. [https://doi.org/10.1111/j.1365-](https://doi.org/10.1111/j.1365-2214.2011.01212.x)

[2214.2011.01212.x](https://doi.org/10.1111/j.1365-2214.2011.01212.x). Exclusion reason: Not original study

Wiley, R., C Renk, K. (2007). Psychological correlates of quality of life in children with cerebral palsy. *Journal of Developmental and Physical Disabilities*, *1S*(5), 427-

447. <https://doi.org/10.1007/s10882-007-9041-0>. Exclusion reason: 6-18 years Williams, K., Jacoby, P., Whitehouse, A., Kim, R., Epstein, A., Murphy, N., Reid, S.,

Leonard, H., Reddihough, D., C Downs, J. (2021). Functioning, participation, and quality of life in children with intellectual disability: An observational study.

*Developmental Medicine & Child Neurology*, *c3*(1), 89-96. <https://doi.org/10.1111/dmcn.14657>. Exclusion reason: 6-18 years

Withers, J. W., Muzzolon, S. B., C Zonta, M. B. (2019). Influence of adapted hip hop dancing on quality of life and social participation among children/adolescents with cerebral palsy. *ARǪUIVOS DE NEURO-PSIǪUIATRIA*, *77*(10), 712-722.

<https://doi.org/10.1590/0004-282X20190124>. Exclusion reason: 6-18 years Yamaguchi, R., Perry, K. N., C Hines, M. (2014). Pain, pain anxiety and emotional and

behavioural problems in children with cerebral palsy. *Disability and*

*Rehabilitation: An International, Multidisciplinary Journal*, *3c*(2), 125-130. <https://doi.org/10.3109/09638288.2013.782356>. Exclusion reason: 6-18 years

Yang, P., Chen, Y.-H., Yen, C.-F., C Chen, H.-L. (2015). Psychiatric diagnoses, emotional– behavioral symptoms and functional outcomes in adolescents born preterm

with very low birth weights. *Child Psychiatry and Human Development*, *4c*(3), 358-366. <https://doi.org/10.1007/s10578-014-0475-1>. Exclusion reason: 6-18 years

Young, B., Rice, H., Dixon-Woods, M., Colver, A. F., C Parkinson, K. N. (2007). A qualitative study of the health-related quality of life of disabled children. *Developmental Medicine & Child Neurology*, *4S*(9), 660-665.

<https://doi.org/10.1111/j.1469-8749.2007.00660.x>. Exclusion reason: 6-18 years Young, N. L., Rochon, T. G., McCormick, A., Law, M., Wedge, J. H., C Fehlings, D. (2010).

The health and quality of life outcomes among youth and young adults with cerebral palsy. *Archives of physical medicine and rehabilitation*, *S1*(1), 143-148. Exclusion reason: 6-18 years

Yu, H., Liu, Y., Li, S., C Ma, X. (2009). Effects of music on anxiety and pain in children with cerebral palsy receiving acupuncture: A randomized controlled trial.

*International Journal of Nursing Studies*, *4c*(11), 1423-1430.

<https://doi.org/10.1016/j.ijnurstu.2009.05.007>. Exclusion reason: 6-18 years Zhelezniak, V. (1999). *Medical staff: Child interaction during a painful therapeutic*

*procedure* ProQuest Information C Learning].

[https://search.ebscohost.com/login.aspx?direct=trueCdb=psyhCAN=1999-](https://search.ebscohost.com/login.aspx?direct=true&db=psyh&AN=1999-95006-454&site=ehost-live) [95006-454Csite=ehost-live](https://search.ebscohost.com/login.aspx?direct=true&db=psyh&AN=1999-95006-454&site=ehost-live). Exclusion reason: Not peer reviewed

Zuculo, G. M., Knap, C. C., C Pinato, L. (2014). Correlation between sleep and quality of life in cerebral palsy. *Codas*, *2c*(6), 447-456. [https://doi.org/10.1590/2317-](https://doi.org/10.1590/2317-1782/20140201435)

[1782/20140201435](https://doi.org/10.1590/2317-1782/20140201435). Exclusion reason: 6-18 years

Özer, D., Nalbant, S., Aktop, A., Duman, Ö., Keleş, İ., C Toraman, N. F. (2007). Swimming training program for children with cerebral palsy: Body perceptions, problem

behaviour, and competence. *Perceptual and Motor Skills*, *105*(3, Pt1), 777-787. <https://doi.org/10.2466/PMS.105.7.777-787>. Exclusion reason: 6-18 years
