# Supplementary material for: Socio‐Emotional Development in Young Children With Cerebral Palsy: A Scoping Review
Source: Child Care Health Dev. 2025 Jul 2;51(4):e70130. doi: 10.1111/cch.70130 (PMC12223170; doi:10.1111/cch.70130)
Supplement: Supplementary file 4 — Data S4 Sources of funding and conflict of interest of the included studies. [file CCH-51-e70130-s004.docx]

Online supplementary material 4

Sources of funding and conflict of interest of the included studies

| **Study** | **Sources of funding and conflict of interest** |
| --- | --- |
| Alsem et al., 2013 | The authors report no declaration of interest. The PERRIN CP 0-5 study was supported by ZonMw, the Netherlands Organization for Health Research and Development (grant No.1435.0011). No  additional funding was received. |
| Banham, 1973 | N/a |
| Brooks-Gunn &  Lewis, 1984 | N/a |
| Burgess et al., 2023 | National Health and Medical Research Council, Grant/Award Number: Australian CP Child StudyNHMRC 465128, NHMRC CDF1 Fellowship (LS 1160694), NHMRC Research Fellowship (RB 1037220) and Predict-CP study NHMRC 1077257; Ramaciotti Health Investment Grant, Grant/Award Number: Ramaciotti Health Investment Grant; University of Queensland, Grant/Award  Number: UQ Research Stimulus (AB) |
| Chang et al., 2012 | No commercial party having a direct financial interest in the results of the research supporting this article has or will confer a benefit upon the authors or upon any organization with which the authors  are associated. |
| Chen et al., 2011 | The authors would like to thank the Health Administration of Executive Yuan, Taiwan, for financially supporting this research  under Contract No. GMRPG360101. |

| Chen et al., 2013 | No party has direct interest neither a financial relationship nor will confer a benefit from the results of this research. The National Science Council (NSC 98-2314-B-182-006-MY3) of Taiwan and Chang Gung Memorial Hospital (CMRPG 391671-3) financially  supported this research. |
| --- | --- |
| Eisenhower et al., 2005 | This article is based on the activities of the Collaborative Family Study, supported by NICHD grant 34879-1459 [Dr Keith Crnic, Principal Investigator (PI), and Drs Bruce L. Baker, Jan Blacher  and Craig Edelbrock, co-PI]. |
| Fuertes et al., 2003 | O presente trabalho foi financiado pela FCT, programa CPSI-50, Centro de Psicologia, linha nº 3./ This work was funded by FCT,  program CPSI-50, Psychology Center, line no. 3. |
| Gehrmann et al., 2014 | Funding was provided by a National Health and Medical Research Council of Australia Project Grant (569605). R. Boyd is supported by a National Health and Medical Research Council Career Development Fellowship (1037220). The authors have stated that they had no interests that might be perceived as posing a conflict or  bias |
| Horwood et al., 2019 | Dr. Horwood’s doctoral work on the study was funded in part by awards from the Fonds de recherche du Québec-Santé (FRQ-S), the Kids Brain Health Network (KBHN), the Research Institute of the McGill University Health Care Centre (RI-MUHC) and McGill University. Dr. Li holds grants from the FRQ-S, the RI-MUHC and the Canadian Institutes of Health Research (CIHR). Dr. Oskoui  holds grants from the FRQ-S, CIHR, the Cerebral Palsy Alliance |

|  | Foundation, and KBHN. Dr. Shevell holds grants from the KBHN. Dr. Constantin holds grants from the KBHN, CIHR and RI-MUHC. The RI-MUHC is supported in part by the FRQ-S. The Cerebral Palsy Registry is funded by the FRQ-S and the KBHN. Declaration  of Competing Interest: None. |
| --- | --- |
| Keawutan et al., 2018 | National Health and Medical Research Council (NHMRC) for Project Grant (569605) ‘Queensland CP Child Study of Growth, Nutrition and Physical Activity’; NHMRC for Project Grant (465128) ‘Queensland CP Child Study of Motor Function and Brain Development’; Thammasat University PhD. Scholarship (PK); Australian Postgraduate Award Scholarship (SO);  Queensland Children’s Medical Research Institute PhD Scholarship |
| Konst et al., 2014 | (SO); Smart State Fellowship from Queensland Government (RB); NHMRC Research Fellowship, no 1105038. Funding was not involving in study design, data collection and data analysis, manuscript preparation and publication decisions. The authors report no conflicts of interest.  The authors report no conflicts of interests and are solely  responsible for the content and writing of this paper. |
| Lai et al., 2015 | This work was supported by the Ministry of Science and Technology, Taiwan (NSC 101-2314-B-182-004-MY3, NSC 102-  2410-H-182-018) and Chang Gung Memorial Hospital, Taiwan  (CMRPG 391671-3, CMRPG3D1271-3). |
| Lipscombe et al.,  2016 | This work was supported by National Health and Medical Research  Council project grants (348400; 569605), a National Health and |

|  | Medical Research Council Postdoctoral Fellowship (K.W., grant number 631712); a National Health and Medical Research Council Career Development Fellowship (R.B., grant number 1037220) and a Smart State Fellowship (R.B). The authors have no conflicts of  interest to disclose. |
| --- | --- |
| Marvin & Pianta,  1996 | This research was supported by NICHD Grant R01 HD 26911 and  NIDRR Grant H133G20118 |
| Moraleda-Barreno & Romero, 2011 | Al Convenio Específico de Colaboración entre el Hospital Juan Ramón Jiménez, la Universidad de Huelva y la Fundación Andaluza Beturia para la Investigación en Salud. Al profesor Pedro Pérez Moreno en los aspectos metodológicos, al personal del Servicio de Rehabilitación del hospital y a los ninos que han participado en esta investigación. Los autores declaran no tener ningún conflicto de intereses. / To the Specific Collaboration Agreement between the Juan Ramón Jiménez Hospital, the University of Huelva, and the Andalusian Beturia Foundation for Health Research. The authors declare that they have no conflict of  interest. |
| Romeo et al., 2014 | The ICMJE Uniform Disclosure Form for Potential Conflicts of Interest associated with this article can be viewed by clicking on  the following link: [http://dx.doi.org/10.1016/j.sleep.2014.05.008.](http://dx.doi.org/10.1016/j.sleep.2014.05.008) |
| Sharawat & Panda,  2022 | Conflict of Interest: None declared. |

| Sigurdardottir et al., 2010 | The study was funded by the Liaison Committee for Central  Norway Regional Health Authority and the Norwegian University of Science and Technology. |
| --- | --- |
| Sipes et al., 2011 | The authors report no conflicts of interests. The authors alone are  solely responsible for the content and writing of the paper. |
| Tan et al., 2014 | This research has been performed as part of the Pediatric Rehabilitation Research in The Netherlandsþ (PERRINþ) research program. Supported by the Stichting Rotterdams Kinderrevalidatie Fonds Adriaanstichting and Stichting Johanna KinderFonds (grant  no. 2010/0040). Disclosures: none |
| Wanamaker &  Glenwick, 1998 | N/a |
| Whittingham et al., 2010 | This research was supported by an NHMRC Grant (368400). KW acknowledges an NHMRC postdoctoral fellowship (631712). RB acknowledges a NHMRC Career Development Award (473840)  and a Smart State Fellowship. |
| Wu et al., 2021 | This study was supported by the General Program of the National Natural Science Foundation of China (Grant NO. 81371900). We  wish to confirm that all of the authors report no conflicts of interest. |
